# Untargeted Metabolomics Approach for the Discovery of Environment-Related Pyran-2-Ones Chemodiversity in a Marine-Sourced *Penicillium restrictum*

**DOI:** 10.3390/md19070378

**Published:** 2021-06-29

**Authors:** Van-Tuyen Le, Samuel Bertrand, Thibaut Robiou du Pont, Fabrice Fleury, Nathalie Caroff, Sandra Bourgeade-Delmas, Emmanuel Gentil, Cedric Logé, Gregory Genta-Jouve, Olivier Grovel

**Affiliations:** 1 Faculty of Pharmacy, University of Nantes, EA 2160-Mer Molécules Santé, 9 rue Bias BP 53508, CEDEX 1, 44035 Nantes, France; van-tuyen.le1@univ-nantes.fr (V.-T.L.); samuel.bertrand@univ-nantes.fr (S.B.); thibaut.robiou@univ-nantes.fr (T.R.d.P.); emmanuel.gentil@univ-nantes.fr (E.G.); 2 Group of Mechanism and Regulation of DNA Repair and IMPACT Platform, UFIP UMR CNRS 6286, University of Nantes, 44322 Nantes, France; Fabrice.Fleury@univ-nantes.fr; 3 Laboratoire EA3826, University of Nantes, IRS2 Nantes Biotech, CEDEX 1, 44100 Nantes, France; nathalie.caroff@univ-nantes.fr; 4 UMR 125 PharmaDev, University of Toulouse, IRD, 31400 Toulouse, France; sandra.bourgeade-delmas@ird.fr; 5 EA1155-IICIMED, University of Nantes, IRS2 Nantes Biotech, CEDEX 1, 44100 Nantes, France; Cedric.Loge@univ-nantes.fr; 6 Laboratory Ecology, Evolution, Interactions of Amazonian Systems (LEEISA), USR 3456, University of French Guiana, CNRS Guyane, 275 Route de Montabo, 97334 Cayenne, France; gregory.genta-jouve@cnrs.fr

**Keywords:** *Penicillium restrictum*, mussel-derived fungi, OSMAC, metabolome, pyran-2-one

## Abstract

Very little is known about chemical interactions between fungi and their mollusc host within marine environments. Here, we investigated the metabolome of a *Penicillium restrictum* MMS417 strain isolated from the blue mussel *Mytilus edulis* collected on the Loire estuary, France. Following the OSMAC approach with the use of 14 culture media, the effect of salinity and of a mussel-derived medium on the metabolic expression were analysed using HPLC-UV/DAD-HRMS/MS. An untargeted metabolomics study was performed using principal component analysis (PCA), orthogonal projection to latent structure discriminant analysis (O-PLSDA) and molecular networking (MN). It highlighted some compounds belonging to sterols, macrolides and pyran-2-ones, which were specifically induced in marine conditions. In particular, a high chemical diversity of pyran-2-ones was found to be related to the presence of mussel extract in the culture medium. Mass spectrometry (MS)- and UV-guided purification resulted in the isolation of five new natural fungal pyran-2-one derivatives—5,6-dihydro-6*S*-hydroxymethyl-4-methoxy-2*H*-pyran-2-one (**1**), (6S, 1’*R*, 2’*S*)-LL-P880*β* (**3**), 5,6-dihydro-4-methoxy-6*S*-(1’*S*, 2’*S*-dihydroxy pent-3’(*E)*-enyl)-2*H*-pyran-2-one (**4**), 4-methoxy-6-(1’*R*, 2’*S*-dihydroxy pent-3’(*E)*-enyl)-2*H*-pyran-2-one (**6**) and 4-methoxy-2*H*-pyran-2-one (**7**)—together with the known (6S, 1’*S*, 2’*S*)-LL-P880*β* (**2**), (1’*R*, 2’*S*)-LL-P880*γ* (**5**), 5,6-dihydro-4-methoxy-2*H*-pyran-2-one (**8**), (6*S*, 1’*S*, 2’*R)*-LL-P880*β* (**9**), (6*S*, 1’*S*)-pestalotin (**10**), 1’*R*-dehydropestalotin (**11**) and 6-pentyl-4-methoxy-2*H*-pyran-2-one (**12**) from the mussel-derived culture medium extract. The structures of **1**-**12** were determined by 1D- and 2D-MMR experiments as well as high-resolution tandem MS, ECD and DP4 calculations. Some of these compounds were evaluated for their cytotoxic, antibacterial, antileishmanial and in-silico PTP1B inhibitory activities. These results illustrate the utility in using host-derived media for the discovery of new natural products.

## 1. Introduction

The phylum Mollusca is one of the largest and most diverse group in the animal kingdom. Estimated at 30,000 species in the sea, it comprises various bivalves such as oysters, mussels and clams [1]. As filter feeders, these shellfish can actively retain and concentrates particles and micro-organisms from their surrounding environment such as bacteria [2], viruses [3,4], protozoa [5], and fungi [6,7,8]. Zvereva et al. reported that fungal strains belonging to *Aspergillus*, *Penicillium*, *Acremonium*, and *Alternaria* genera are the most predominantly found in the two bivalve molluscs *Crenomytilus grayanus* and *Modiolus modiolus* [7]. Santos et al. reported eight fungal genera, such as *Aspergillus*, *Penicillium,* and *Fusarium* from the gills, intestine, and muscle tissue of the *Nodipectennodosus* scallop from marine farms in Brazil [8]. Sallenave-Namont et al. reported the presence of genera *Penicillium*, *Trichoderma*, *Clasdosporium*, *Aspergillus*, *Paecilomyces* along with Mucorales from mussel samples from marine shellfish farming areas [6]. In this way, all marine bivalves are colonized by a high diversity of microorganisms and fungi are major contributors to the microbiome of these holobionts. Various lifestyles such as symbiosis, parasitism, and mutualism have been described for fungi, depending on the species and the host [9,10]. In these ecosystems, fungi can establish numerous interactions with their hosts, mediated by secondary metabolites that serve as communication or chemical war purposes [10]. However, while the relationship between plants and endophytic fungi or between animals and fungal parasites have been the object of numerous works, almost no studies have investigated the chemical ecology of the association between mollusc bivalves and associated fungi.

*Penicillium* is one of the predominant genera in marine environments [11] and shellfish-derived *Penicillium* strains have been demonstrated to produce a high range of metabolites. Some of these are identical to compounds from terrestrial origin such as roquefortine C [12], patulin, cladosporin, festuclavin or griseofulvin [13,14], but others have been first detected from marine strains [10]. It seems that under the very specific conditions observed in marine environments, such as pressure, salinity or tides, some chemical pathways or chemical conditions are expressed that are not observed in terrestrial media. An interaction with marine invertebrates is one further condition that can be supposed to induce a dedicated chemistry. In this way, in a previous work, it was demonstrated that *Penicillium* strains isolated from shellfish produced more bioactive compounds than strains sampled from their surrounding environment [15,16].

To access metabolites involved in the chemical communication between the two species and whose expression is silenced under usual laboratory culture conditions, culture-based strategies such as OSMAC (One Strain Many Compounds) are mandatory [17,18]. As part of this strategy, in vitro cultivation of fungi in the presence of host-derived substrates can induce specific metabolites. For example, the cultivation of a *Penicillium hordei* strain on tulip agar led to the medium-dependant production and isolation of the novel metabolites corymbiferone [19] and corymbiferan lactones A-D [20]. Host-derived media have also been successfully applied to enhance the yield of fungal inocula [21,22], to stimulate the production of low abundance metabolites [23], and to promote novel extracellular enzyme production and enhance protein expression [24,25,26,27]. In the marine field, the in vitro cultivation of fungi on mussel-derived substrates has been employed to convert agricultural and marine residues into microbial metabolites [27,28,29,30,31,32], and a recent study showed that mussel-processing wastewaters were a promising nutritive medium for astaxanthin production by the basidiomycetous species *Xanthophyllomyces dendrorhous* [29]. However, very little information is available on fungal metabolome expression induced by bivalves. Only one study has shown that the use of a mussel flesh-derived culture medium enhanced the production of cytotoxic metabolites by some mussel-derived fungi [16].

During our ongoing search for new marine fungal natural products, a *Penicillium restrictum* J.C. Gilman & E.V. Abbott strain was isolated from a mussel sample in the Loire estuary, France. Although it is mainly considered as a typical terrestrial soil species, it has also been sampled from seawater, corals and marine sponges [33]. *Penicillium restrictum* isolates have been shown to produce some bioactive metabolites such as dehydrocarolic acid, gliotoxin [34], restricticin and its dimethyl derivative [35,36], curvularins [37], calbistrins [38,39], and the mycotoxins patulin and penicillic acid [40].

In this study, we present a metabolome investigation of a mussel-derived strain of *P. restrictum* MMS417, with a focus on environment-derived culture conditions. Alterations of culture conditions were performed following the OSMAC approach using seven culture media including a host-derived medium to evaluate the influence of mussel components on the production of specialized metabolites. In addition, the effect of salinity was also explored. Culture extracts were submitted to an untargeted metabolomics study using UPLC-IT/ToF–MS/MS-based molecular networking (MN) [41]. This resulted in highlighting some classes of metabolites overexpressed by the presence of mussel and seawater and to the MS-guided isolation of 12 pyran-2-ones including five new fungal natural products. Some of these compounds were tested for cytotoxic, antibacterial, antileishmanial activity and virtual PTP1B inhibition.

## 2. Results

### 2.1. Effects of Culture Medium Components on the Metabolome of P. restrictum

To explore the chemical diversity produced by *P. restrictum* MMS417 according to culture media, a complete metabolomics study was performed following the OSMAC approach. The strain was grown over six usual agar-based media—Czapek Yeast Agar (CYA), Dextrose Casein Agar (DCA), Potato Dextrose Agar (PDA), Yeast Extract Sucrose agar (YES), Malt Extract Agar (MEA) and Kohlmeyer-Medium Solid (KMS). A seventh medium was employed: Mussel Extract Sucrose Agar (MES), a host-derived medium [16] used to evaluate the influence of mussel flesh on the production of specific metabolites. Two different osmotic conditions were also used separately for each medium, by employing either distilled water (DW) or synthetic seawater (SSW) for the reconstitution of the media. Colony morphology was observed throughout the culture growth (Figure S1), showing that the morphology of the strain was dramatically modified according to each medium. A particularly unusual aspect was observed for cultures in the MES-SSW medium, which appeared to be “wet” and “submerged” at the centre of the colony with an absence of classical aerial mycelial structures.

**Figure 1:**
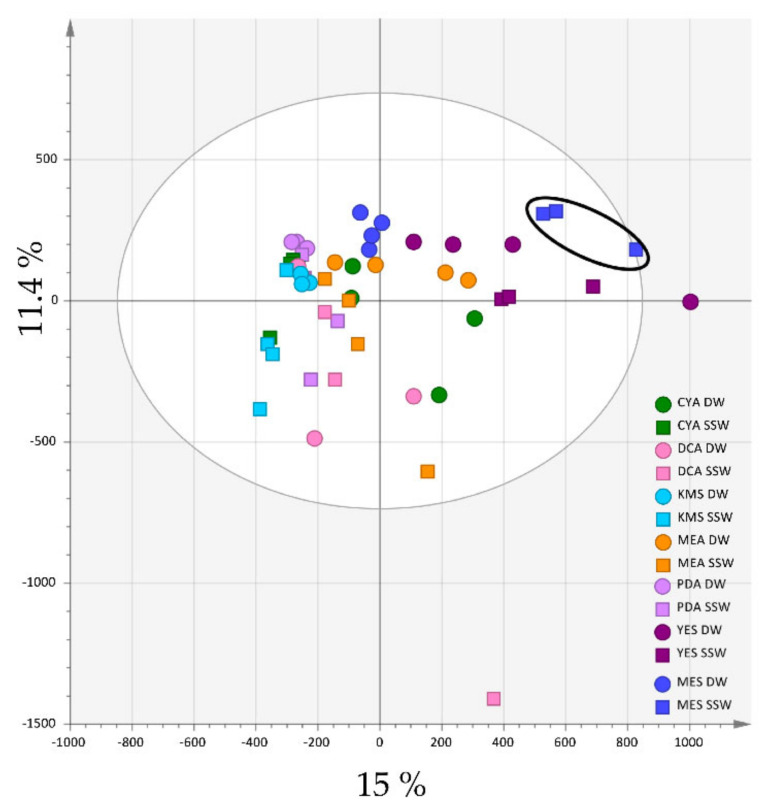
PCA (Pareto scaling) of the 14 *Penicillium restrictum* metabolic profiles (HPLC-HRMS data in positive ionization mode, n = 4). MES-SSW extracts are highlighted by a black circle.

Extracts of the cultures after 10 days were analysed by HPLC-HRMS using both the positive and negative ionisation modes. The chemical profiles of the 14 types of cultures (four replicates each) were converted to a data matrix using automated peak detection and compared by multivariate data analyses. The data were preliminarily analysed by principal component analysis (PCA) including quality control samples (QC), which confirmed data consistency (Pareto scaling) (Figure S2). In the positive ionization mode, the PCA scores plot achieved on all the 56 samples (Figure 1) showed a variation in the chemical profiles, with the first two components of PCA explaining 26.4% of the variance (15 % and 11.4 % for PC1 and PC2, respectively).

It clearly appeared that both the medium composition and the presence of seawater influenced the extract composition. Extracts from media with the lowest content in nutrients (KMS, PDA and DCA) clustered closely together on the left part of the scores plot, while YES and MES extracts, issued from media for which the composition was similar except for the presence of mussel flesh in MES instead of yeast extract on YES, clustered in the other same part of the scores plot. On the other hand, DW and SSW extracts were separated from each other regardless of the composition of the medium. Of particular interest was the observation that MES-SSW extracts appeared clearly individualized from the other ones, suggesting that environment-based culture conditions can induce the production of specific compounds.

The influence of sea water/distilled water on the induction/repression of the production of metabolites was then globally investigated. For that purpose, a supervised orthogonal projection to latent structure discriminant analysis (OPLS-DA), comparing the two osmotic conditions was performed to highlight the distribution differences of metabolites in DW or SW extracts.

**Figure 2:**
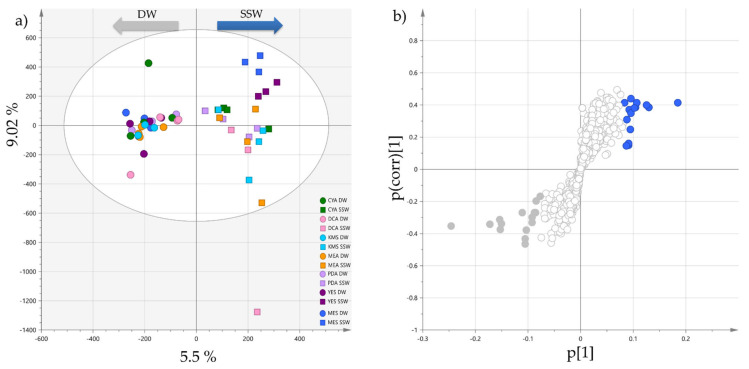
(**a**) OPLS-DA of the 14 *Penicillium restrictum* metabolic profiles (HPLC-HRMS data in positive ionization mode, n = 4) with separation of extracts following salinity (R2X cum 0.316 and R2Y cum 0.89, Q2 cum 0.766; CV-ANOVA (*p*-value = 5.61 × 10^−11^)); (**b**) corresponding S-plot showing regions (blue: SSW; grey: DW) of highly specific features following salinity.

The OPLS-DA (Figure 2a) showed significant discrimination between the two groups along the first dimension, and that the DW extracts metabolic profiles were less dispersed than for the SSW extracts on the orthogonal component. This was particularly obvious for MES-SSW and YES-SSW profiles which clustered separately from the other SSW extracts. They were also the most influenced by the type of water used. The S-loadings plot represented in Figure 2b highlighted the metabolites distinct between the two groups. Features with a large Variable Influence on Projection values (VIP > 2) were considered to be the most contributing ones and were subjected to dereplication based on their (+)-HRESIMS spectrum, UV-vis spectrum and retention time leading to their putative annotation (Table 1). Nevertheless, many annotations could not be ascertained, as regioisomers and stereoisomers that can be produced simultaneously by the same fungal strain were described in the literature. Thus, these isobaric compounds were annotated as putative isomers of a reference compound already described from fungi. Most of the VIP compounds were found to correspond to some pyran-2-one derivatives, which could be clearly annotated due to the characteristic UV absorption of either the pyran-2-one (λ_max_ = 280 nm) [42] or the 5,6-dihydropyran-2-one moieties (λ_max_ = 238 nm) [43]. In SSW extracts, pestalotin and 2’-hydroxypestalotin isomers [44,45] were highly produced, whereas dehydropestalotin, LL-P880*γ* [46], and 5,6-dihydro-4-methoxy-2*H*-pyran-2-one [45] were correlated with DW culture conditions. Various steroid derivatives were also detected as compounds distinctive of SSW extracts with antibiotic Mer-NF 8054A [47], 3*β*-hydroxyergosta-8,14,24(28)-trien-7-one [48] and paxisterol [49], together with the two macrolides melearoride A [50] and *N*-demethylmelearoride A [51]. Among all the metabolites detected and annotated, only antibiotic TAN-1446A has been previously described from *P. restrictum* [52]. Some unidentified metabolites at *m*/*z* 412.3335 [M+H]^+^ (Rt = 23.28 min) and *m*/*z* 427.3221 [M+H]^+^ (Rt = 23.76 min) were also highlighted in SSW profiles, which did not match to any compound in natural product databases (see Materials and Methods).

**Figure 3:**
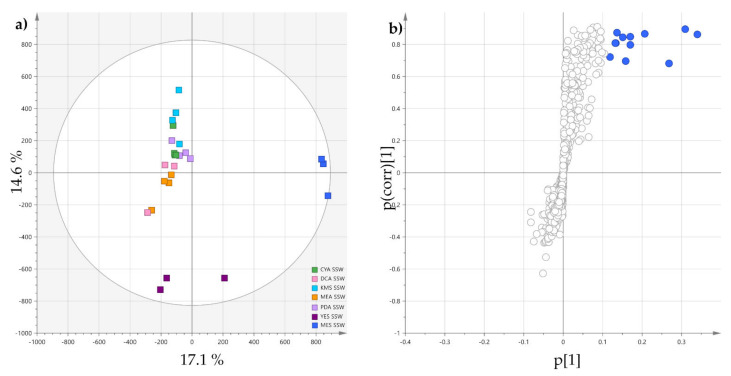
(**a**) OPLS-DA scores-plot of MES-SSW extracts versus the six other SSW extracts (positive ionization, n = 4; R2X cum 0.317 and R2Y cum 0.927, Q2 cum 0.777; CV-ANOVA (*p*-value = 2.72 × 10^−6^)); (**b**) corresponding S-plot: features highlighted in blue correspond to discriminatory ions with VIP > 3.

To observe the specific effect of the mussel flesh on the induction of metabolites in *P. restrictum*, an OPLS-DA was carried out comparing the MES-SSW extracts to other SSW extracts (Figure 3). The validated OPLS-DA model (Figure 3, R2Y cum 0.89, Q2 cum 0.766) exhibited a significant discrimination between the two groups following the first component, the MES-SSW extracts being well separated from the others. The S-plot showed that the ions responsible for distinction of MES-SSW (VIP > 3) were still pyran-2-one derivatives: isomers of pestalotin, 2’-hydroxypestalotin, dehydroxypestalotin and LL-P880*γ* were dereplicated among the compounds with the highest VIP values (Table 2). The ion at *m*/*z* 211.0581 [M+Na]^+^ eluted at 6.58 min remained unidentified when searched in fungal natural products databases. However, its UV-Vis spectrum showed an absorption band at λ_max_ = 237 nm, which is characteristic of a 5,6-dihydropyran-2-one moiety [43]. The compound at *m*/*z* 147.0654 was annotated as 5-hydroxy-3-methoxy-2-pentenoic acid (verrucolone or arabenoic acid), which corresponds to an open acidic form of 5,6-dihydro-4-methoxy-2*H*-pyran-2-one, two compounds which have been previously described from *Penicillium* strains [53,54]. From this OPLS-DA, it could also be noticed that on the first orthogonal component, which corresponds to an unsupervised direction, the YES-SSW extracts were clearly distinguished from the other SSW extracts. Another OPLS-DA (Figure S3, Table S1) was conducted comparing these extracts to other SSW extracts, which showed that other pyran-2-ones together with melearorides were responsible for this discrimination.

### 2.2. Molecular Networking of P. restrictum MES-SSW Extract

**Figure 4:**
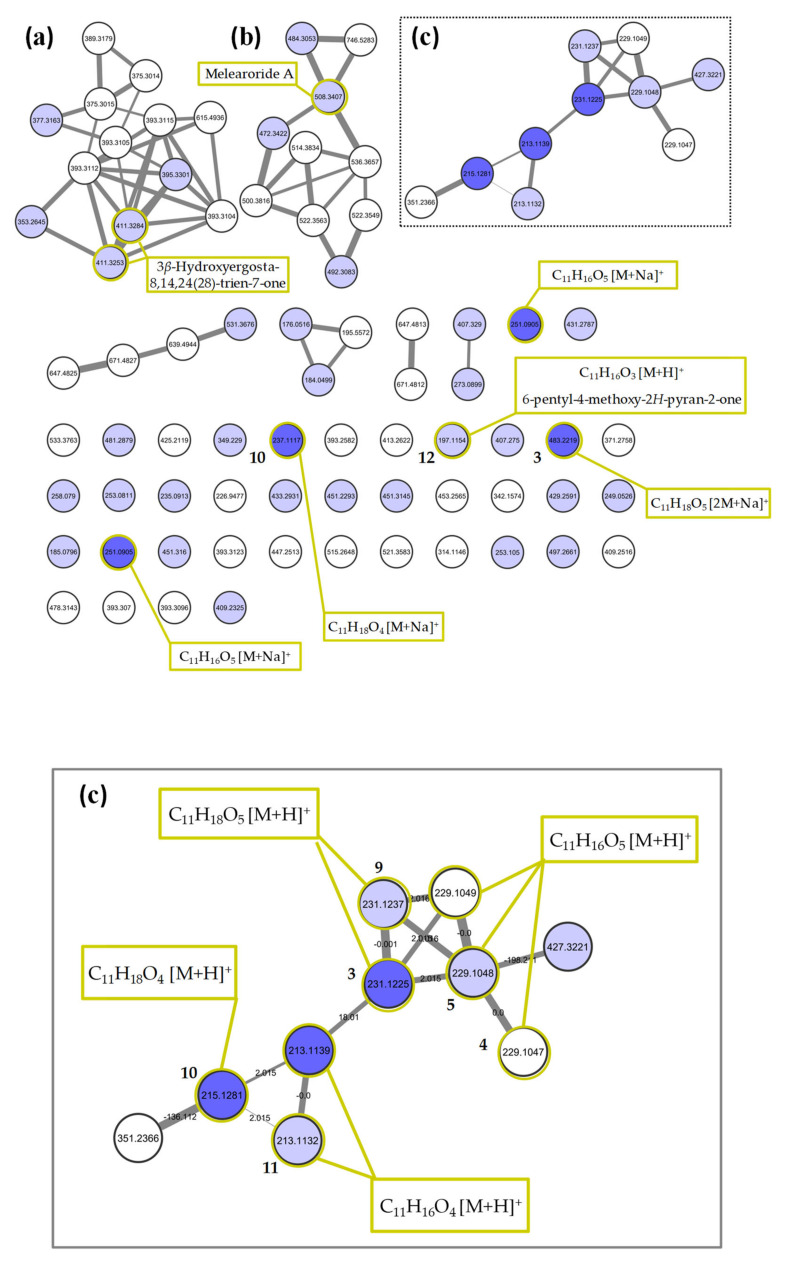
MS/MS molecular network obtained from the MES-SSW extract of *Penicillium restrictum* MMS417. Above: complete network showing the three main clusters, (**a**) ergosterol derivatives, (**b**) melearorides derivatives, (**c**) pyran-2-ones. Below: expansion of pyran-2-one cluster (**c**). Light blue nodes represent features with VIP > 1, dark blue nodes represent features with VIP > 3. Bold numbers highlight isolated compounds.

A molecular network (MN, Figure 4) of the MES-SSW extract was constructed using LC-HRMS/MS data obtained in the positive ionization mode, including the retention time of all detected ions in order to distinguish the isobaric compounds. The MN consisted of 83 nodes representing the most abundant ions in the chemical profile, which were grouped into seven clusters. Compounds significantly discriminating the MESS-SSW extract on the OPLS-DA were searched among the nodes and mapped on the MN. Among a total of 43 nodes corresponding to compounds for which VIP > 1 (light blue nodes), seven of the ions with VIP > 3 (dark blue nodes) were present in the MN. Five compounds were not detected due to a too low relative abundance in the extract to be fragmented using the MS/MS parameters. The largest cluster (Figure 4a) corresponded to ergosterol derivatives, some of them being dereplicated by the GNPS database as ergostapentaene (*m*/*z* 375.3014), ergostahexaene (*m*/*z* 377.3163), ergostatetraen-3-one (*m*/*z* 393.3115), ergostatetraen-3-ol (*m*/*z* 395.3301) and hydroxyergostatrien-7-one (*m*/*z* 411.3253) analogues [48], among which five were overproduced in the mussel-derived medium. A more detailed list of annotated metabolites can be found in Table S2. In the second cluster (Figure 4b), the node at *m*/*z* 508.3407 was annotated as melearoride A (C_30_H_47_NO_4,_ [M+Na]^+^_,_ ∆ppm 0.83), a 13-membered macrolide previously isolated from a marine-derived *Penicillium meleagrinum* var. *viridiflavum* [50]. Four other melearoride derivatives—PF 1163E (*m*/*z* 536.3657, [M+Na]^+^_,_ C_32_H_51_NO_4_ ∆ppm -10.96) [55], PF 1163B (*m*/*z* 484.3053, [M+Na]^+^_,_ C_27_H_43_NO_5_ ∆ppm 2.91) [56,57], *N*-demethylmelearoride A (*m*/*z* 472.3422, [M+H]^+^_,_ C_29_H_45_NO_4_ ∆ppm -1.02) [51]—were dereplicated in the same cluster, for which the annotation was confirmed by a thorough manual interpretation of their MS/MS fragmentation pattern (Figure S4). Two nodes at *m*/*z* 522.3549 and 522.3563 were connected with the *m*/*z* 536.3657 node with a difference of 14.01 suggesting that they corresponded to two undescribed demethyl derivatives. Two other nodes at *m*/*z* 492.3083, 746.5283 corresponding to unknown compounds were also strongly connected within this cluster, showing a high diversity of new compounds in this rare macrolide family, which were specifically produced by the fungus on YES-SSW and MES-SSW media.

The third cluster (Figure 4c) was composed of 10 nodes related to pyran-2-one compounds, linked together by an accurate mass difference of 2.01 or 18.01. Three of them were some of the ions highlighted in the OPLS-DA with VIP > 3: *m*/*z* 215.1281 (pestalotin isomer), *m*/*z* 213.1139 (dehydropestalotin isomer) and *m*/*z* 231.1225 (2’-hydroxypestalotin isomer). Among the other nodes, four exhibited a VIP score between 1 and 3. Two of them were dereplicated as other analogs of dehydroxypestalotin and 2’-hydroxypestalotin, and the node at *m*/*z* 229.1048 was consistent with LL-P880γ. Two other nodes with the same accurate mass and similar fragmentation pattern (cosine score = 0.70 and 0.86) were linked to the latter but with a different retention time, showing that three different isomers of LL-P880*γ* were present. Some of the ions with VIP > 3 in the O-PLSDA model did not appear to be connected to this pyran-2-one cluster because they were observed as [M+Na]^+^ adducts and thus exhibited different fragmentation patterns than the corresponding protonated molecule. In particular, two other LL-P880*γ* isomers were observed as isolated nodes at *m*/*z* 251.0905. Other pyran-2-ones were manually searched among the self-loop nodes using their UV-vis absorbance spectra, on the basis of the characteristic absorbance band at 280 nm revealing a pyran-2-one ring and 238 nm for the 5,6-dihydropyran-2-one moiety. A node at *m*/*z* 197.1154 [M+H]^+^ was then annotated as 6-pentyl-4-methoxy-2*H*-pyran-2-one (λ_max_ 280 nm), a dehydroxy derivative of dehydropestalotin [12]. Manual curation of its MS/MS spectrum and comparison with those of other pyran-2-ones confirmed this annotation as it did not present an [M-18+H]^+^ fragment ion characteristic of the hydroxyl group found in all the compounds of the pyran-2-one cluster. It appeared then that most of the pyran-2-one compounds could not be clearly annotated due to the possibilities of many regio- or stereoisomerism. In this way, none of the three *m*/*z* 229.1047, 229.1048, 229.1049 nodes could be assigned as one of the four diastereoisomers of LL-P880*γ*, among which only the (1’*S*, 2’*R*) and (1’*R*, 2’*S*) analogues have been described as natural products. It was the same for the *m*/*z* 231.1237 and 231.1225 nodes, for which two of the eight possible 2’-hydroxypestalotin (LL-P880*β*) configurations have been described as natural products, these two in the 6*S* series. Thus, as some of the compounds would likely be new ones, it was decided to engage the purification and structure elucidation of all pyran-2-one derivatives found in the MES-SSW extract.

### 2.3. Isolation and Structure Elucidation of Pyran-2-ones

**Figure 5:**
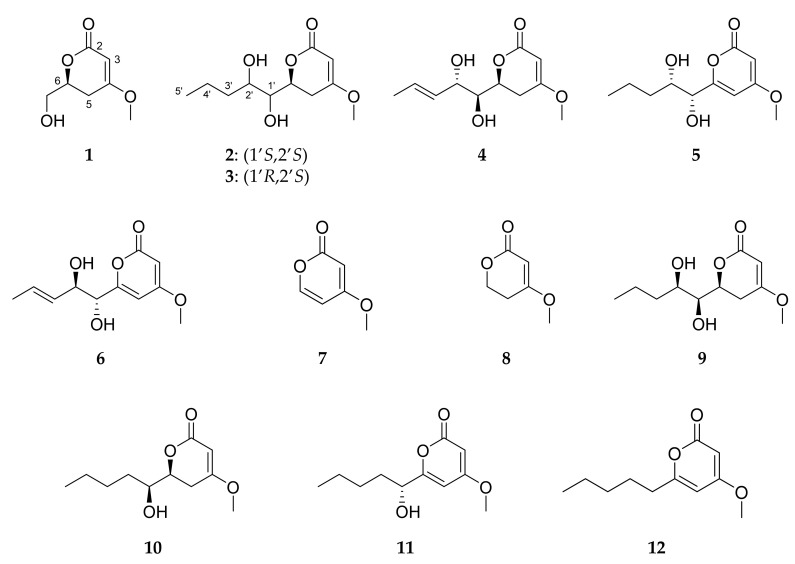
Structures of compounds **1**−**12**.

Targeted isolation of pyran-2-ones was both MS- and UV-guided, focusing on compounds detected in the cluster (III) of the MN and other compounds, showing a UV spectrum typical of a pyrone system. This led to the isolation of 12 pyran-2-ones including five new fungal compounds (Figure 5). Six of the seven known compounds were identified as (1’*R*, 2’*S*)-LL-P880γ (**5**), 5,6-dihydro-4-methoxy-2*H*-pyran-2-one (**8**) [45], (6*S*,1’*S*,2’*R*)-LL-P880*β* (**9**) [44], (6*S*,1’*S*)-pestalotin (**10**) [58,59], 1’*R*-dehydropestalotin (**11**) [60,61] and 6-pentyl-4-methoxy-2*H*-pyran-2-one (**12**) [61]. The identification of these substances was established by comparison of their physical and spectroscopic data with those reported previously and their absolute configuration was established by circular dichroism and comparison of their spectra with published data.

**Figure 6:**
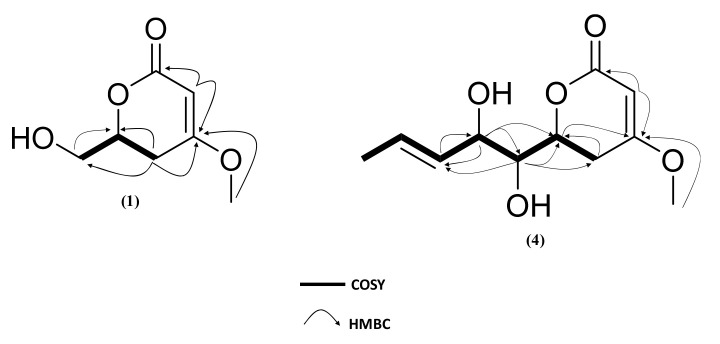
Selected HMBC and COSY correlations for compound **1**, **4**.

Compound **1** was obtained as a white amorphous powder. Its molecular formula was deduced as C_7_H_10_O_4_ with three degrees of unsaturation from the (+)-HRESIMS ions at *m*/*z* 159.0652 [M+H]^+^. The ^13^C NMR spectrum (Table 3) showed seven carbons including one oxygenated methyl group, one unsaturated methine, one oxygenated methine, one methylene, one oxygenated methylene, one unsaturated quaternary carbon and one carbonyle. The ^1^H-NMR spectrum (Table 3) revealed the presence of one methoxy group at δ_H_ 3.77 (3H, s, O-CH_3_), one methine olefinic proton at δ_H_ 5.16 (1H, dd, *J* = 1.5 Hz, H-3), one oxygenated methine at δ_H_ 4.51 (1H, m, H-6), two methylene protons at δ_H_ 2.8 (1H, ddd, *J* = 1.5, 12.5, 16.7 Hz, H-5a) and 2.28 (1H, dd, *J* = 3.8, 17.2 Hz, H-5b) and two oxygenated methylene protons at δ_H_ 3.73 (1H, dd, *J* = 4.6, 12.2 Hz, H-1’b); 3.92 (1H, m, H-1’a). Comparison of these data with those of 5,6-dihydro-(6*R*)-hydroxymethyl-4-methoxy-2*H*-pyran-2-one [62] revealed that the two compounds possessed the same planar structure. This was supported by ^1^H-^1^H COSY correlations between H-6 (δ_H_ 4.51) and H-5a/H-5b/H-1’a/H-1’b. HMBC correlations from H-3 (δ_H_ 5.16) to C-2 (δ_C_ 166.73)/C-4 (δ_C_ 172.94)/C-5 (δ_C_ 28.84), from H-5a (δ_H_ 2.8) to C-3 (δ_C_ 89.95)/C-4 (δ_C_ 174.94)/C-6 (δ_C_ 76.21)/C-1’(δ_C_ 63.74) and 4-OCH_3_ to C-3 (δ_C_ 89.95)/C-4 (δ_C_ 174.94) confirmed that the methoxy group and the hydroxymethyl were located at C-4 (δ_C_ 174.94) and C-6 (δ_C_ 76.21), respectively, of a 5,6-dihydro-2*H*-pyran-2-one moiety (Figure 6). The UV spectrum with an absorption band at λ_max_ 238 nm was also in accordance with a 5,6-dihydropyran-2-one moiety [43]. 

**Figure 7:**
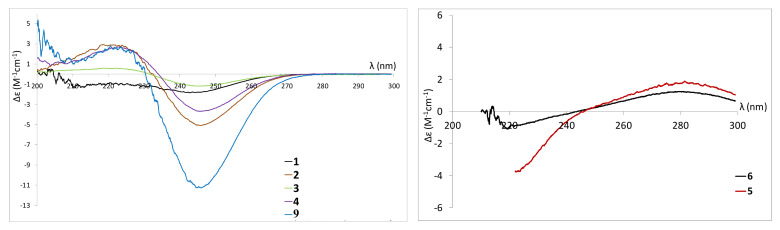
ECD spectra of compounds **1**−**6** and reference compound **9** monitored in methanol.

The absolute configuration of **1** was assigned as (6*S*) using optical rotation which was found to be of a similar magnitude to that of 5,6-dihydro-(6*R*)-hydroxymethyl-4-methoxy-2*H*-pyran-2-one [62], but opposite. The ECD spectrum confirmed this assignment as it showed a negative Cotton effect at 245 nm (Figure 7), similar to the one reported for (6*S*,1’*S*)-pestalotin [59] and compound **9** (also isolated and analysed in this study). 5,6-dihydro-6-hydroxymethyl-4-methoxy-2*H*-pyran-2-one can be found, reported as two different products in the chemical literature. It is referenced under the CAS no: 1013918-20-1 as the (6*R*) enantiomer isolated from the endophytic fungus *Pestalotiopsis sydowian* and obtained as a synthetic intermediate by Pospisil et al., 2008 [63]. However, it is also found under the reference CAS no: 1332747-99-5 as a compound isolated from the endophytic fungus *Pestalotiopsis palmarum* but without determination of its absolute configuration [62]. Therefore, compound **1** was assigned as the previously unreported 5,6-dihydro-6*S*-hydroxymethyl-4-methoxy-2*H*-pyran-2-one.

**Figure 8:**
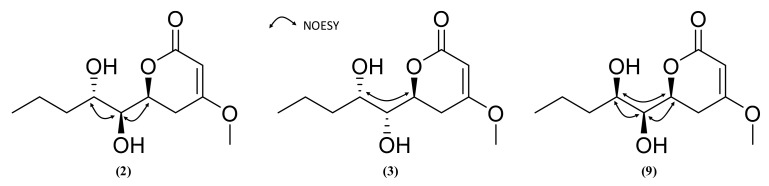
Selected NOESY correlations for compounds **2**, **3** and **9**.

Compound **2** was obtained as a white powder. Its (+)-HRESIMS data (*m*/*z* 231.1229 [M+H]^+^, 253.1050 [M+Na]^+^) indicated a molecular formula of C_11_H_18_O_5_ corresponding to a hydroxylated derivative of pestalotin such as (6*S*,1’*S*,2’*R*)-LL-P880*β* [44]. The UV spectrum in MeOH showed an absorption maximum at 238 nm, revealing a 5,6-dihydropyran-2-one moiety [43]. The ^13^C NMR spectrum displayed 11 carbons including two methyl groups, three methylenes, three methines, and two quaternary carbons. Inspection of the ^1^H NMR spectrum showed one methyl group at δ_H_ 0.95 (3H, t, *J* = 7.1 Hz, H-5’), one olefinic proton at δ_H_ 5.12 (1H, s, H-3), six methylenes at δ_H_ 1.38–1.5 (2H, m, H-4’), δ_H_ 1.5–1.63 (2H, m, H-3’), δ_H_ 3.05 (1H, dd, *J* = 13.8, 16.7 Hz, H-5a), δ_H_ 2.19 (1H, dd, *J* = 2.9, 17.0 Hz, H-5b), three oxygenated methines δ_H_ 3.37 (1H, dd, *J* = 2.0, 6.7 Hz, H-1’), δ_H_ 3.80 (1H, t, *J* = 7.1, 7.7 Hz, H-2’), δ_H_ 4.76 (1H, d, *J* = 12.8 Hz, H-6) and one methoxy group δ_H_ 3.76 (3H, s, 4-OCH_3_). The analysis of the ^1^H-^1^H COSY spectrum indicated the presence of a spin system (C-3-C-5-C-6-C-1’-C-2’-C-3’-C-4’-C-5’). Key HMBC correlations from H-3 (δ_H_ 5.12) to C-2 (δ_C_ 167.03)/C-4 (δ_C_ 173.94)/C-5 (δ_C_ 29.49)/C-6 (δ_C_ 75.41), from H-1’ (δ_H_ 3.37) to C-2 (δ_C_ 167.03)/C-5 (δ_C_ 29.49)/C-6 (δ_C_ 75.41)/C-2’ (δ_C_ 74.13), H-5a (δ_H_ 3.05) to C-2 (δ_C_ 167.03)/C-3 (δ_C_ 89.51)/C-4 (δ_C_ 173.94)/C-6 (δ_C_ 75.41)/C-1’ (δ_C_ 71.27) and from 4-OCH_3_ (δ_H_ 3.76) to C-2 (δ_C_ 167.03)/C-4 (δ_C_ 173.94)/C-5 (δ_C_ 29.49) confirmed that the methoxy group and an 1’,2’-dihydroxy five-carbon side chain were, respectively, located at C-4 and C-6 of the 5,6-dihydro-pyran-2-one. The planar structure of **2** was then established as the same as hydroxypestalotin [(6*S*,1’*S*,2’*R*)-LL-P880*β*]. The ECD spectrum of compound **2** was similar to compound **1** (Δε_240.9 nm_ = −1.92) and (6*S*,1’*S*,2’*R*)-LL-P880*β* (Δε_245.2 nm_ = −11.54) with a negative Cotton effect at 247 nm indicating its (6*S*) absolute configuration (Figure 7). The four diastereoisomers *SSR*, *SSS*, *SRS* and *SRR* have been reported so far, but only the two first have been isolated from a natural source. Their absolute configuration has been, for most of them, deduced from diagnostic coupling constants of the H-6-H-1’-H2’ system, which we investigated in the ^1^H NMR spectrum of **2**. The ^3^*J*_H-6-H-1’_ (*J* = 2.0 Hz) of **2** was different to that of (6*S*,1’*R*,2’*S*)-nodulisporipyrone D (^3^*J*_H-6-H-1’_ = 6.5 Hz) [64] and (6*R*, 1’*S*, 2’*R*)-LL-P880*β* (^3^*J*_H-6-H-1’_ = 6.7 Hz) [65] but similar to that of (6*S*,1’*S*,2’*S*)-LL-P880*β* (^3^*J*_H-6-H-1’_ = 1.1 Hz) [66], which indicated that the relationship between H-6 and H-1’ was *syn*. Moreover, the ^3^*J*_H-1’-H-2’_ (*J* = 6,7 Hz) of **2** was equivalent to that of (6*S*,1’*R*,2’*R*)-LL-P880*β* (^3^*J*_H-1’-H-2’_ = 6.1 Hz) [67] and in the same order as that of (6*R*,1’*R*,2’*R*)-LL-P880*β* (^3^*J*_H-1’-H-2’_ = 9.2 Hz) [67] and (6*S*,1’*S*,2’*S*)-LL-P880*β* (^3^*J*_H-1’-H-2’_ = 9.1 Hz) [66], which identified that the relationship between H-1’ and H-2’ was *anti*. Furthermore, the NOESY spectrum showed both an absence of correlation between H-6 and H-2’ and a strong correlation between H-1’ and H-2’. On the basis on these results, the absolute configuration of **2** was determined as 6*S*,1’*S*,2’*S* (Figure 8) and compound **2** was assigned as (6*S*,1’*S*,2’*S*)-LL-P880*β*, a natural product obtained once from a mangroved-derived *Pestalotiopsis virgatula* [66].

Compound **3** was obtained as a white powder and showed the same molecular formula C_11_H_18_O_5_ as compound **2**, with ions *m*/*z* 231.1225 [M+H]^+^, 253.1041 [M+Na]^+^ in (+)-HRESIMS. The 1D NMR (^1^H, ^13^C NMR), 2D NMR (HSQC, COSY, and HMBC), UV and ECD spectra were very similar to those of **2**, indicating the same planar structure. However, the ^3^*J*_H6-H1’_ (*J* = 3.9 Hz) was different from **2** but similar to (6*S*, 1’*R*, 2’*R*)-LL-P880*β*, (^3^*J*_H6-H1’_ = 4.0 Hz) [67], and ^3^*J*_H-1’-H-2’_ (*J* = 3.5 Hz) of **3** was different from the one of **2** but similar to (6*S*, 1’*S*, 2’*R*)-LL-P880*β* (^3^*J*_H-1’-H-2’_ = 4.0 Hz) [46]. The NOESY spectrum revealed a strong H-6/H-2’ correlation and the absence of H-1’/H-2’, and H-1’/H-6 correlation (Figure 8). Compound **3** was then assigned as (6*S*,1’*R*,2’*S*)-LL-P880*β*, a new natural product previously obtained by stereoselective synthesis [68].

Compound **4** was isolated as a white amorphous powder. Its (+)-HRESIMS spectrum displayed ions at *m*/*z* 229.1068 [M+H]^+^, 251.0884 [M+Na]^+^, and 211.0964 [M−H_2_O+H]^+^, with the formula C_11_H_16_O_5_ and four degrees of unsaturation. The UV spectrum in MeOH showed an absorption maximum at 238 nm, revealing a 5,6-dihydro-pyran-2-one moiety [43]. The ^1^H-NMR and ^13^C-NMR spectra have demonstrated that **4** was closely related with **2** and **3** except for the difference in the chemical shift of H-2’ (δ_H_ 4.32)/C-2’ (δ_H_ 72.62), H-3’(δ_H_ 5.53)/C-3’ (δ_C_ 129.24), H-4’(δ_H_ 5.88)/C-4’(δ_C_ 130.60) and H-5’(δ_H_ 1.74)/C-5’(δ_C_ 17.88). The differences in molecular formula and NMR data indicated that **4** was a reduced homologue of **2**, **3** and **9**. The HMBC correlation from H-4’ (δ_H_ 5.88) to C-2’ (δ_C_ 72.62)/C-5’ (δ_C_ 17.88) and from H-2’ (δ_H_ 4.32) to C-1’ (δ_C_ 74.79)/C-3’ (δ_C_ 129.24)/C-4’ (δ_C_ 130.60)/C-6 (δ_C_ 75.94) indicated that the double bond was adjacent to a methyl group and occurred at C-3’ and C-4’. The geometry of C-3’ (δ_C_ 129.24) was deduced to be *E* by the NOESY correlation between H-2’ and H-4’, the ^13^C-NMR deshielded chemical shift of the methyl group and the coupling constant ^3^*J*_H3’-H4’_ = 15.7 Hz. The planar structure of **4** was established, which was confirmed by the exhaustive analysis of the 2D-NMR data (HSQC, ^1^H-^1^H COSY, and HMBC) (Figure 6). The ECD spectrum of compound **4** was found to be similar to those of **1**–**3** (Figure 7). NOESY correlations between H-6 and H-1’ and between H-1’ and H-2’ together with diagnostic coupling constants ^3^*J*_H6-H1’_ = 2.0 Hz (similar to that of (6*S*,1’*S*,2’*S*)-LL-P880*β* [66] and ^3^*J*_H1’-H2’_ = 6.1 Hz (equivalent to that of **2**), permitted the assignment of the structure and absolute configuration for **4** as the new compound 5,6-dihydro-4-methoxy-6*S*-(1’*S*,2’*S*-dihydroxy pent-3’(*E)*-enyl)-2*H*-pyran-2-one, unprecedented LL-P880*β* analogue with an unsaturated side chain.

Compound **5** was obtained as a white amorphous powder. The (+)-HRESIMS analysis returned a molecular formula of C_11_H_16_O_5_ with four degrees of unsaturation from ions *m*/*z* 229.1069 [M+H]^+^, 251.0896 [M+Na]^+^, and 211.0969 [M−H_2_O+H]^+^. The UV spectrum of compound **5** in MeOH showed an absorption maximum at 280 nm characteristic of an unsaturated pyran-2-one moiety [42]. The ^1^H-NMR spectrum showed the presence of one methoxy group at δ_H_ 3.83 (3H, s, 4-OCH_3_), a pair of olefinic hydrogens at δ_H_ 5.46 (1H, d, *J* = 2.0 Hz, H-3) and 6.16 (1H, d, *J* = 2.0 Hz, H-5), a methyl at δ_H_ 0.93 (3H, 7.1, H-5’), four methylenes at δ_H_ 1.43 (2H, m, H-3’), δ_H_ 1.54 (1H, m, H-4’) and δ_H_ 1.35 (1H, m, H-4’), and two oxygenated sp^3^ methines at δ_H_ 4.44 (1H, d, *J* = 4.0 Hz, H-1’) and 4.02 (1H, m, H-2’). These data suggested that compound **5** was the reduced product of **3** in the lactone ring, which was confirmed by comparison of the ^13^C, COSY, HSQC and HMBC spectra with those of LL-P880γ [44,46,69]. The coupling constant ^3^*J*_H1’-H2’_ = 4.0 Hz and a correlation observed between H-1’ and H-2’ in the NOESY spectrum experiment of **5** indicated that they were *syn* and that the relative configuration of **5** was 1’R*,2’S* or 1’S*,2’R*. The optical rotation of compound **5**, with [α]${}_{D}^{20}$ + 63 (c 1.0, MeOH), had the same sign than (1’*R*,2’*S*)-LL-P880γ and (1’*R*,2’*R*)-LL-P880γ and the ECD spectrum of compound **5** (Δε_281.9nm_ = +2.042) exhibited a positive Cotton effect at 282 nm already described for 6-(1*R*, 2’*R*-dihydroxyheptyl)-4-methoxy-2*H*-pyran-2-one and 6-(1*R*, 2’*S*-dihydroxyheptyl)-4-methoxy-2*H*-pyran-2-one [64]. These observations allowed the assignment of the structure and absolute configuration of **5** as the new natural product (1’*R*, 2’*S*)-LL-P880γ, previously described as a synthetic compound [69] and reported from *Xylaria feejeenisis* but without stereochemistry data [70].

**Figure 9:**
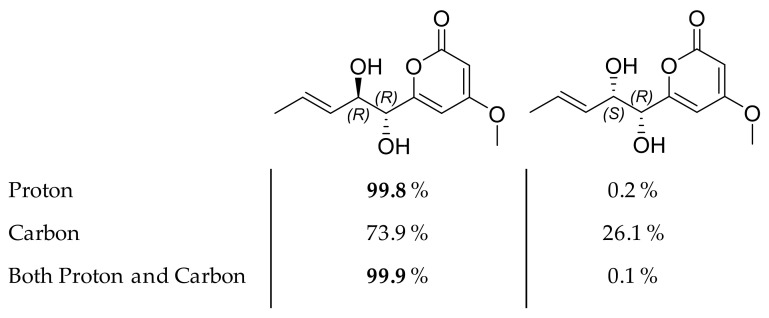
Chemical structures of two possible stereoisomers of compound **6** with their respective DP4 probabilities.

Compound **6** was isolated as a white amorphous powder. The (+)-HRESIMS spectrum of **6** displayed ions at *m*/*z* 227.0921 [M+H]^+^, 249.0743 [M+Na]^+^, and 209.0822 [M−H_2_O+H]^+^, corresponding to the formula C_11_H_14_O_5_ with five degrees of unsaturation. This difference in molecular formula compared to **5** (-2H) and its UV spectrum presenting the same λ_max_ = 280 nm indicated that **6** was a reduction product of **5** on the side chain of the pyran-2-one moiety. The ^1^H and ^13^C-NMR spectra (Table 3) were also very similar to those of compound **5**, except for the absence of the signals corresponding to the H-3’ and H-4’ methylenes. Instead, two olefinic methines were observed at δ_H_ 5.5 (1H, ddd, *J* = 2.01, 1.3, 15.5 Hz, H-3’) and δ_H_ 5.83 (1H, dq, *J* = 6.7, 15.5 Hz, H-4’) with an HMBC correlation from H-5’ to C-3’ and C-4’ suggesting that the double bond was adjacent to the methyl group. A mutual transform coupling (*J* = 15.5 Hz) indicated an *E* configuration of the double bond. Based on the above analysis, the planar structure of **6** was established. The absolute configuration at C-1’ was deduced to be *R* from the observation that the ECD spectrum of compound **6** was similar to that of **5** (Figure 7). According to these data, the relative configuration of compound 6 could be proposed as 1’R*, 2’R* or 1’R*, 2’S*. The ^1^H and ^13^C NMR shifts of these two possible stereoisomers were computed using density functional theory. After correlating the experimental and simulated data through the DP4 probability method [71,72], the relative configuration of 1’*R*, 2’*R* was identified as the most likely candidate in high probability, with 99.9% (both ^13^C and ^1^H NMR), 99.8% (only ^1^H NMR), and 73.9% (only ^13^C NMR) probabilities values according to DP4 calculations (Figure 9). Finally, the structure of compound **6** was established as 4-methoxy-6-(1’*R*, 2’*R*-dihydroxy pent-3’(*E)*-enyl)-2*H*-pyran-2-one.

Compound **7** had the molecular formula C_6_H_6_O_3_ as indicated by the (+)-HRESIMS *m*/*z* 127.0416 [M+H]^+^. As for compounds **5** and **6**, the UV spectrum of compound **7** was consistent with the presence of a pyran-2-one moiety [42]. The ^1^H, ^13^C-NMR analysis (Table 3) allowed a final identification of compound **7** as 4-methoxy-2*H*-pyran-2-one. Compound **7** was originally reported as a synthetic intermediate during the synthesis of patulin [73] and, surprisingly, recently as a constituent of the roots of *Glycyrrhiza uralensis* [74]. It is described here for the first time as a fungal natural product.

### 2.4. Biological Evaluation of Isolated Compounds

Due to the limited amount, only some of the isolated products were tested for cytotoxic, antibacterial and antileishmanial activities. Compounds **7**, **10**–**12** did not exhibit antiproliferative activity against the epithelial cancer cell line KB when assayed at a concentration of 50 µg/mL. Compounds **2**, **3**, **7**, **10**–**12** were screened for their antibacterial activities against *Escherichia coli*, *Pseudomonas aeruginosa*, *Staphylococcus aureus* and *Enterococcus faecalis.* None of them showed antibacterial activity at a concentration of 100 µg/mL. Antileishmanial activity of **10** was evaluated through determination of 50% efficient concentration against *Leishmania infantum* amastigotes. The results of this assay showed no detectable activity at concentrations ≤ 50 µg/mL.

### 2.5. PTP1B Docking

**Figure 10:**
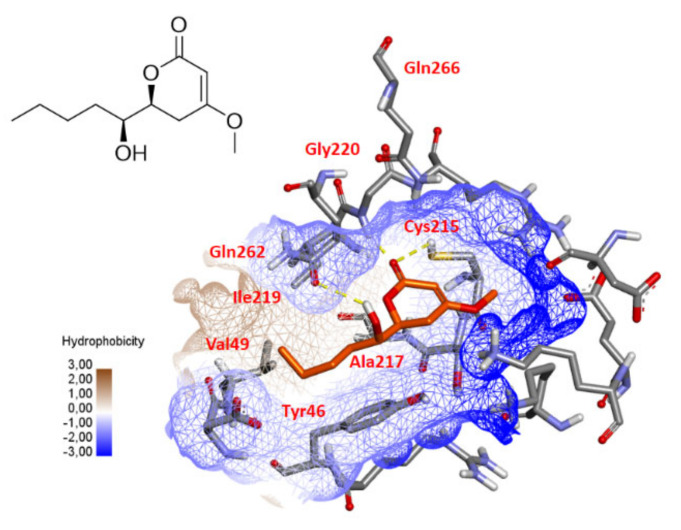
Possible docking solution of compound **10** (goldscore.fitness: 32.2589) in the active site of PTP1B (1NWL.pdb). Hydrogen bonds are indicated as yellow lines.

Recently, 3,4,6-trisubstituted pyran-2-one derivatives, chrysopyrones A and B have been described as potent inhibitors of protein tyrosine phosphatase 1B (PTP1B) [75]. To investigate a possible interaction between compound **10** and PTP1B, a molecular docking was performed using Gold software [76]. As shown in Figure 10, a docking result revealed that the carbonyl group of the pyran-2-one core could form hydrogen bonds with the –NH of Gly220 and –SH of Cys215, which is essential for catalysis [77,78], while the hydroxyl group could form a hydrogen bond with the c*arbonyl* oxygen atom of the *Gln262 amide* side chain, another important residue in the second catalytic step [79]. The hydrophobic side of the molecule also makes van der Waals contacts with the side chains of Tyr46, Val49, Ala217 and Ile219. All these features are consistent with a possible inhibition of PTP1B that will have to be confirmed by experimental assays.

## 3. Discussion

Stress adaptation and metabolic response are critical mechanisms for the survival of microbes in a dynamic environment and for the colonization of host niches. Fungi can sense and then transduce external changes in osmotic pressure mainly through cellular signalling pathways such as the HOG pathway and then activated transcriptional activators will modulate the pattern of gene expression, leading to the production of osmoprotectant compounds such as glycerol [80]. Some studies in the literature have shown that the response to osmotic stress by fungi is in some cases linked to the biosynthesis of natural products [80,81,82,83]. For investigating new bioactive compounds in marine fungi, several strategies have been used to induce the expression of silent natural products including the use of abiotic stresses such as high NaCl concentrations [81,82]. In *Aspergillus aculeatus*, osmotic and saline stress exerted by glycerol and NaCl, respectively, can modulate the production of natural products [82]. Similarly, the expression of polyketides was upregulated and de novo observed following the use of a combination of SAHA and osmotic stress in *A. cruciatus* [80]. Here, we observed that it was the combination of the use of seawater and the mussel-derived medium that induced the overexpression of some specialized compounds, i.e., culture conditions that can be considered as the closest to the original environment of the *P. restrictum* strain. This illustrates that there may be a specific metabolome of the living conditions within holobionts such as bivalves, which can be tentatively reconstituted in vitro. 

The use of both metabolomics and feature-based molecular networking (FBMN) allowed to highlight compounds and to cluster them into chemical series. These tools are of high value for enhancing the dereplication of known compounds and can allow annotation propagation for the discovery of new analogs in a chemical series [84,85], as is shown here for melearorides. However, for small molecules and trace compounds such as the pyran-2-ones described here, fragmentation information is usually poor and they can escape MS/MS fragmentation because they are below the method threshold, particularly in complex mixtures such as fungal culture extracts. Furthermore, some compounds can be undetected and unrepresented in the MN, as in the case of isomers, which can lead to similar fragmentation patterns. The observation of multiple isobaric compounds in an MN highlights these limits of MS-based dereplication, as shown in this study for LL-P880*β* stereoisomers. Then, adding further physico-chemical properties such as retention time and UV-Vis spectrum in the MN is mandatory to optimize the resolution of the detection of metabolites diversity, but it does not enhance the strength of annotations. In this work, using a combination of MS-guided and UV-guided fractionation was the only way to fully characterize the targeted compounds, which successfully yielded to the isolation of, among others, three isomers of LL-P880*β*, including one new natural product. 

The 12 isolated pyran-2-ones were found to belong to two sub-series according to their degree of insaturation of the lactone ring. These kinds of compounds are commonly found among fungi, but only the 6*S* stereoisomers have been described as natural products, with the exception of 5,6-dihydro-(6*R*)-hydroxymethyl-4-methoxy-2*H*-pyran-2-one, the smallest C6-substituted analogue, which was previously isolated from *Pestalotiopsis sydowiana* [62]. In our study, all the compounds belonged to the 6*S* series, including the new **1**, which completes the chemical family, showing that the biosynthetic route induced in these environment-related conditions was unique and stereospecific. 

If monocyclic pyran-2-ones attracted much attention due to their wide distribution and chemodiversity in microbes, their biological evaluations always reported that they exert no or minor cytotoxicity nor antibacterial or antiparasitic activity, as we observed here [86,87]. Only some members were found to inhibit seed germination or plant growth, to inhibit 20S proteasome [62], to enhance the growth-stimulating action of gibberellic acid [59], or to be selectively active against some yeasts or filamentous fungi [88,89]. Their role in the chemical interaction between a fungus and its host remains then unknown, but hypotheses can be proposed. Due to their structural similarity with homoserine lactones, some synthetic and natural pyran-2-ones have been shown to act as quorum sensing inhibitors. Usually, it is considered that the longer the alkyl chain, the higher the QS inhibition activity [90]. However, it has also been discovered that pyran-2-ones, including short-branched ones, can act as signalling molecules in some bacteria such as *Pseudomonas luminescens* [91]. It can then be envisaged that small pyran-2-ones can be engaged in signal recognition processes and in chemical interactions between fungi and hosted bacteria inside the bivalve.

Pyran-2-ones were reported to possess potent inhibitory activities against protein tyrosine phosphatases 1B (PTP1B), as shown for two 3,4,6-trisubstituted pyran-2-one derivatives, chrysopyrones A and B, isolated from a marine *Penicillium chrysogenum* [80]. Protein tyrosine phosphatases (PTPs) are of importance in the regulation of a myriad of cellular signal transduction systems. They are involved in meiosis, metabolism, cell-substrate and cell-cell adhesion, and sporulation in yeasts. They also play a role in responses to environmental changes and stresses [92]. PTP1B is the major negative regulator of insulin signalling in mammals by catalysing the phosphorylation of the insulin receptor or insulin receptor substrate [93]. Furthermore, small pyran-2-ones seem to be promising candidates for developing new PTP1B inhibitors [94,95,96]. The results we obtained from the in silico docking of 6*S*, 1’*S*-pestalotin (**10)** showed that it can bind to important residues in the catalytic site of PTP1B, including some amino acids within the PTP-loop that are highly conserved in all PTPs. A whole-genome sequencing project of the cosmopolitan Mediterranean mussel (*Mytilus galoprovincalis*) has been conducted recently, showing that some genes with tyrosine phosphatase-related functions were present in mussels [97]. Therefore, it can be envisaged that an inhibition of PTPs is one of the mechanisms involved in the interaction of the fungus with its host. Further biological studies should be conducted to determine whether pyran-2-ones play these or other roles within molluscs.

## 4. Materials and Methods

### 4.1. Fungal Material

*Penicillium restrictum* MMS417 was isolated from a sample of blue mussel *Mytilus edulis* collected in January 1997 at Port Giraud on the Loire estuary in France. The strain is stored at the laboratory Mer-Molécule-Santé (MMS) fungal culture collection, University of Nantes, France. The strain has been identified according to macroscopic and microscopic observations, and sequencing of the internal transcribed spacer (ITS) and *β*-tubulin regions of the rDNA and nucleotide BLAST search (GenBank accession number for MMS417: KU720404, KU720398).

### 4.2. Culture Media Preparation

Fungal cultures were performed in Petri dishes (10 cm diameter) containing 15 mL of solid agar-based medium in 14 different media. Two osmotic conditions were prepared based on the presence or absence of artificial seawater (36 g/L of salinity). The media compositions were as follows: DCA (dextrose 40 g/L, enzymatic digest of casein 10 g/L, agar 15 g/L, Difco); MEA (malt extract 20 g/L, peptone 1 g/L, glucose 20 g/L, agar 20 g/L); PDA (potato extract 4 g/L, dextrose 20 g/L, ZnSO_4_·7H_2_O 0.01 g/L, CuSO_4_·5H_2_O 0.005 g/L, agar 15 g/L); YES (yeast extract 20 g/L, sucrose 150 g/L, agar 20 g/L); MES (mussel extract 20 g/L, sucrose 150 g/L, agar 20 g/L); CYA (Czapek concentrate 10 mL, yeast extract 5 g/L, K_2_HPO_4_ 1 g/L, sucrose 30 g/L, agar 15 g/L); Czapek concentrate (NaNO_3_ 3 g/L, KCl 0.5 g/L, MgSO_4_·7H_2_O 0.5 g/L, FeSO_4_·7H_2_O 0.01g/L, ZnSO_4_·7H_2_O 0.01 g/L, CuSO_4_·5H_2_O 0.05 g/L); KMS (MgSO_4_.7H_2_O 2.4 g/L, NH_4_NO_3_ 2.4 g/L, Tris (tampon) 1.21 g/L, Agar 20 g/L). Details for the preparation of mussel extract are presented in a previous study [16].

### 4.3. Fermentation and Extraction for OSMAC Approach

Fungal cultures were carried out in triplicate for each medium from culture stocks of the strain stored at −20 °C and transplanted on Petri dishes of DCA medium 10 days before inoculation. Culture inoculations were performed by taking fragments of mycelium and conidia using a sterile Pasteur pipette and deposited at three points on the agar. Cultures were incubated at 27 °C for 10 days under natural light. Plugs (6 mm diameter) were taken in three distinct places of cultures (at the point of central impact, on the outskirts of the colony, in the periphery but contacting an adjacent colony). The three plugs were gathered together and extracted twice with 1.5 mL of CH_2_Cl_2_/EtOAc 1:1 (*v*/*v*). Fungal mycelium and agar were extracted together to obtain both intra- and extracellular metabolites. Mixtures were sonicated for 30 min and filtered on 0.45 *µ*m regenerated cellulose filters (Sartorius). Organic phases were combined and evaporated to dryness, leading to an organic extract. Non-inoculated agar media were also extracted following the same protocol and used as controls (blank samples).

### 4.4. HPLC-MS Analyses

HPLC-(+)-HRESI-MS and HPLC-(+)-HRESI-MS/MS analyses were performed on a UFLC-MS (IT-TOF) Shimadzu instrument (combining ion trap and time of flight analyzers), using a Kinetex C18 column (2.6 µm, 2.1 × 100 mm, Phenomenex) and following previously described conditions [41,98]. MS/MS fragmentations were obtained by applying the following parameters: energy 50%, collision gas 50%, *q* (frequency) 0.251 (45.0 Hz). Precursor ion selection was performed in the range *m*/*z* 150–1000. Daughter ions were measured in the range *m*/*z* 100–1000 with 30 ms accumulation, an execution trigger set at 1 × 10^6^, and an excluding dynamic range of 3 s. Samples (5 µL) were injected at the concentration of 1 mg/mL in MeOH. MeOH blanks were injected randomly during the analysis sequences. A mixture of all extracts at the same concentration was also prepared as a quality control (QC) and regularly injected throughout the sequences. QC samples were analysed intermittently for the duration of the analytical study to assess the variance observed in the data throughout the sample preparation, data acquisition, and data pre-processing steps.

### 4.5. Data Processing

The HPLC-(+)-HRESI-MS chromatogram raw data files were converted to *.netCDF files by using LCMS solution (version 3.60—Shimadzu). The *.netCDF files were subjected to MZmine 2 [99] for automatic peak picking. The mass detection was performed using a centroid algorithm with a noise level of 1.4^E^4. The chromatogram building was established using the ADAP algorithm with minimum of group size in the number of scans of 4, a group intensity threshold of 1^E^5, a minimum of the highest intensity of 2.1^E^4 and *m*/*z* tolerance of 100 ppm. The peak deconvolution was performed using the ADAP algorithm with median *m*/*z* center calculation, a signal-to-noise threshold of 5, minimum feature height of 1, coefficient/area threshold of 100, peak duration range from 0.08 to 23.69 min and retention time wavelet range from 0.01 to 0.2 min. The chromatograms were deisotoped with an *m*/*z* tolerance of 0.003 *m*/*z*, retention time tolerance of 1 min, maximum charge of 2, and the lowest *m*/*z* was chosen as representative. The duplicate peaks were filtered with an *m*/*z* tolerance of 10 ppm and retention time tolerance of 0.05 min. The peak list was aligned using the Ransac aligner algorithm with an *m*/*z* tolerance of 100 ppm, retention time tolerance of 1 min, retention time tolerance after correction of 1 min, Ransac interactions of 20000, and a minimum number of points of 50.0%; the value of threshold was 1, and a linear model was chosen. The peak list rows were filtered with minimum peaks in a row of 3. Then, the gap-filling step was performed with a peak finder (multithreaded) algorithm, intensity tolerance of 30%, *m*/*z* tolerance of 5 ppm, and a retention time tolerance of 0.5 min. All duplicate peaks were filtered by setting the *m*/*z* tolerance of 5 ppm and retention time tolerance of 0.05 min. The results were exported as a *.csv file containing all peaks observed and referenced by their mass-to-charge ratio (*m*/*z*) and retention times (t_R_) together with their respective peak areas in each sample. The generated matrix was cleaned by removing all peaks abundantly found in blank samples (culture media extracts) and technical blank samples (MeOH injections). The final data matrix corresponded to a total of 882 features (*m*/*z*-t_R_) and their respective areas in the 56 samples investigated (14 groups of four biological replicates).

### 4.6. Statistical Analysis

The data set containing 882 features was first transformed to reflect full compound production by multiplying each peak area by the extract amount of the corresponding samples. Then, the data was normalized using Pareto scaling and submitted to principal component analysis (PCA) and orthogonal projection to latent structures discriminates analysis (OPLS-DA) using SIMCA13 (UMETRICS). Variable Influence in the Projection (VIP) scores were provided by SIMCA13 software. They account for the significance of variables in the maximised groups’ separation in the OPLS-DA model [100]. VIPs > 1 were considered as significantly contributing to the separation.

### 4.7. Molecular Networking

The HPLC-(+)-HRESI-MS/MS raw data files were converted to *.netCDF files using LCMS solution (version 3.60—Shimadzu). The LC-MS/MS data was subjected to automatic peak picking using MZmine 2 [99] with the above-mentioned parameters. The peak list was exported as a *.mgf file for GNPS. The *.mgf file was subjected to the online workflow of the Global Natural Products Social molecular networking platform (https://gnps.ucsd.edu, accessed on 29 June 2021) [101]. The molecular network was generated using the following settings: precursor ion mass tolerance of 2 Da and fragment ion mass tolerance of 0.3 Da, minimum pairs cosine of 0.1, network topK of 10, minimum matched fragment ions of 2, minimum cluster size of 1; the run MS cluster and filter precursor window tolls were turned off. It can be visualised via the following link: https://gnps.ucsd.edu/ProteoSAFe/status.jsp?task=58f66fb7b73745ce95ea632e948dc1a9, accessed on 29 June 2021. The molecular network was finally visualized using Cytoscape 3.7.1 [102].

### 4.8. Annotation of MS Features

Compound annotation based on MS/MS data was first performed using the GNPS platform [84,101]. Then, unannotated features were further identified using an alternative strategy [103]. The feature molecular formula was determined using SIRIUS3 [104] and used to mine for possible annotation in various natural product databases, such as the Dictionary of Natural Products (DNP, CRC Press), Antibase (Wiley), and KNApSAcK [105]. Fungal-related annotations were further confirmed by an exhaustive literature search for taxonomic consistency.

### 4.9. Isolation and Identification of Specialized Metabolites

Large-scale cultivation on the MES-SSW medium was performed in 54 Erlenmeyer flasks (250 mL), containing 50 mL of media. Extraction with CH_2_Cl_2_/EtOAc 1:1 (*v*/*v*) resulted in 11.5 g of extract. The extract was fractionated using silica gel vacuum liquid chromatography (VLC) eluting with mixtures of solvents with increasing polarity: hexane/EtOAc (from 100:0 to 30:70 (*v*/*v*)) to yield six fractions, then CH_2_Cl_2_/MeOH (from 100:0 to 0:100 (*v*/*v*)) to yield five fractions. Fraction F8 (250.2 mg) was subjected to silica gel flash chromatography (column Reveleris, Silica 40 µm, 4 g) eluting with mixtures of CH_2_Cl_2_/EtOAc (from 100:0 to 60:40 (*v*/*v*)), and then CH_2_Cl_2_/MeOH (50:50 (*v*/*v*)) at a flow rate of 15 mL/min to afford 13 subfractions. Fraction F8-6 (84 mg) was subjected to silica HPLC (column Luna^®^ 5 µm, Silica, 100 Å, 250 × 10 mm), with a mobile phase consisting in MeOH/H_2_O (from 3:97 to 25:75 (*v*/*v*)) at a flow rate of 4 mL/min, to yield 12 subfractions. F8-6-4 (15 mg) was fractionated by RP-pentafluorophenyl HPLC (column Kinetex^®^ 5 µm F5, 100 Å, 250 × 4.6 mm), with the mobile phase MeOH/H_2_O (20:80 (*v*/*v*)) at a flow rate of 1 mL/min, to obtain **1** (0.5 mg), **2** (3.2 mg) and **3** (5 mg). Fraction F8-6-7 (5.5 mg) was purified by RP-pentafluorophenyl HPLC (column Kinetex^®^ 5 µm F5, 100 Å, 250 × 4.6 mm), with the mobile phase MeOH/H_2_O (25:75 (*v*/*v*)) at a flow rate of 0.5 mL/min, to afford **5** (1.4 mg) and **6** (0.7 mg). F8-7 (8.7 mg) was subjected to silica HPLC (column Luna^®^ 5 µm, Silica, 100 Å, 250 × 10 mm), with the mobile phase MeOH/H_2_O (from 3:97 to 25:75 (*v*/*v*)) at a flow rate of 4 mL/min, giving **4** (0.7 mg). Fraction F6 (253.8 mg) was subjected to another silica gel flash chromatography (column Macherey-Nagel, Chromabond^®^ flash RS25-SiOH) eluting with mixtures of CH_2_Cl_2_/MeOH (from 100:0 to 0:100 (*v*/*v*)), at a flow rate of 20 mL/min to afford 12 subfractions. F6-3 (25.2 mg) was subjected to another silica gel flash chromatography eluting with mixtures of Hexane/EtOAc (from 100:0 to 0:100 (*v*/*v*)) followed by 100% MeOH to yield six fractions. F6-3-3 (2 mg) was purified by isocratic silica HPLC (column Interchim^®^ 5 µm, Silica, 250 × 4.6 mm), with a mobile phase of CH_2_Cl_2_/EtOAc 98:2 (*v*/*v*) at a flow rate of 1 mL/min, to afford **7** (0.9 mg).

*5,6-dihydro-6S-hydroxymethyl-4-methoxy-2H-pyran-2-one* (**1**) White amorphous powder; [α]${}_{D}^{20}$ −30 (c 0.2, MeOH); UV (MeOH) λ_max_ 238 nm; CD (5.06 × 10^−3^ M, MeOH) Δε_240.9_ = −1.92; ^1^H and ^13^C NMR data, Table 3; (+)-HRESIMS *m*/*z* 159.0652 [M+H]^+^ (calculated for C_7_H_10_O_4_, 158.058 Da).

*6S-(1’S,2’S-dihydropentyl)-4-methoxy-5,6-dihydro-2H-pyran-2-one* (**2**): White amorphous powder; [α]${}_{D}^{20}$.−61.71 (c 0.58, MeOH); UV (MeOH) λ_max_ 238 nm; CD (1.74 × 10^−3^ M, MeOH) Δε_221.4_ = +3.18, Δε_247_ = −5.2; ^1^H and ^13^C NMR data, Table 3; (+)-HRESIMS *m*/*z* 231.1229 [M+H]^+^ (calculated for C_11_H_18_O_5_, 230.1156 Da).

*6S-(1’R,2’S-dihydropentyl)-4-methoxy-5,6-dihydro-2H-pyran-2-one* (CAS 58846-08-5) (**3**): White amorphous powder; [α]${}_{D}^{20}$ − 38.29 (c 0.067, MeOH); UV (MeOH) λ_max_ 238 nm; CD (4.78 × 10^−3^ M, MeOH) Δε_222.8_+ 0.66, Δε_245.6_ = −1.2; ^1^H and ^13^C NMR data, Table 3; (+)-HRESIMS *m*/*z* 231.1225 [M+H]^+^ (calculated for C_11_H_18_O_5_, 230.1152 Da).

*5,6-dihydro-4-methoxy-6S-(1’S,2’S-dihydroxypent-3(E)-enyl)-2H-pyran-2-one* (**4**). White amorphous powder; UV (MeOH) λ_max_ 239 nm; CD (8.77 × 10^−4^ M, MeOH) Δε_223.6_ = +3.01, Δε_246.6_ = −3.75; ^1^H and ^13^C NMR data, Table 3; (+)-HRESIMS *m*/*z* 229.1068 [M+H]^+^ (calculated for C_11_H_16_O_5_, 228.1 Da).

*(1’R, 2’S)-LL-P880γ* (CAS 176780-71-5) (**5**): White amorphous powder; [α]${}_{D}^{20}$ + 63 (c 1.0, MeOH). UV (MeOH) λ_max_ 280 nm; CD (1.2 × 10^−3^ M, MeOH) Δε_281.9_ = +2.042; ^1^H and ^13^C NMR data, Table 3; (+)-HRESIMS *m*/*z* 229.1069 [M+H]^+^ (calculated for C_11_H_16_O_5_, 228.1 Da).

*4-methoxy-6-(1’R,2’R-dihydroxypent-3’(E)-enyl)-2H-pyran-2-one* (**6**). White amorphous powder; [α] +55.2 (c 0.42, MeOH); CD (6.2 × 10^−3^ M, MeOH) Δε_275.7_ = +1.31; UV (MeOH) λ_max_ 280 nm; ^1^H and ^13^C NMR data, Table 3; HRESIMS *m*/*z* 227.0921 [M+H]^+^ (calculated for C_11_H_14_O_5_, 226.0848 Da).

*4-methoxy-2H-pyran-2-one* (**7**) White amorphous powder; UV (MeOH) λ_max_ 198, 274 nm; ^1^H and ^13^C NMR data, Table 3; (+)-HRESIMS *m*/*z* 127.0411 [M+H]^+^ (calculated for C_6_H_6_O_3_, 126.034 Da).

### 4.10. Computational Details

All density functional theory (DFT) calculations have been performed using Gaussian 16W. A conformational analysis on both like and unlike diastereoisomers gave rise to 16 and 37 conformers, respectively. After geometry optimization at the 6-31G(d) level, NMR prediction was realized at the mpw1pw91/6-311+g(2d,p) level on each conformer. Comparison between experiment and theoretical NMR chemical shifts was achieved by calculation of the DP4 probability [71]. 

### 4.11. Cytotoxicity Assays

Cytotoxicity assays were carried out using the MTT assay and following the procedure previously described [41]. KB cell line was purchased from the European Collection of Animal Cell Cultures (ECACC).

### 4.12. Antimicrobial Assay

Compounds were tested for antimicrobial activity via a paper disk diffusion method, according to the Clinical and Laboratory Standards Institute (CLSI) protocol [106]. Seed cultures of *Escherichia coli* ATCC 25922, *Pseudomonas aeruginosa* ATCC 27853, *Staphylococcus aureus* ATCC 25923 and *Enterococcus faecalis* ATCC 19433 were prepared by incubating the organism for 18 h at 37 °C. Aliquots of overnight cultures at 1 × 10^7^ CFU/mL were spread onto the surfaces of nutrient agar. Sterile filter disks (6 mm diameter), which were plotted with 10 µL of test solution (100 µg/mL in EtOH 1%), positive control (gentamicin sulfate) or vehicle only (EtOH 1%), were added to the plates. Plates were left upright for 30 min at room temperature before being placed in an incubator at 37 °C for 18 h, and then the growth inhibition zone diameter was measured. 

### 4.13. Antileishmanial Activity on Leishmania infantum Axenic Amastigotes

*Leishmania infantum* promastigotes (MHOM/MA/67/ITMAP-263, CNR Leishmania, Montpellier, France, expressing luciferase activity) were cultivated in RPMI 1640 medium supplemented with 10% foetal calf serum (FCS), 2 mM L-glutamine and antibiotics (100 U/mL penicillin and 100 μg/mL streptomycin) and harvested in logarithmic phase of growth by centrifugation at 900 g for 10 min. The supernatant was removed carefully and was replaced by the same volume of RPMI 1640 complete medium at pH 5.4 and incubated for 24 h at 24 °C. The acidified promastigotes were then incubated for 24 h at 37 °C in a ventilated flask to transform promastigotes into axenic amastigotes. The effects of the tested compounds on the growth of *L. infantum* axenic amastigotes were assessed as follows: *L. infantum* amastigotes were incubated at a density of 2 × 10^6^ parasites/mL in sterile 96-well plates with various concentrations of compounds dissolved in MeOH (final concentration less than 0.5% *v*/*v*), in duplicate. Appropriate controls, DMSO, MeOH and amphotericin were added to each set of experiments. After a 48-h incubation period at 37 °C, each plate-well was then microscopically examined to detect any precipitate formation. To estimate the luciferase activity of axenic amastigotes, 80 μL of each well were transferred to white 96-well plates, Steady Glow^®^ reagent (Promega) was added according to the manufacturer’s instructions, and plates were incubated for 2 min. The luminescence was measured in Microbeta Luminescence Counter (PerkinElmer). Efficient concentration 50% (EC_50_, mean of three independent experiences) was defined as the concentration of drug required to inhibit the metabolic activity of *L. infantum* amastigotes compared to control by 50%. 

### 4.14. Docking

Molecular modelling studies were performed using SYBYL-X 2.1.1 software (SYBYL-X, Tripos Associates, Inc. 1699 South Hanley Road, St. Louis, MO 63144, USA) running on an HP Z-Book Studio G3 workstation. The three-dimensional structure of compound 10 was built from a standard fragments library and optimized using the Tripos force field [107] including the electrostatic term calculated from Gasteiger and Hückel atomic charges. Powell’s method available in Maximin2 procedure was used for energy minimization until the gradient value was smaller than 0.001 kcal/(mol·Å). The crystal structure of protein tyrosine phosphatase 1B at a 2.4 Å resolution (PDB ID 1NWL) [108] was used as a template for docking. All water molecules were removed from the coordinates set. Flexible docking of compound 10 into the binding site was performed with GOLD software [76] using GoldScore fitness function. Finally, the selected complex was energy-minimized using Powell’s method available in Maximin2 procedure with the Tripos force field and a dielectric constant of 4.0, until the gradient value reached 0.1 kcal/mol·Å. Biovia Discovery Studio Visualizer (Dassault Systèmes BIOVIA, Discovery Studio Visualizer, v19.1.0.18287, San Diego: Dassault Systèmes, 2018) was used for graphical display.

### 4.15. Electronic Circular Dichroism Measurements

The electronic circular dichroism (ECD) spectra of small molecules were measured using a J-810 spectropolarimeter (Jasco, Japan) and a mini-quartz cell with a path length of 0.2 cm. The spectra were recorded in the range from 200 to 300 nm and averaged over three scans to increase the signal-to-noise ratio (response time, 0.125 s; data pitch 0.2 nm, scanning speed 50 nm/min). The final concentrations of compounds 1–6 and reference compound 9 were 0.2% in methanol. All the spectra were corrected by subtracting the spectrum of methanol.

## Figures and Tables

**Table 1:** Putative identification of characteristic ions highlighted in the OPLS-DA S-plot comparing media following salinity.

	VIP	*m*/*z*	R_t_ (min)	Observed Features (Ionic Species)	Molecular Formula for M	∆ppm	UV-vis Absorption (λ_max_ nm)	Putative Annotation
**SSW**	4.05	231.1225	6.52	[M+H]^+^	C_11_H_18_O_5_	−3.24	238	2’-hydroxy pestalotin isomer
		253.1050		[M+Na]^+^				
	3.46	411.3253	23.41	[M+H]^+^	C_28_H_42_O_2_	−2.44	n.d.	3*β*-hydroxyergosta-8,14,24(28)-trien-7-one
	3.43	472.3422	22.27	[M+H]^+^	C_29_H_45_NO_4_	−1.02	n.d.	*N*-demethylmelearoride A
		494.3197		[M+Na]^+^				
	3.15	486.3583	23.86	[M+H]^+^	C_30_H_47_NO_4_	−0.07	n.d.	Melearoride A
		508.3407		[M+Na]^+^				
	2.8	443.3150	23.87	[M+H]^+^	C_28_H_42_O_4_	−2.56	n.d.	Paxisterol
	2.5	215.1281	9.08	[M+H]^+^	C_11_H_18_O_4_	−1.09	237	Pestalotin isomer
	2.42	412.3335	23.28	[M+H]^+^	C_26_H_41_N_3_O	−1.53	n.d.	-
	2.34	427.3221	23.76	[M+H]^+^	C_28_H_42_O_3_	2.06	n.d.	-
	2.12	467.3157	19.62	[M+Na]^+^	C_28_H_44_O_4_	4.22	n.d.	Antibiotic Mer-NF 8054A
**DW**	6.33	213.1139	9.82	[M+H]^+^	C_11_H_16_O_4_	−2.78	279	Dehydropestalotin isomer
		235.0972		[M+Na]^+^				
	4.08	213.1132	10.03	[M+H]^+^	C_11_H_16_O_4_	2.42	237	Pyran-2-one derivative
	3.89	307.1577	14.92	[M+H]^+^	C_17_H_22_O_5_	10.26	n.d.	Antibiotic TAN 1446A
	3.88	213.1132	9.09	[M+H]^+^	C_11_H_16_O_4_	−3.18	288	Dehydropestalotin isomer
	3.82	251.0899	11.56	[M+Na]^+^	C_11_H_16_O_5_	1.42	275	LL-P880*γ* isomer
		267.0638		[M+K]^+^				
	3.72	129.0549	2.18	[M+H]^+^	C_6_H_8_O_3_	−2.09	237	5,6-dihydro-4-methoxy-2*H*-pyran-2-one
	3.24	431.2787	22.98	[M+Na]^+^	C_24_H_40_O_5_	3.14	n.d.	Aspergillus acid A
	2.97	515.2648	24.36	[M+H]^+^	C_29_H_38_O_8_	0.60	n.d.	Citreohybridone B
	2.9	147.0654	2.13	[M+H]^+^	C_6_H_10_O_4_	−2.27	237	5-hydroxy-3-methoxy-2-pentenoic acid
	2.79	379.3358	20.40	[M+H]^+^	C_28_H_42_	−1.78	n.d.	-
	2.58	185.0845	5.88	[M+H]^+^	C_12_H_11_NO	2.36	n.d.	-
	2.22	544.3654	18.59	[M+H]^+^	C_32_H_49_NO_6_	2.91	n.d.	Pestalotiopin B
	2.11	520.3413	16.45	[M+ Na]^+^	C_31_H_47_NO_4_	1.96	n.d.	-

n.d.: UV maxima not determined; -: not hit from fungal natural product databases.

**Table 2:** Identification of characteristic ions highlighted in the OPLS-DA S-plot comparing MES-SSW extracts to other SSW extracts.

VIP	*m*/*z*	R_t_ (min)	Observed Features (Ionic Species)	Molecular Formula for M	∆ppm	UV-vis Absorption (λ_max_ nm)	Putative Annotation
**8.95**	231.1225	6.52	[M+H]^+^	C_11_H_18_O_5_	−3.24	238	2’-hydroxypestalotin isomer
	253.1050		[M+Na]^+^				
	269.0800		[M+K]^+^				
	483.2219		[2M+Na]^+^				
**7.95**	211.0581	6.58	[M+Na]^+^	C_8_H_12_O_5_	−0.68	237	Unknown pyran-2-one derivative
**5.45**	213.1139	9.82	[M+H]^+^	C_11_H_16_O_4_	5.71	279	Dehydroxypestalotin isomer
**4.59**	129.0549	2.18	[M+H]^+^	C_6_H_10_O_4_	−2.09	237	5,6-dihydro-4-methoxy-2*H*-pyran-2-one
**4.53**	215.1281	9.08	[M+H]^+^	C_11_H_18_O_4_	−1.09	237	Pestalotin isomer
	237.1117		[M+Na]^+^				
**3.57**	251.0905	6.68	[M+Na]^+^	C_11_H_16_O_5_	5.8	275	LL-P880*γ* isomer
**3.50**	147.0654	2.13	[M+H]^+^	C_6_H_10_O_4_	−4.99	237	5-hydroxy-3-methoxy-2-pentenoic acid
**3.24**	251.0905	11.56	[M+Na]^+^	C_11_H_16_O_5_	1.82	275	LL-P880*γ* isomer

**Table 3:** ^1^H (500 MHz) and ^13^C NMR (125 MHz) data for compounds **1**-**7** and the reference compound (6*S*,1’*S*,2’*R*)-LL-P880*β* (in CDCl_3_).

Position	1		(6*S*,1’*S*,2’*R*)-LL-P880*β*		2		3	
	δ_C,_ type	δ_H_ (*J* in Hz)	δ_C,_ type	δ_H_ (*J* in Hz)	δ_C,_ type	δ_H_ (*J* in Hz)	δ_C,_ type	δ_H_ (*J* in Hz)
2	166.73 C	-	166.58 C		167.03 C	-	167.18 C	-
3	89.95 CH	5.16, s	89.73 CH	5.14 (d, 1.5)	89.51 CH	5.12, s	89.55 CH	5.11, s
4	172.94 C	-	173.40 C		173.94 C	-	173.68 C	-
5	28.84 CH_2_	a. 2.8 (dd, 12.8, 16.7) b. 2.28 (dd, 3.7, 17.0)	29.35 CH_2_	a. 2.89 (ddd, 1.5, 12.9, 17.2) b. 2.32 (dd, 3.7, 16.9)	29.49 CH_2_	a. 3.05 (dd, 13.8, 16.7) b. 2.19 (dd, 2.9, 17.0)	29.30 CH_2_	a. 2.88 (dd, 13.9, 16.2) b. 2.31 (dd, 3.5, 17.2)
6	76.21 CH	4.51, m	77.95 CH	4.52 (dt, 4.0, 4.0, 12.9)	75.41 CH	4.76 (dt, 12.8, 2.3)	77.88 CH	4.5 (dt, 3.9, 4.2, 12.8)
4-OCH_3_	56.18	3.77, s	56.16	3.76, s	56.16	3.76, s	56.18	3.74, s
1’	63.74 CH_2_	a. 3.73 (dd, 4.6, 12.2)	73.80 CH	3.49 (dd, 2.9, 4.2)	71.27 CH	3.37 (dd, 2.0, 6.7)	70.77 CH	3.47 (t, 3.5, 3.9)
		b. 3.91, m						
2’	-	-	70.93 CH	3.80, m	74.13 CH	3.80 (t, 7.1, 7.7)	73.78 CH	3.77 (t, 3.5, 4.3)
3’	-	-	35.94 CH_2_	1.5–1.63, m	35.71 CH_2_	a. 1.66, m b. 1.5, m	35.84 CH_2_	1.5–1.58, m
4’	-	-	18.78 CH_2_	1.38–1.5, m	18.78 CH_2_	a. 1.58, m b. 1.4, m	18.72 CH_2_	1.38–1.5, m
5’	-	-	13.94 CH_3_	0.94 (t, 7.1)	13.98 CH_3_	0.95 (t, 7.1)	13.92 CH_3_	0.92 (t, 6.7)
**Position**	**4**		**5**		**6**		**7**	
	**δ_C,_ type**	**δ_H_ (*J* in Hz)**	**δ_C,_ type**	**δ_H_ (*J* in Hz)**	**δ_C,_ type**	**δ_H_ (*J* in Hz)**	**δ_C,_ type**	**δ_H_ (*J* in Hz)**
2	166.58 C		164.12 C		162.7 C		163.75 C	
3	89.81 CH	5.14, s	88.4 CH	5.46 (d, 2.0)	88.35 CH	5.44 (d, 2.7)	89.98 CH	5.53 (d, 2.3)
4	173.32 C		171.11 C		171.18 C		170.56 C	
5	29.55 CH_2_	a. 2.97 (dd, 13.8, 17.3) b. 2.27 (dd, 3.9, 17.3)	100.45 CH	6.16 (d, 2.0)	100.19 CH	6.14 (d, 2.0)	103.13 CH	6.01, m
6	75.94 CH	4.51 (dt, 3.2, 3.2, 12.8)	163.19 C	-	167.3 C	-	150.90 CH	7.35 (d, 6.1)
4-OCH_3_	56.18	3.76, s	55.99	3.83, s	55.96	3.82, s	55.51	3.81, s
1’	74.79 CH	3.49 (dd, 2.0, 6.1)	73.44 CH	4.44 (d, 4.0)	73.75 CH	4.51, br,m	-	-
2’	72.62 CH	4.32 (t, 6.4)	72.56 CH	4.02, m	72.98 CH	4.51, br,m	-	-
3’	129.24 CH	5.53 (dd, 7.7, 15.7)	33.24 CH_2_	1.43, m	127.1 CH	5.5 (dd, 1.3, 2.0, 15.5)	-	-
4’	130.60 CH	5.88, m	18.91 CH_2_	a. 1.54, m b. 1.35, m	131.48 CH	5.83 (dq, 6.7, 15.5)	-	-
5’	17.88 CH_3_	1.74 (d, 6.4)	13.91 CH_3_	0.93 (t, 7.4)	17.9 CH_3_	1.71 (dd, 1.3, 6.7)	-	-

## Data Availability

Further data are available on request from the corresponding authors.

## Supplementary Materials

The following are available online at https://www.mdpi.com/article/10.3390/md19070378/s1: Figure S1: Morphology of strain *P. restrictum* on 14 different media after 10 days of growth (seven media, two salinity); Figure S2: (a) PCA of OSMAC data in positive mode of 14 media (seven media, two salinity, four replicates) with QC (quality control); Figure S3: (a) OPLS-DA score-plot of YES-SSW versus the six other SSW extracts (positive ionization, n = 4; R2X cum 0.366 and R2Y cum 0.98, Q2 cum 0.703; CV-ANOVA (*p*-value = 5.37 × 10^−4^) and (b) the corresponding S-plot: features highlighted in yellow correspond to discriminatory ions with VIP > 3; Table S1: Identification of representative characteristics ions of OPLS-DA separation YES-SSW media from other; Table S2: Dereplication from HRMS/MS data sets of the crude ethyl acetate extract of *P. restrictum* MMS417 on MES-SSW medium; Figure S4: Annotation of cluster 3—light blue nodes represent features with values VIP > 1; Figure S5: LC-(+)-ESIHRMS spectrum and UV-Vis spectrum of compound **1** with corresponding structure; Figure S6: LC-(+)-ESI HRMS spectrum and UV-Vis spectrum of compound **2** with corresponding structure; Figure S7: LC-(+)-ESIHRMS spectrum and UV-Vis spectrum of compound **3** with corresponding structure; Figure S8: LC-(+)-ESIHRMS spectrum and UV-Vis spectrum of compound **4** with corresponding structure; Figure S9: LC-(±)-ESIHRMS spectrum and UV-Vis spectrum of compound **5** with corresponding structure; Figure S10: LC-(+)-ESIHRMS spectrum and UV-Vis spectrum of compound **6** with corresponding structure; Figure S11: LC-(+)-ESIHRMS spectrum and UV-Vis spectrum of compound **7** with corresponding structure; Figure S12–S50: 1D and 2D NMR spectra of compounds **1**–**7**.
