# Supplementary material for: Untargeted Metabolomics Approach for the Discovery of Environment-Related Pyran-2-Ones Chemodiversity in a Marine-Sourced Penicillium restrictum
Source: Mar Drugs. 2021 Jun 29;19(7):378. doi: 10.3390/md19070378 (PMC8305465; doi:10.3390/md19070378)
Supplement: Supplementary file 1 [file marinedrugs-19-00378-s001.zip › marinedrugs-1264397-supplementary.pdf]

Untargeted metabolomics approach for the discovery of environmentally dependantpyran-2-ones chemodiversity in a marine-sourced *Penicillium restrictum*

Van-Tuyen Le[1], Samuel Bertrand[1], Thibaut Robiou du Pont[1], Fabrice Fleury[2], Nathalie Caroff[3], Sandra Bourgeade-Delmas[4], Cedric Logé[5], Gregory Genta-Jouve[6], and Olivier Grovel[1]*

[1]EA 2160 - Mer Molécules Santé, Université de Nantes, 9 rue Bias BP 53508, 44035 Nantes-Cedex 1, France.
[2]Group of Mechanism and Regulation of DNA Repair and IMPACT Platform, UFIP UMR CNRS 6286, University of Nantes, 44322, Nantes, France
[3]EA3826, Université de Nantes, IRS2 Nantes Biotech, Nantes-Cedex1, F-44100, France
[4]UMR 125 PharmaDev, Université de Toulouse, IRD, Toulouse 31400, France
[5]EA1155-IICIMED, Université de Nantes, IRS2 Nantes Biotech, Nantes-Cedex1, F-44100, France
[6] C-TAC, UMR 8638 CNRS, Faculté de Pharmacie de Paris, Paris-Descartes University, Sorbonne, 4 Avenue de l'Observatoire, 75006 Paris, France.
* Correspondence: olivier.grovel@univ-nantes.fr.

Table of Contents

Experimental section....4

Figure S1: Morphology of strain *P. restrictum* on 14 different media after 10 days of growth (7 media, 2 salinity)....4

Figure S2. PCA of OSMAC data in positive mode of 14 media (7 media, 2 salinity, 4 replicates) with QC (quality control). 4

Figure S3. a) OPLS-DA scores-plot of YES-SSW versus the 6 other SSW extracts (positive ionization, n = 4; R2X cum 0.366 and R2Y cum 0.98, Q2 cum 0.703; CV-ANOVA (*p*-value = 5.37e-4)); b) Corresponding S-plot: features highlighted in yellow correspond to discriminatory ions with VIP > 3 5

Table S1. Identification of representative characteristics ions of OPLS-DA separation YES-SSW media from others 5

Table S2: Dereplication from HRMS/MS data sets of the crude extract of *P. restrictum* MMS417 on MES-SSW medium....6

Figure S4. Annotation of cluster 3. Light blue nodes represent features with values VIP > 1 7

Figure S5: LC-(+)-ESIHRMS spectrum in positive mode and UV-Vis spectrum of compound 1 with corresponding structure. 8

Figure S6: LC-(+)-ESIHRMS spectrum in positive mode and UV-Vis spectrum of compound 2 with corresponding structure. 8

Figure S7: LC-(+)-ESIHRMS spectrum and UV-Vis spectrum of compound 3 with corresponding structure. 8

Figure S8: LC-(+)-ESIHRMS spectrum and UV-Vis spectrum of compound 4 with corresponding structure. 9

Figure S9: LC-(±)-ESIHRMS spectrum and UV-Vis spectrum of compound 5 with corresponding structure. 9

Figure S10: LC-(+)-ESIHRMS spectrum and UV-Vis spectrum of compound 6 with corresponding structure. 9

Figure S11: LC-(+)-ESIHRMS spectrum and UV-Vis spectrum of compound 7 with corresponding structure. 10

Figure S12: ^{1}H-NMR spectrum (500 MHz, $CDCl_3$) for 1 11

Figure S13: ^{13}C-NMR spectrum (125 MHz, $CDCl_3$) for 1....11

Figure S14: HSQC spectrum ($CDCl_3$) for 1....12

Figure S15: COSY spectrum ($CDCl_3$) for 1 13

Figure S16: HMBC spectrum ($CDCl_3$) for 1....14

Figure S17: NOESY spectrum ($CDCl_3$) for 1 15

Figure S18: ^{1}H-NMR spectrum (500 MHz, $CDCl_3$) for 2 16

Figure S19: ^{13}C-NMR spectrum (125 MHz, $CDCl_3$) of 2 16

Figure S20: HSQC spectrum ($CDCl_3$) for 2....17

Figure S21: HMBC spectrum ($CDCl_3$) for 2....18

Figure S22: COSY spectrum ($CDCl_3$) for 2 19

Figure S23: NOESY spectrum ($CDCl_3$) for 2 20

Figure S24: ^{1}H-NMR spectrum (500 MHz, $CDCl_3$) for 3 21

Figure S25: ^{13}C-NMR spectrum (125 MHz, $CDCl_3$) for 3....22

Figure S26: HSQC spectrum ($CDCl_3$) for 3....23

Figure S27: HMBC spectrum ($CDCl_3$) for 3....24

Figure S28: COSY spectrum ($CDCl_3$) for 3 25

Figure S29: NOESY spectrum ($CDCl_3$) for 3 26

Figure S30: ^{1}H-NMR spectrum (500 MHz, $CDCl_3$) for 4 27

Figure S31: ^{13}C-NMR spectrum (125 MHz, $CDCl_3$) for 4....27

Figure S32: COSY spectrum ($CDCl_3$) for 4 28

Figure S33: HSQC spectrum ($CDCl_3$) for 4....29

Figure S34: HMBC spectrum ($CDCl_3$) for 4....30

Figure S35: NOESY spectrum ($CDCl_3$) for 4 31

Figure S36: ^{1}H-NMR spectrum (500 MHz, $CDCl_3$) for 5 32

Figure S37: ^{13}C-NMR spectrum (125 MHz, $CDCl_3$) for 5 33
Figure S38: HSQC spectrum ($CDCl_3$) for 5 34
Figure S39: HMBC spectrum ($CDCl_3$) for 5 35
Figure S40: COSY spectrum ($CDCl_3$) for 5 36
Figure S41: NOESY spectrum ($CDCl_3$) for 5 37
Figure S42: ^{1}H-NMR spectrum (500 MHz, $CDCl_3$) for 6 38
Figure S43: ^{13}C-NMR spectrum (125 MHz, $CDCl_3$) for 6 39
Figure S44: COSY spectrum ($CDCl_3$) for 6 40
Figure S45: NOESY spectrum ($CDCl_3$) for 6 41
Figure S46: HSQC spectrum ($CDCl_3$) for 6 42
Figure S47: HMBC spectrum ($CDCl_3$) for 6 43
Figure S48: ^{1}H-NMR spectrum (500 MHz, $CDCl_3$) for 7-8 44
Figure S49: ^{13}C-NMR spectrum (125 MHz, $CDCl_3$) for 7-8 44
Figure S50: HSQC spectrum ($CDCl_3$) for 7-8 45
References 46

Experimental section

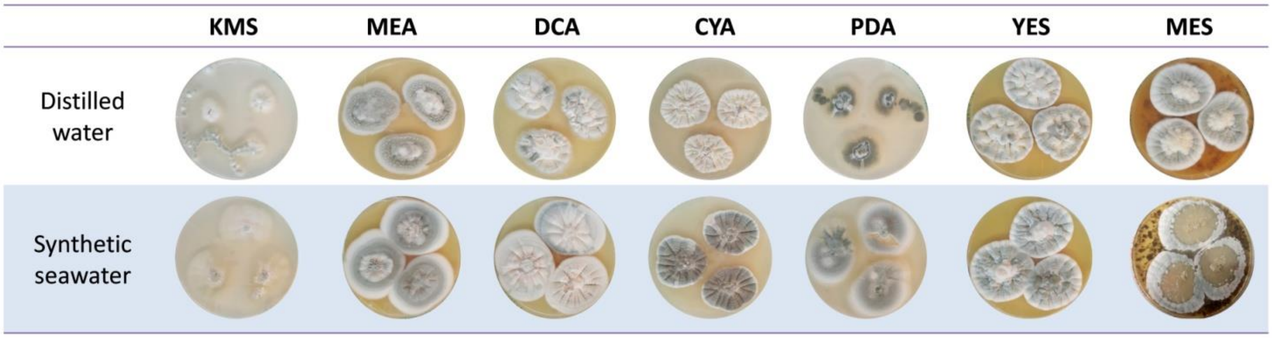

Figure S1: Morphology of strain *P. restrictum* on 14 different media after 10 days of growth (7 media, 2 salinity).

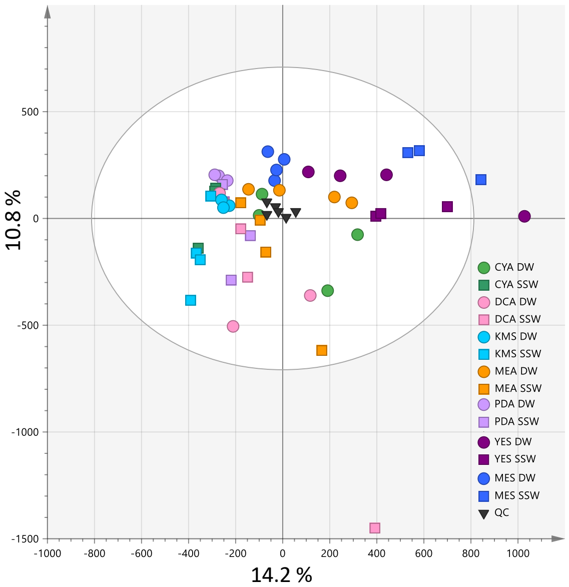

Figure S2. PCA of OSMAC data in positive mode of 14 media (7 media, 2 salinity, 4 replicates) with QC (quality control).

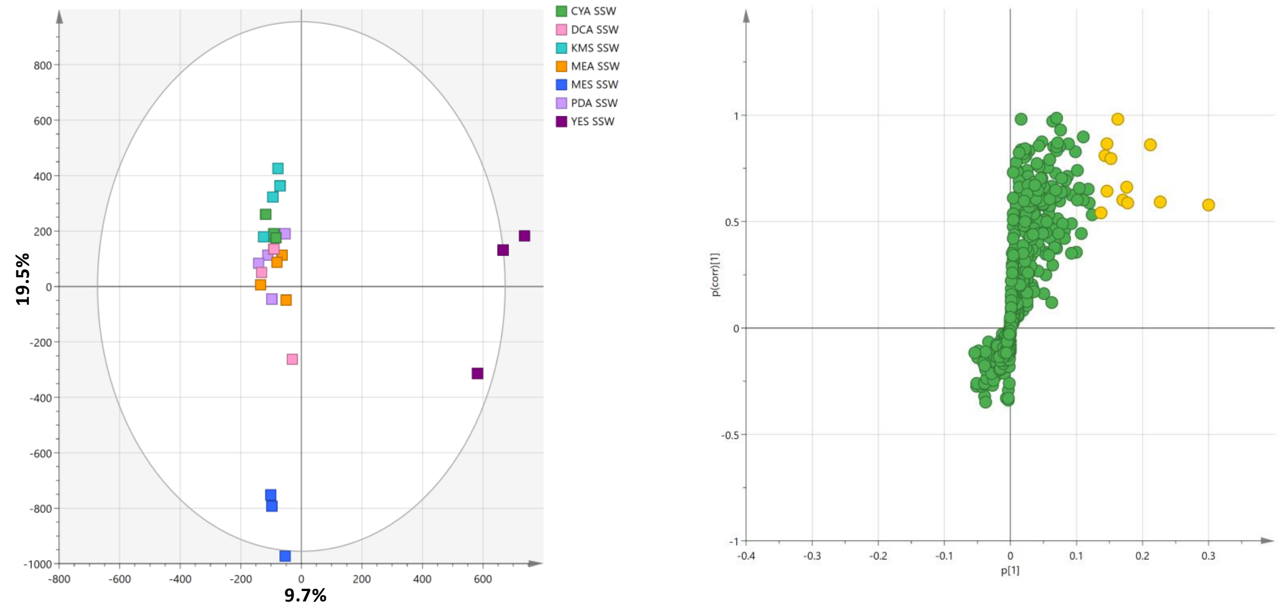

Figure S3. a) OPLS-DA scores-plot of YES-SSW versus the 6 other SSW extracts (positive ionization, n = 4; R2X cum 0.366 and R2Y cum 0.98, Q2 cum 0.703; CV-ANOVA (p-value = 5.37e-4)); b) Corresponding S-plot: features highlighted in yellow correspond to discriminatory ions with VIP > 3

Table S1. Identification of representative characteristics ions of OPLS-DA separation YES-SSW media from others

Vip	*m/z*	R_t (min)	Observed features (ionic species)	Molecular formula for M	Δppm	UV-Vis absorption (λ_{max} nm)	Putative annotation
**8.32**	253.1050	6.52	$[M+Na]^+$	$C_{11}H_{18}O_5$	-0.76	238	2′-hydroxy pestalotin and isomers
	269.0807		$[M+K]^+$				
	483.2219		$[2M+Na]^+$				
**5.1**	431.2787	22.98	$[M+H]^+$	$C_{26}H_{38}O_5$	-2.43	n.d	Simplicissin
**4.38**	486.3583	23.87	$[M+H]^+$	$C_{30}H_{47}NO_4$	-0.07	n.d	Melearoride A
	508.3407		$[M+Na]^+$				
**3.81**	231.1239	4.66	$[M+H]^+$	$C_{11}H_{18}O_5$	1.95	238	2′-hydroxypestalotin and isomers
**3.69**	472.3422	22.27	$[M+H]^+$	$C_{29}H_{45}NO_4$	-1.02	n.d	*N*-demethylmelearoride A
	494.3197		$[M+Na]^+$				
**3.57**	509.3433	23.91	$[M+Na]^+$	$C_{27}H_{50}O_7$	-4.17	n.d	n.d
**3.48**	506.3252	23.23	$[M+Na]^+$	$C_{30}H_{45}NO_4$	1.13	n.d	n.d

n.d.: not hit from fungal natural product databases.

Table S2: Dereplication from HRMS/MS data sets of the crude extract of *P. restrictum* MMS417 on MES-SSW medium

R_t (min)	*m/z*	Adduct type	Molecular Formula	Possible Annotations	Reference
2.13	147.0654	$[M+H]^+$	$C_6H_{10}O_4$	5-Hydroxy-3-methoxy-2-pentenoic acid	[1,2]
2.18	129.0549	$[M+H]^+$	$C_6H_8O_3$	5,6-Dihydro-4-methoxy-2*H*-pyran-2-one	[3]
4.65	231.1225	$[M+H]^+$	$C_{11}H_{18}O_5$	2'-Hydroxy pestalotine isomers	[4,5]
5.66	229.1047	$[M+H]^+$	$C_{11}H_{16}O_5$	LL-P880γ isomers	[6]
5.88	185.0845	$[M+H]^+$	$C_9H_{12}O_4$	8-Macommelinol	[7]
6.09	229.1048	$[M+H]^+$	$C_{11}H_{16}O_5$	LL-P880γ isomers	[6]
6.52	231.1237	$[M+H]^+$	$C_{11}H_{18}O_5$	2'-Hydroxy pestalotine isomers	[4,5]
6.58	211.0581	$[M+H]^+$	$C_{10}H_{10}O_5$	Pyran-2-one derivative	
7.29	229.1049	$[M+H]^+$	$C_{11}H_{16}O_5$	LL-P880γ isomers	[6]
9.08	215.1281	$[M+H]^+$	$C_{11}H_{18}O_4$	(-)-Pestalotin	[4,5]
9.71	235.0963	$[M+K]^+$	$C_{11}H_{18}O_3$	6-Pentyl-4-methoxy-2*H*-pyran-2-one	[8]
9.09	213.1132	$[M+H]^+$	$C_{11}H_{16}O_4$	Dehydropestalotin isomers	[9,10]
9.82	213.1139	$[M+H]^+$	$C_{11}H_{16}O_4$	Dehydropestalotin isomers	[9,10]
10.03	213.1132	$[M+H]^+$	$C_{11}H_{16}O_4$	Pyran-2-one derivative	
13.75	197.1154	$[M+H]^+$	$C_{11}H_{18}O_3$	6-Pentyl-4-methoxy-2*H*-pyran-2-one	[8]
15.08	371.2819	$[M+H]^+$	$C_{21}H_{38}O_5$	n.d.	
19.41	484.2959	$[M+Na]^+$	$C_{27}H_{43}NO_5$	PF 1163B	[11]
19.62	467.3157	$[M+Na]^+$	$C_{28}H_{44}O_4$	Antibiotic Mer-NF 8054A	[12]
20.72	451.3219	$[M+NH_4]^+$	$C_{25}H_{39}NO_5$	Aspochalasin K	[13]
21.96	349.2359	$[M+H]^+$	$C_{21}H_{32}O_4$	Brassicicene C	[14]
21.97	393.3115	$[M+H]^+$	$C_{28}H_{40}O$	Ergostatetraen-3-one isomers	[15,16]
22.27	472.3422	$[M+H]^+$	$C_{29}H_{45}NO_4$	*N*-demethylmelearoride A	[17]
22.35	441.3029	$[M+H]^+$	$C_{22}H_{40}N_4O_5$	n.d.	
22.64	384.3513	$[M+H]^+$	$C_{23}H_{45}NO_3$	n.d.	
22.98	431.2796	$[M+Na]^+$	$C_{24}H_{40}O_5$	Simplicissin	[18]
23.22	393.3105	$[M+H]^+$	$C_{28}H_{40}O$	Ergostatetraen-3-one isomers	[15,16]
23.41	411.3253	$[M+H]^+$	$C_{28}H_{42}O_2$	3β-Hydroxyergosta-8,14,24(28)-trien-7-one isomers	[19]
23.53	393.3104	$[M+H]^+$	$C_{28}H_{40}O$	Ergostatetraen-3-one isomers	[15,16]
23.77	375.3014	$[M+H]^+$	$C_{28}H_{38}$	Ergostahexaene isomers	[20]
23.81	486.3580	$[M+H]^+$	$C_{30}H_{47}NO_4$	Melearoride A	[21]
23.87	443.3115	$[M+H]^+$	$C_{28}H_{42}O_4$	Paxisterol	[22]
23.87	393.3112	$[M+H]^+$	$C_{28}H_{40}O$	Ergostatetraen-3-one isomers	[15,16]
23.88	522.3563	$[M+Na]^+$	$C_{29}H_{47}NO_5$	*N*-demethyl PF 1163E isomers	
24.07	500.3816	$[M+H]^+$	$C_{29}H_{47}NO_5$	*N*-demethyl PF 1163E isomers	
24.07	411.3284	$[M+H]^+$	$C_{28}H_{42}O_2$	3β-Hydroxyergosta-8,14,24(28)-trien-7-one isomers	[19]
24.08	433.2983	$[M+H]^+$	$C_{26}H_{40}O_5$	Curvicollide B	[23]
24.2	395.3267	[M+H]+	$C_{28}H_{42}O$	Ergostatetraen-3-ol	[24]
24.58	391.2879	$[M+H]^+$	$C_{24}H_{38}O_4$	n.d.	
24.78	375.3015	$[M+H]^+$	$C_{28}H_{38}$	Ergostahexaene isomers	[20]
25.22	514.3834	$[M+H]^+$	$C_{32}H_{51}NO_4$	PF 1163E	[25]

n.d.: not hit from fungal natural product databases.

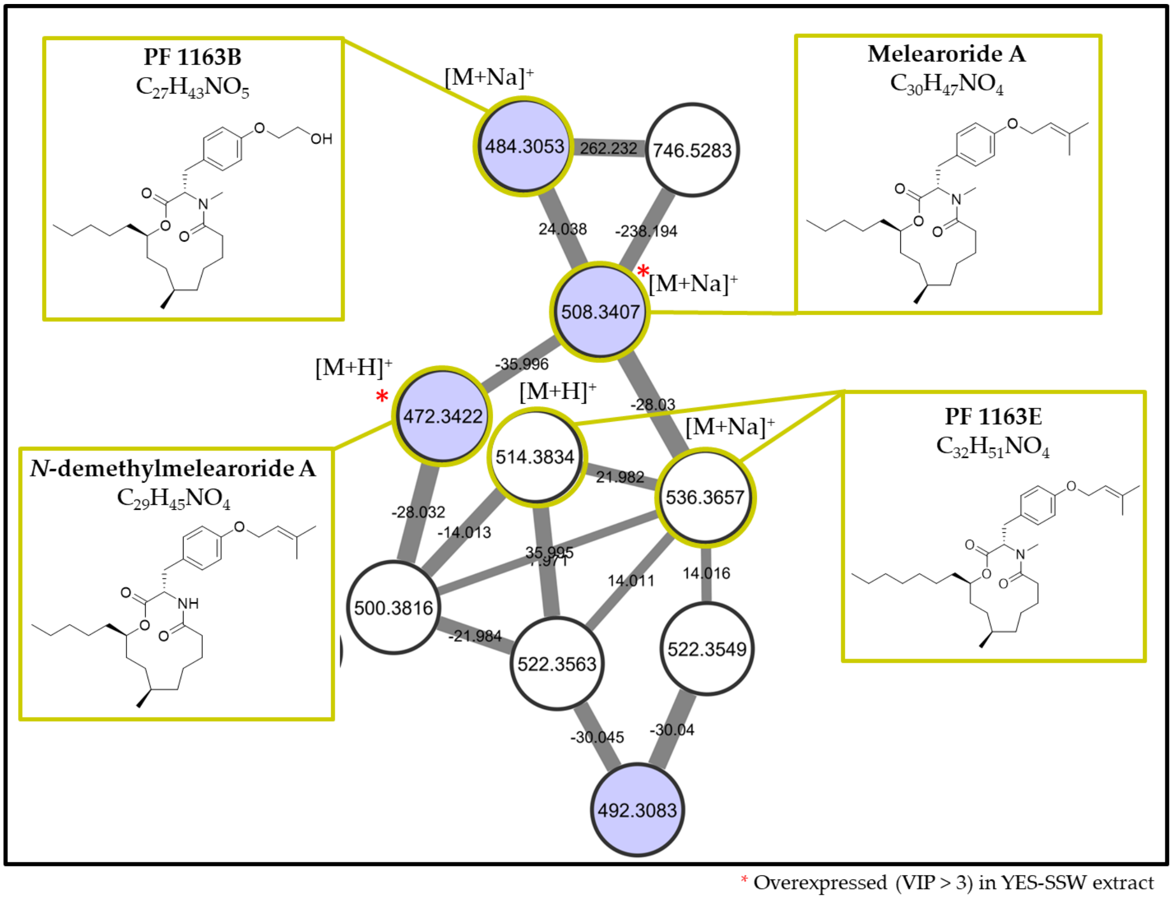

Figure S4. Annotation of cluster 3. Light blue nodes represent features with values VIP > 1

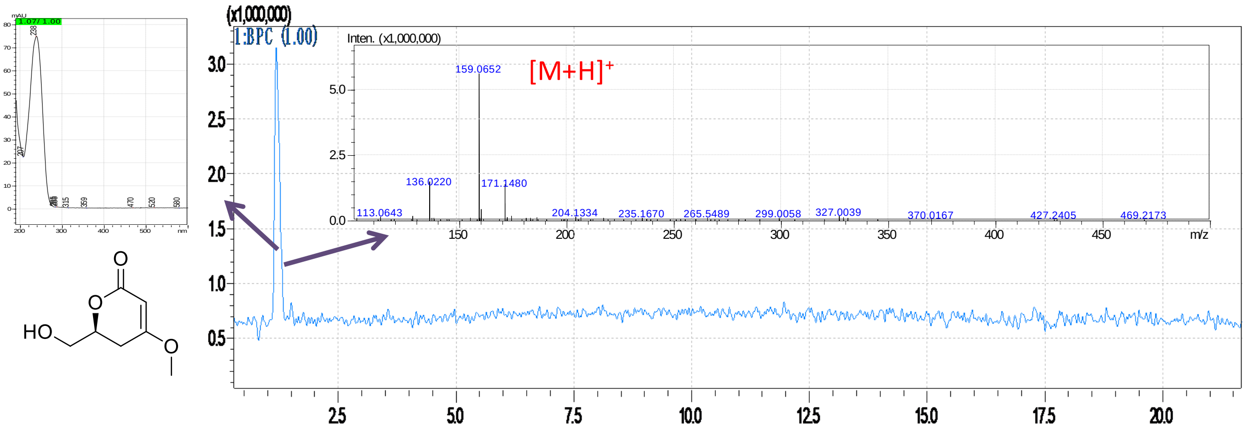

Figure S5: LC-(+)-ESIHRMS spectrum and UV-Vis spectrum of compound **1** with corresponding structure.

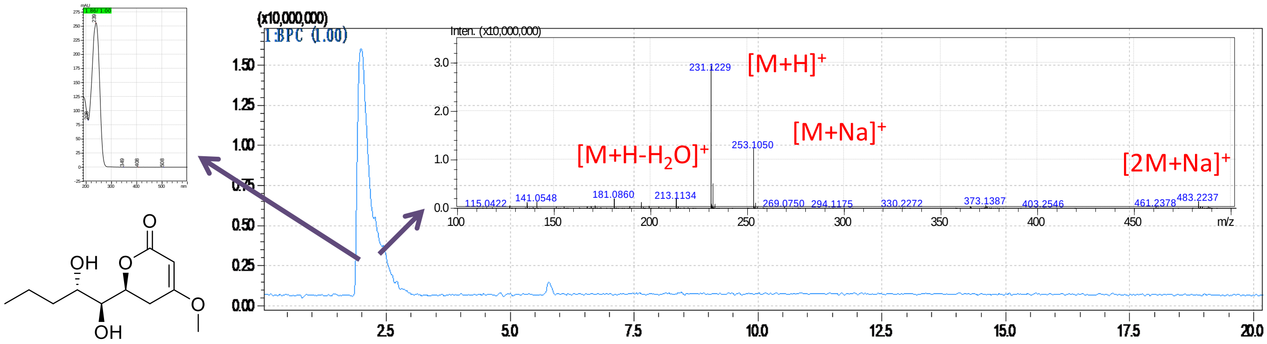

Figure S6: LC-(+)-ESIHRMS spectrum and UV-Vis spectrum of compound **2** with corresponding structure.

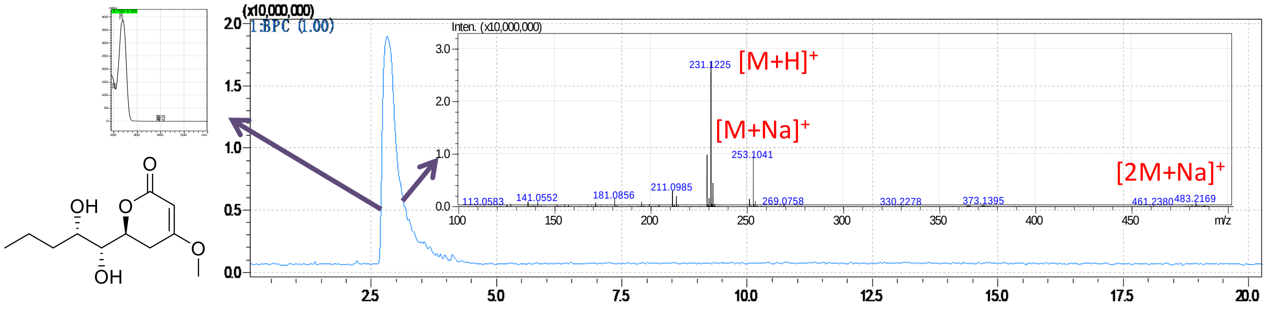

Figure S7: LC-(+)-ESIHRMS spectrum and UV-Vis spectrum of compound **3** with corresponding structure.

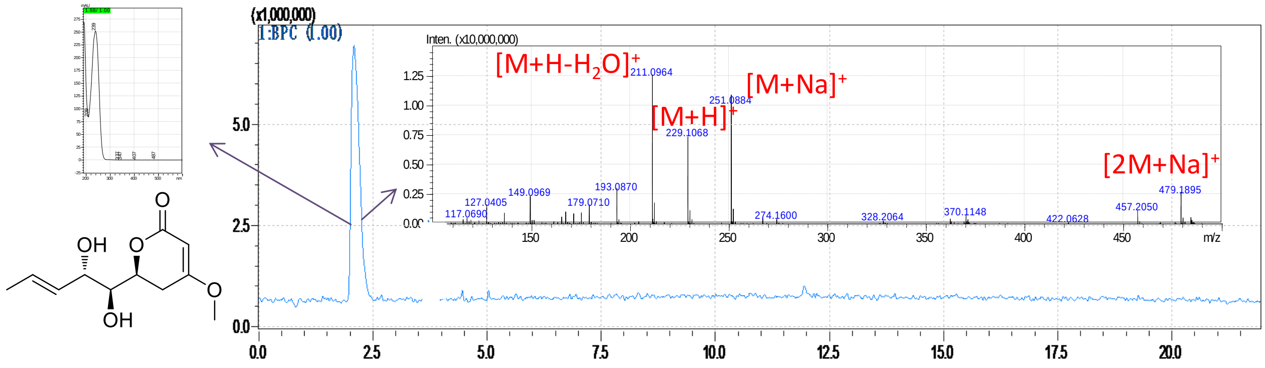

Figure S8: LC-(+)-ESIHRMS spectrum and UV-Vis spectrum of compound **4** with corresponding structure.

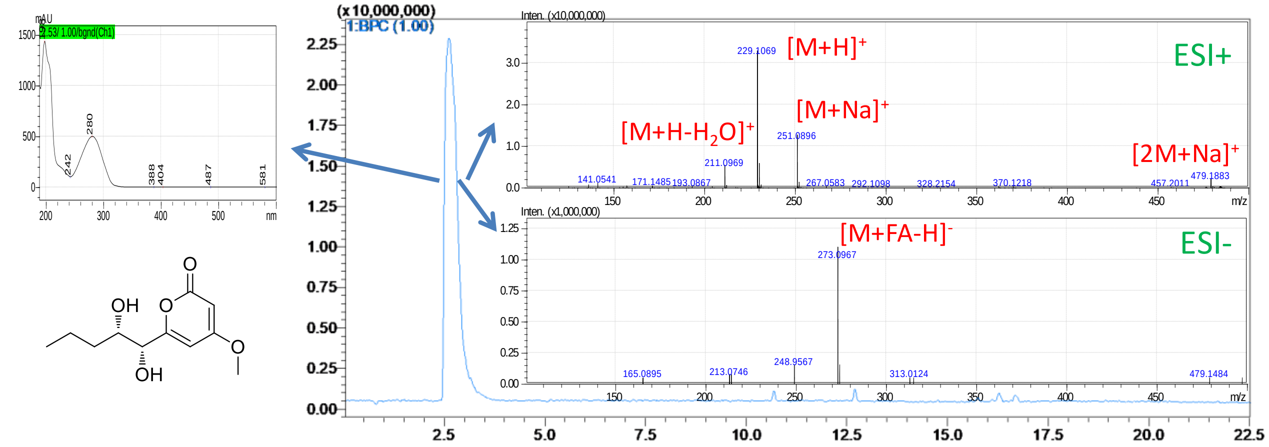

Figure S9: LC-(±)-ESIHRMS spectrum and UV-Vis spectrum of compound **5** with corresponding structure.

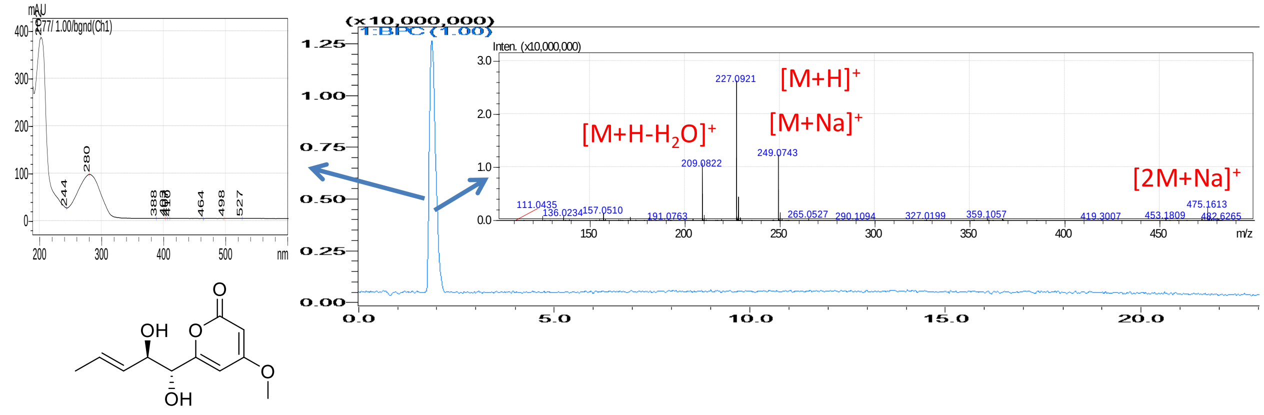

Figure S10: LC-(+)-ESIHRMS spectrum and UV-Vis spectrum of compound **6** with corresponding structure.

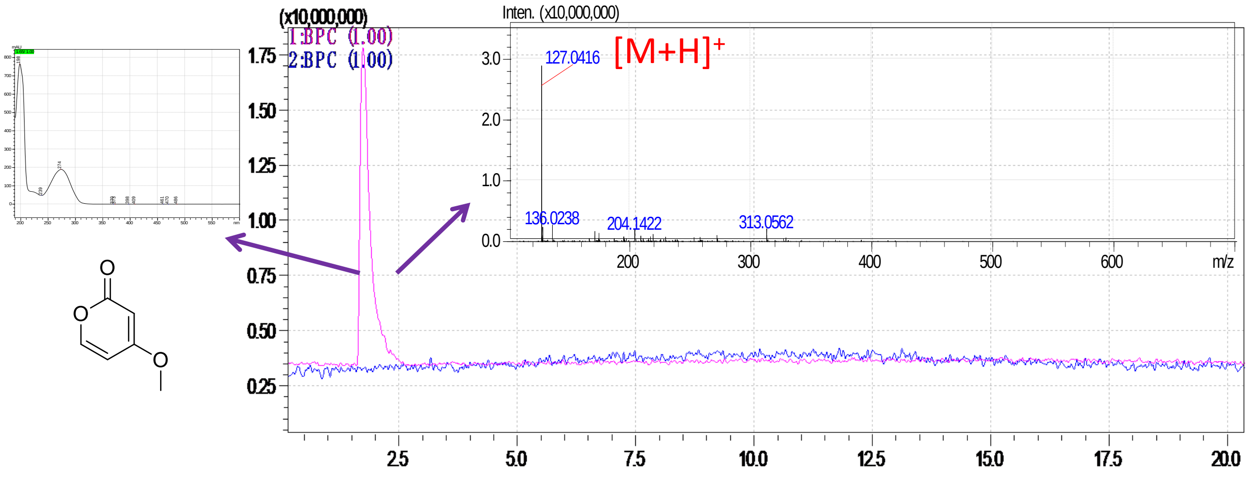

Figure S11: LC-(+)-ESIHRMS spectrum and UV-Vis spectrum of compound **7** with corresponding structure.

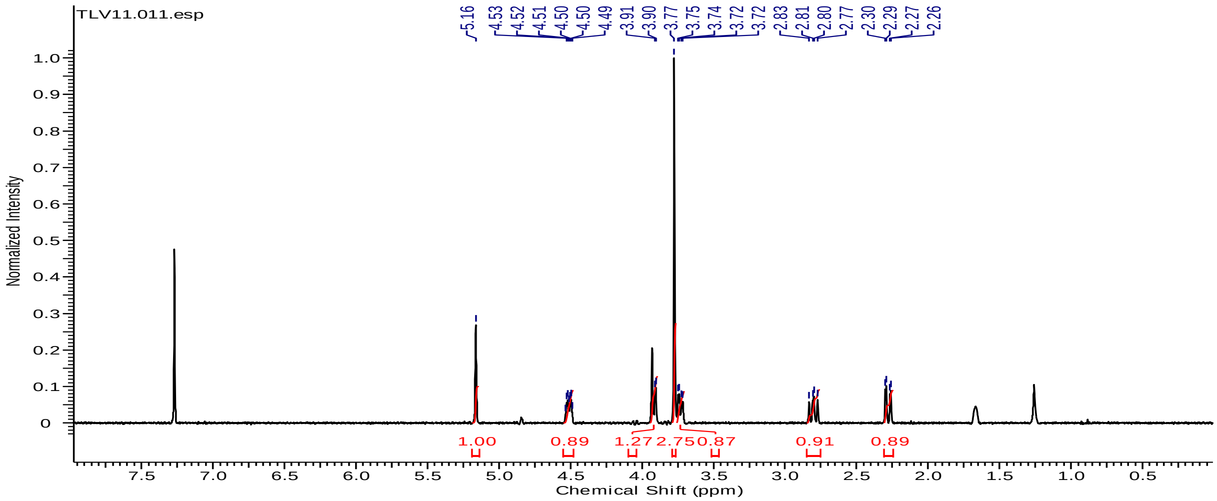

Figure S12: ^{1}H-NMR spectrum (500 MHz, $CDCl_3$) for **1**

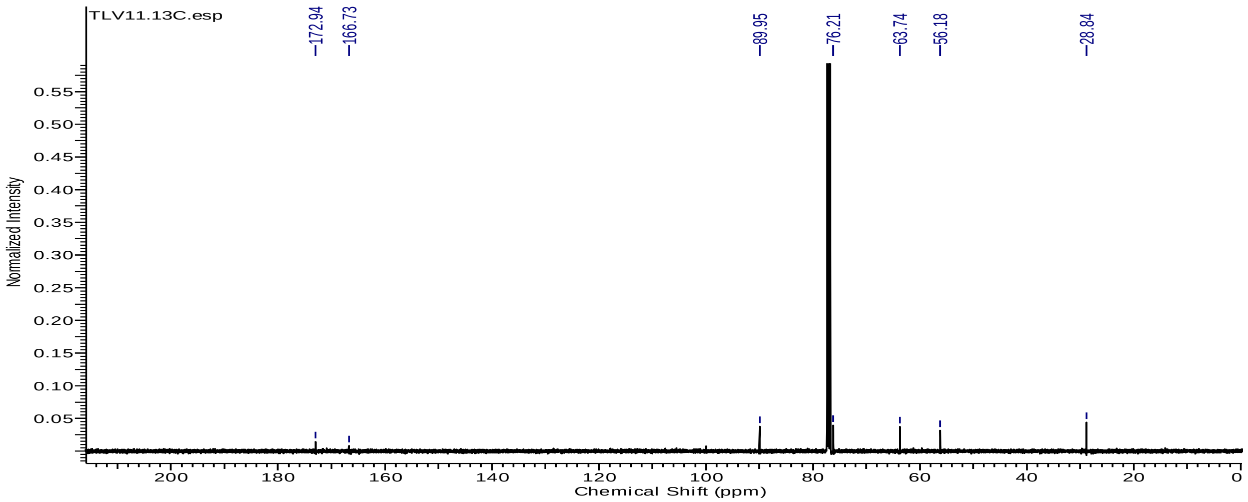

Figure S13: ^{13}C-NMR spectrum (125 MHz, $CDCl_3$) for **1**

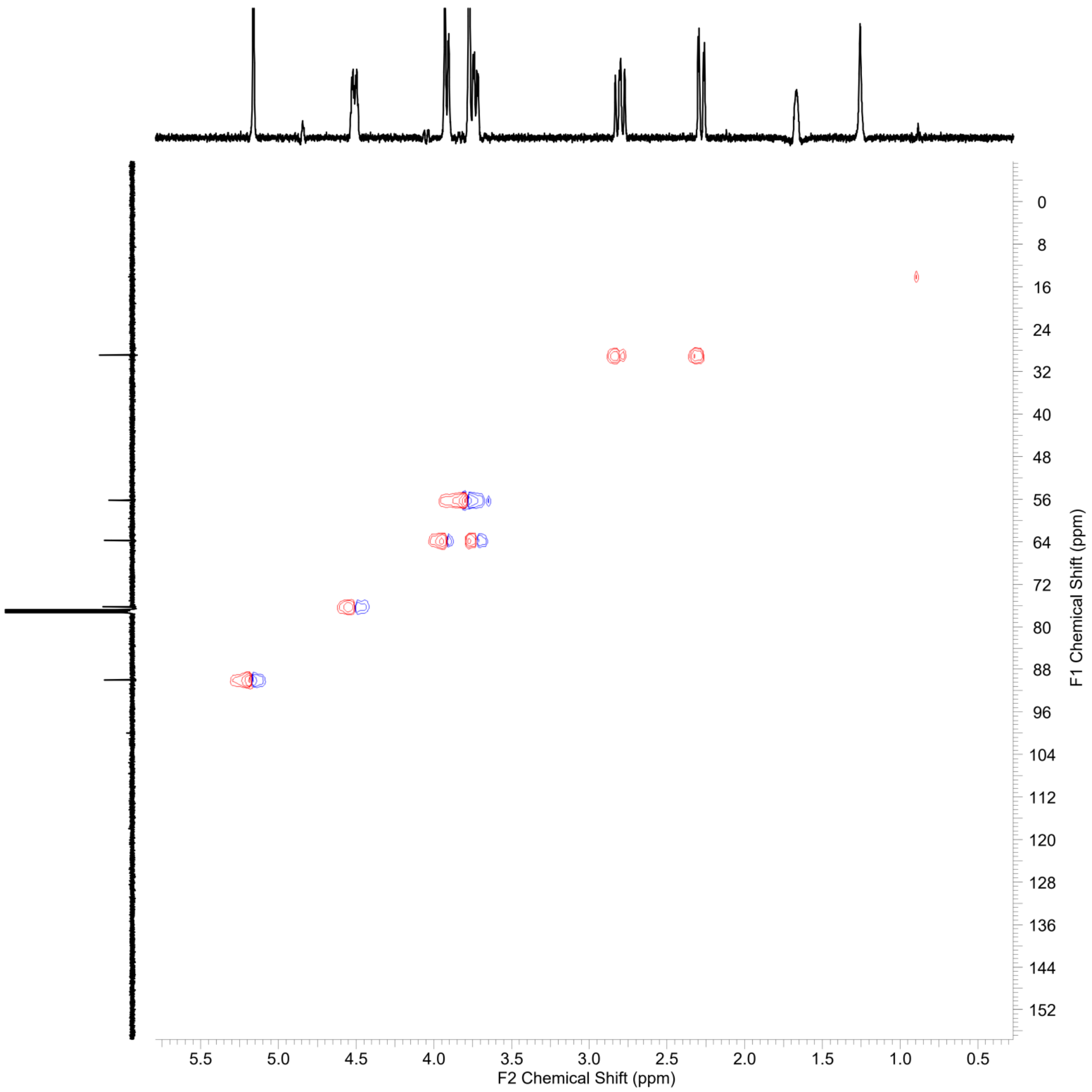

Figure S14: HSQC spectrum ($CDCl_3$) for **1**

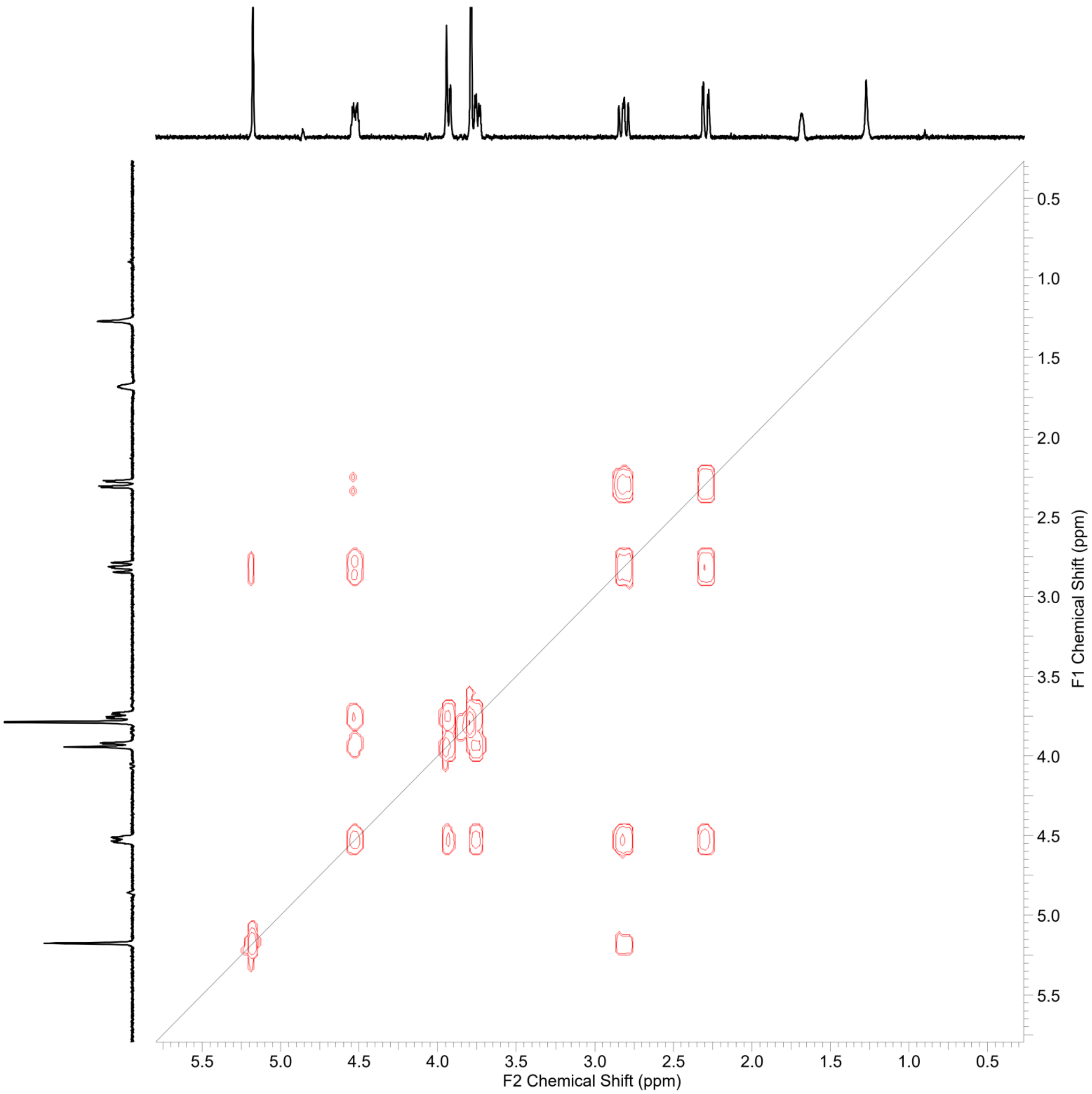

Figure S15: COSY spectrum ($CDCl_3$) for **1**

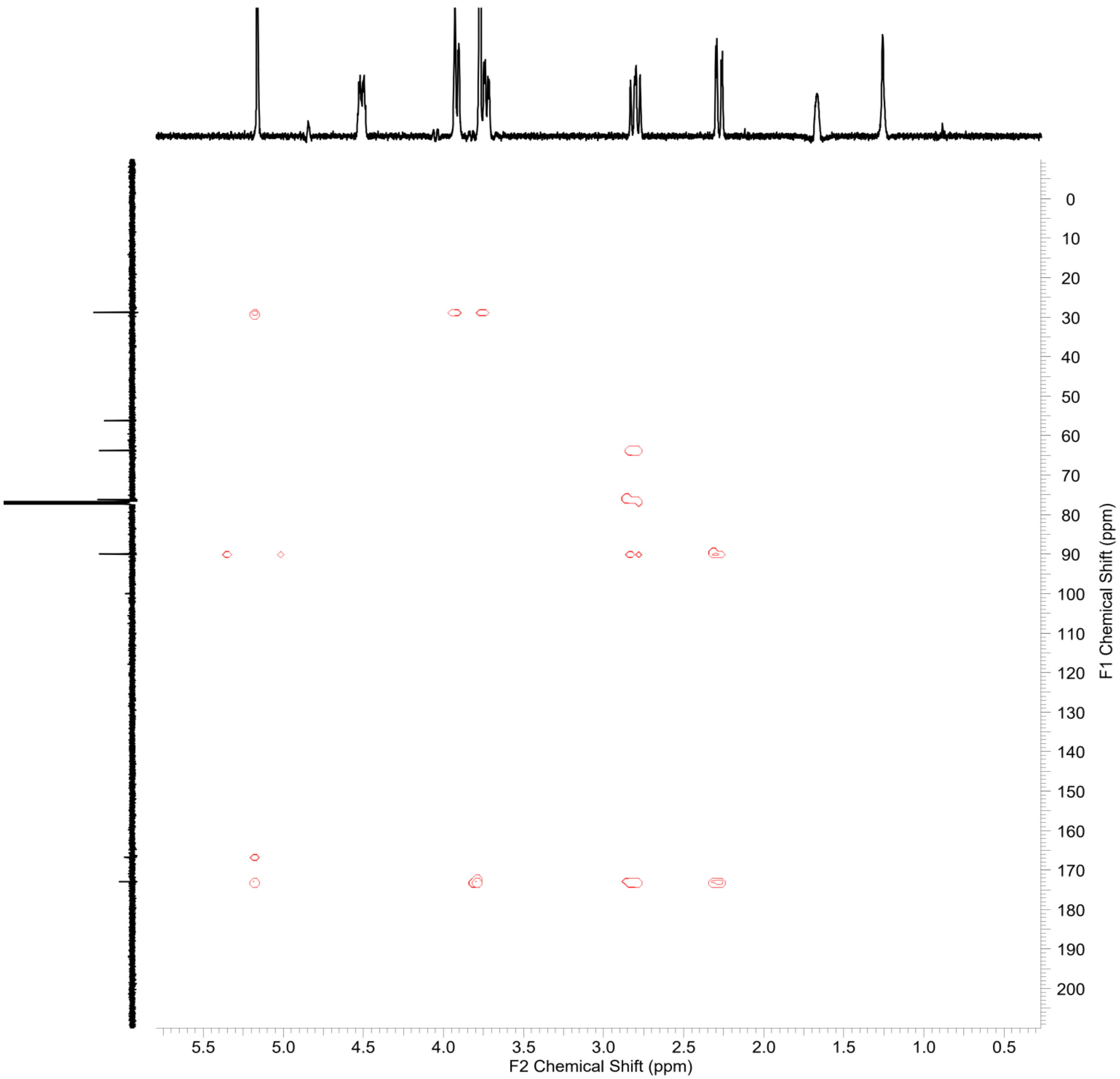

Figure S16: HMBC spectrum ($CDCl_3$) for **1**

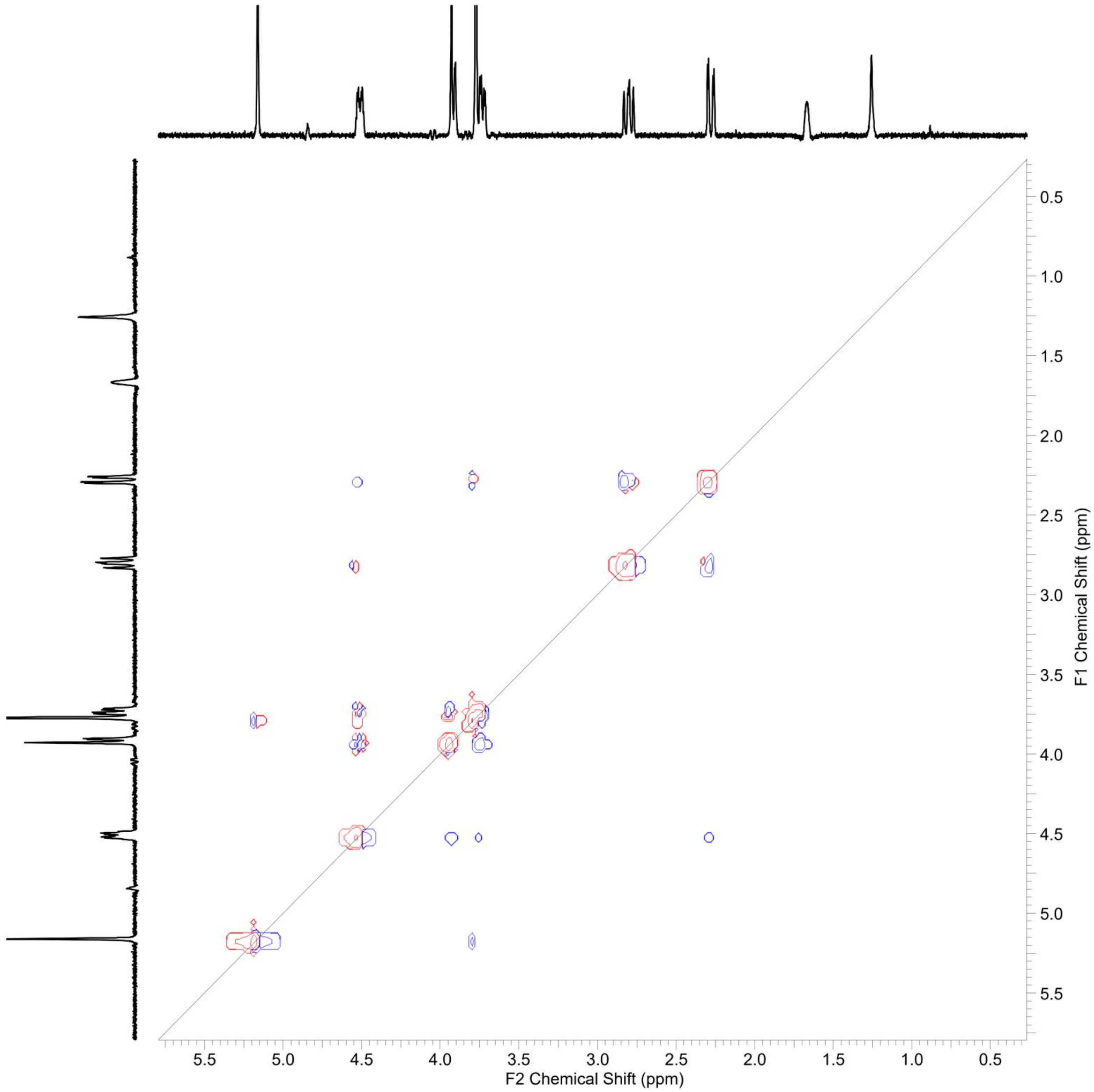

Figure S17: NOESY spectrum ($CDCl_3$) for **1**

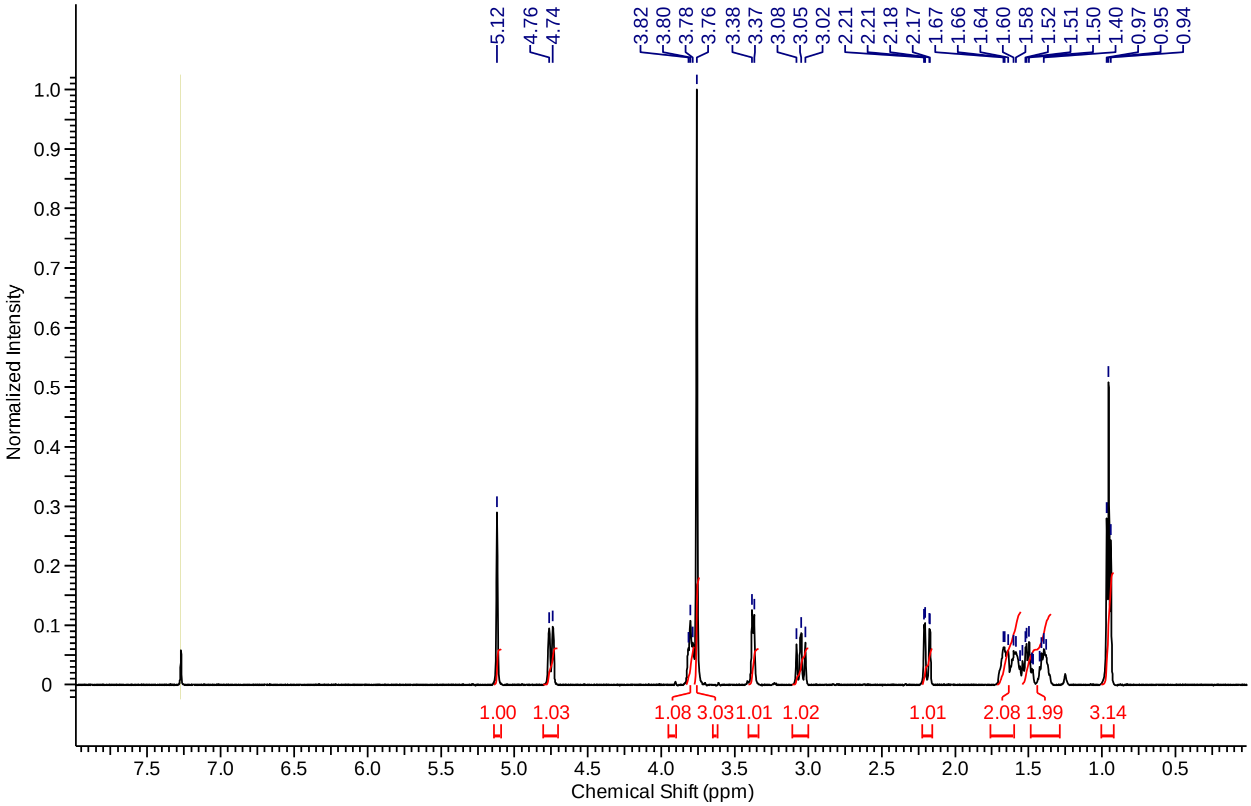

Figure S18: ^{1}H-NMR spectrum (500 MHz, $CDCl_3$) for **2**

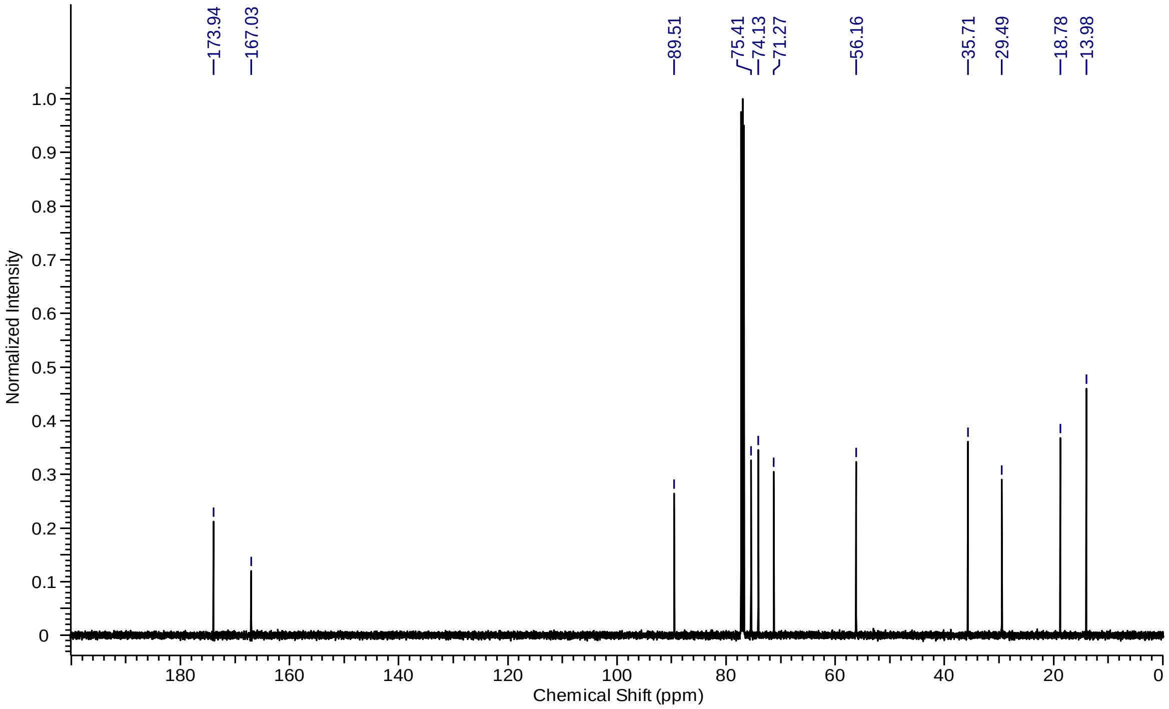

Figure S19: ^{13}C-NMR spectrum (125 MHz, $CDCl_3$) of **2**

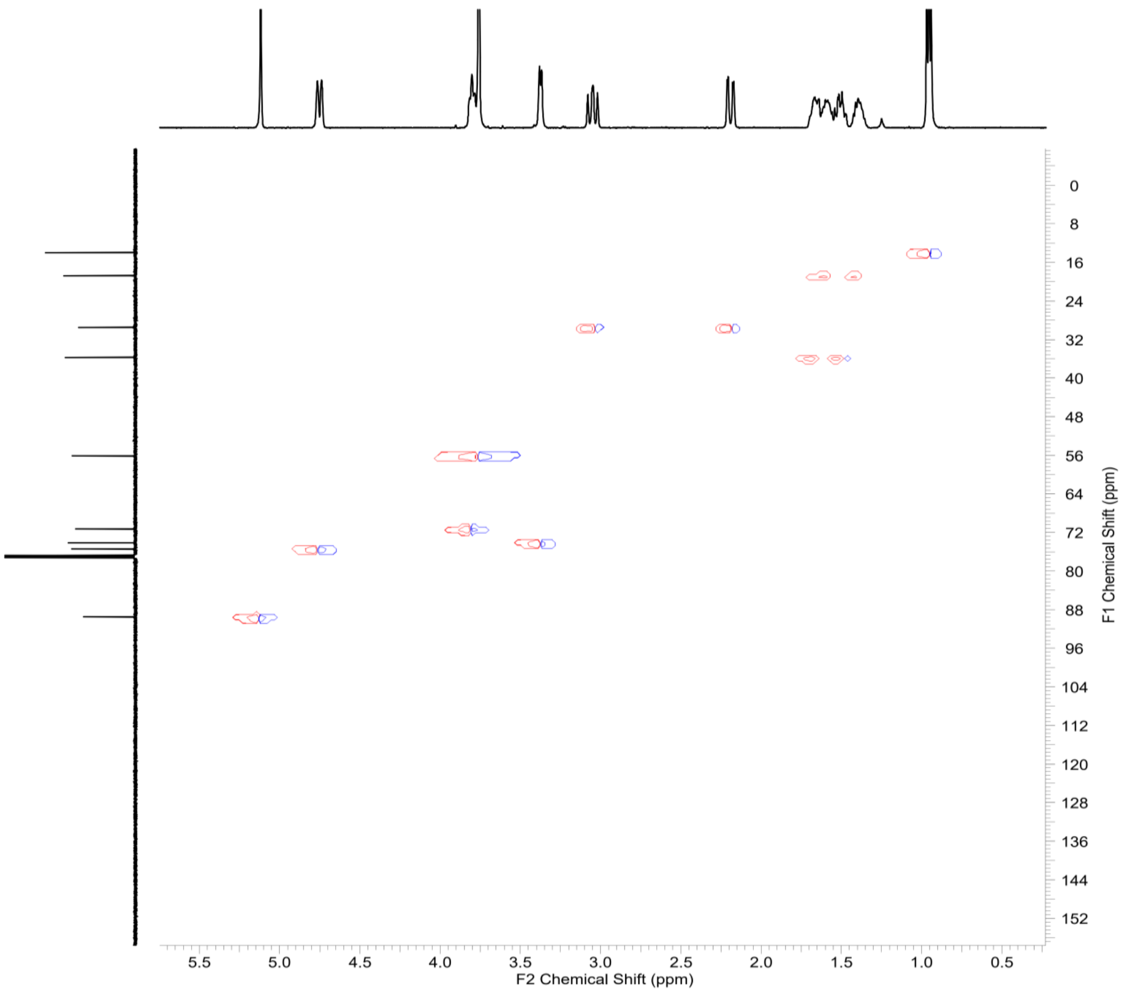

Figure S20: HSQC spectrum ($CDCl_3$) for **2**

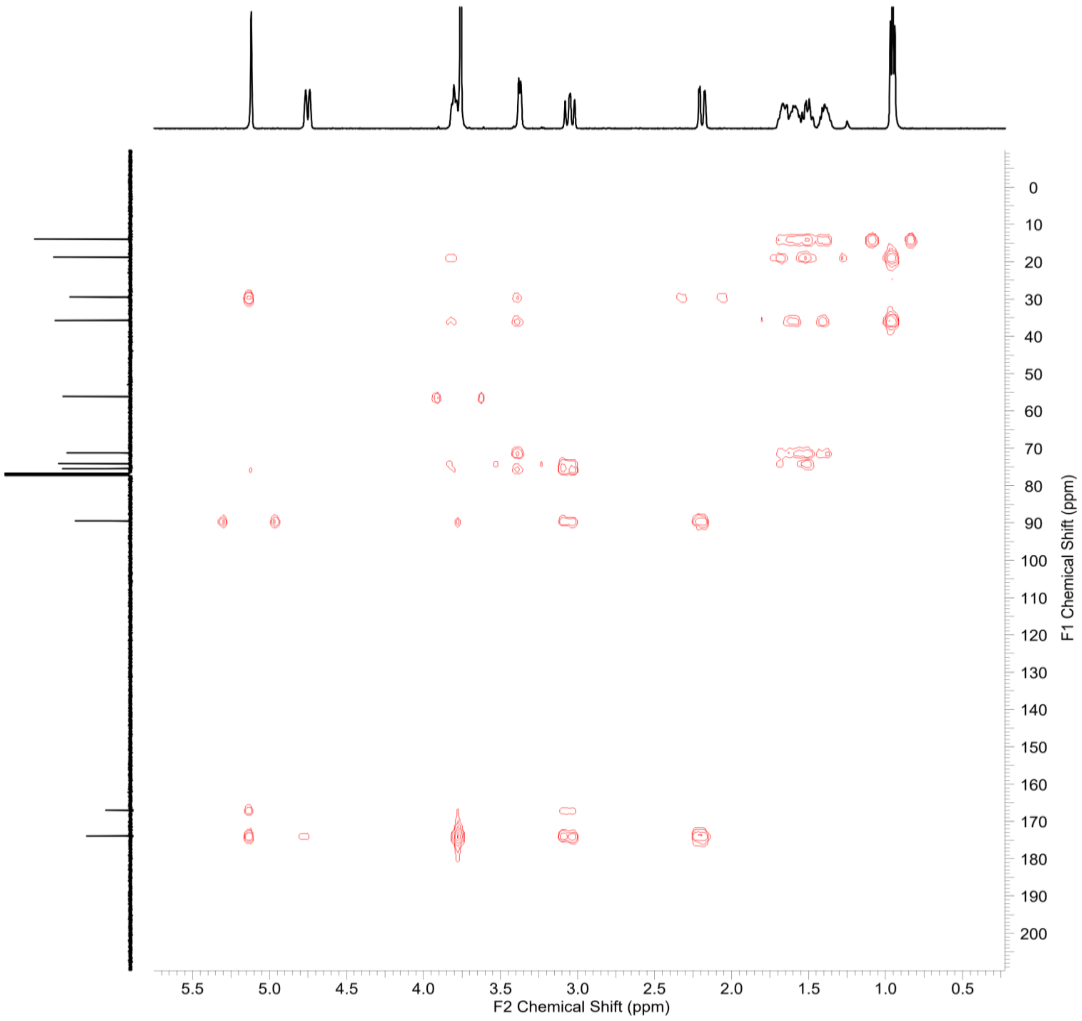

Figure S21: HMBC spectrum ($CDCl_3$) for **2**

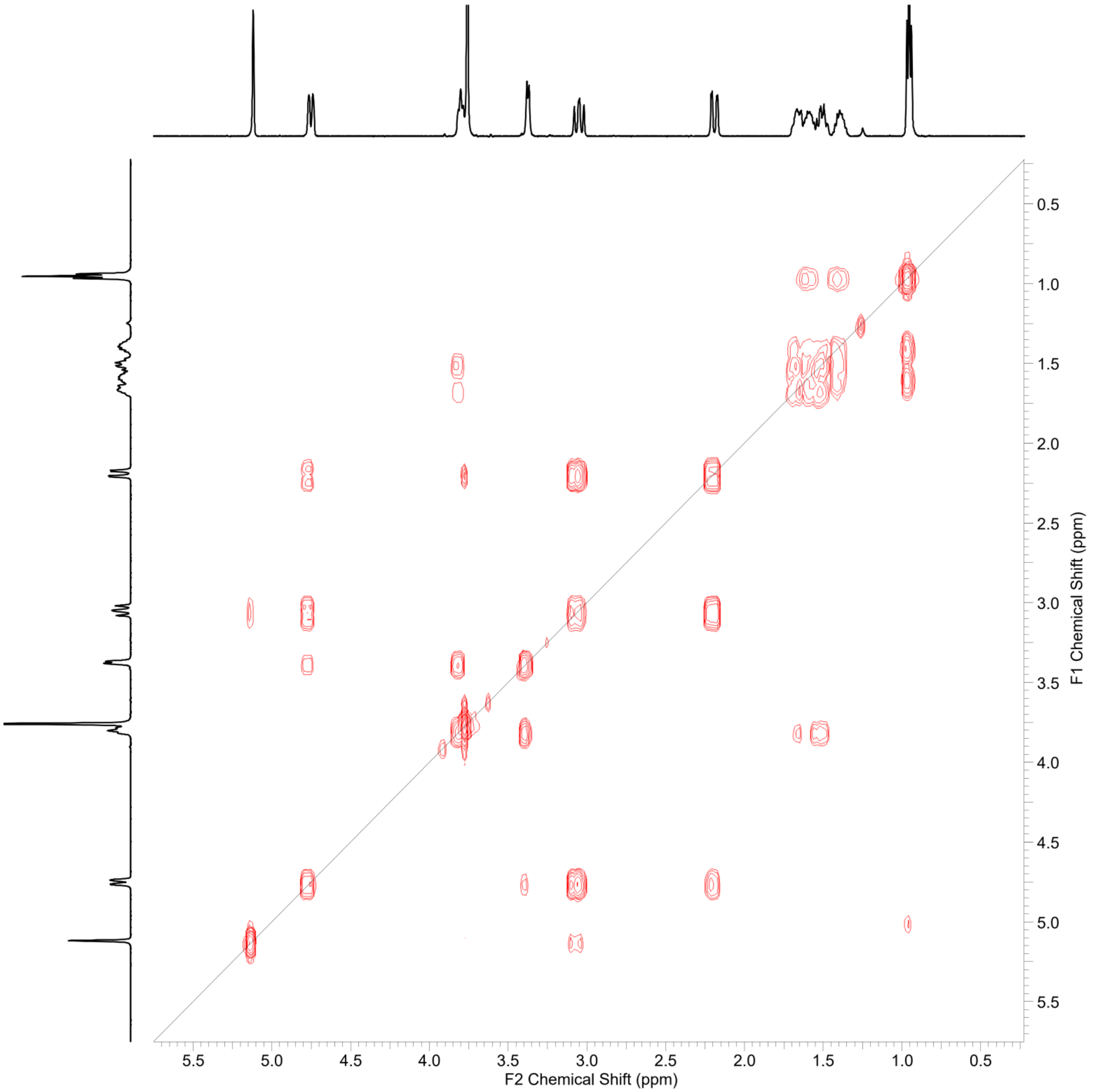

Figure S22: COSY spectrum ($CDCl_3$) for **2**

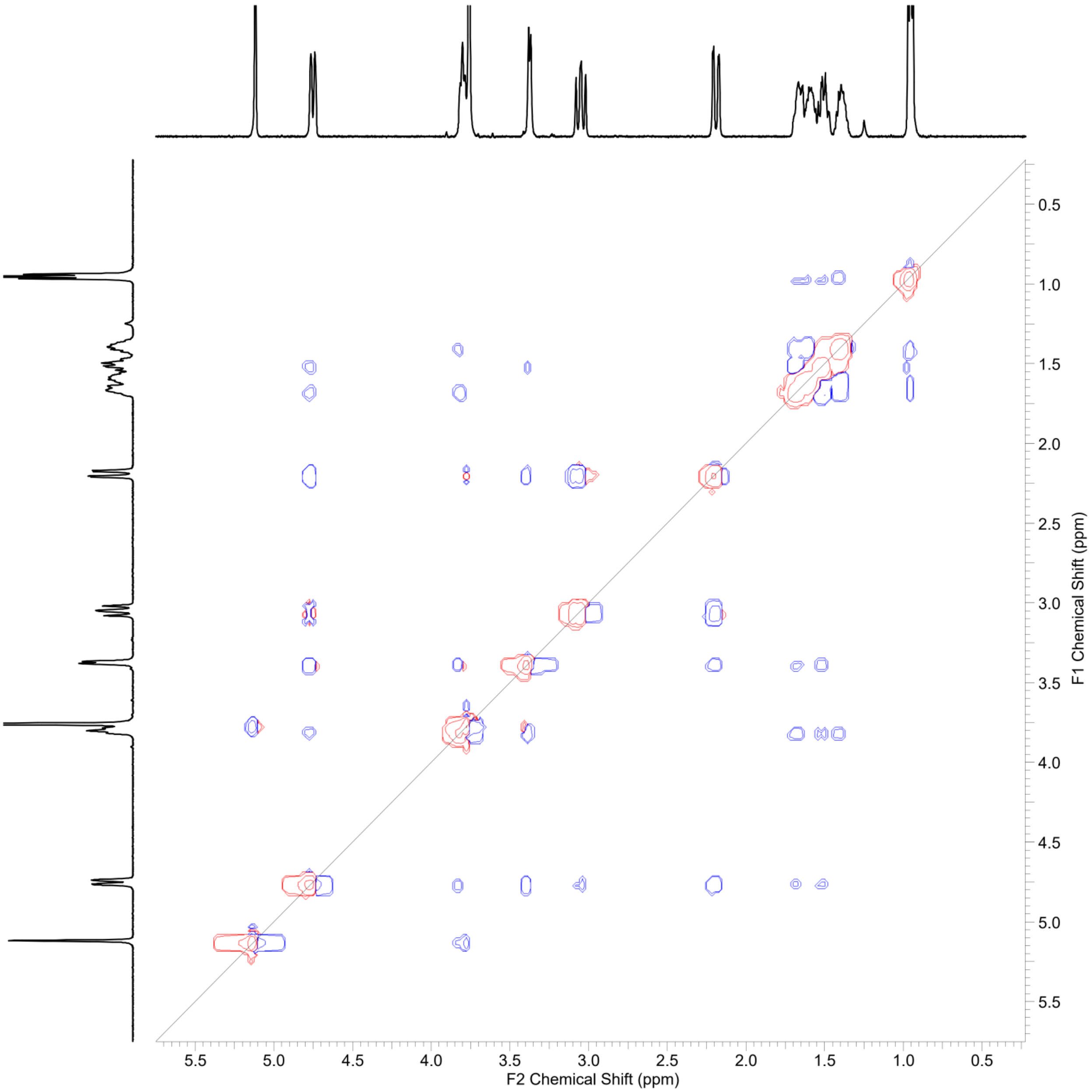

Figure S23: NOESY spectrum ($CDCl_3$) for **2**

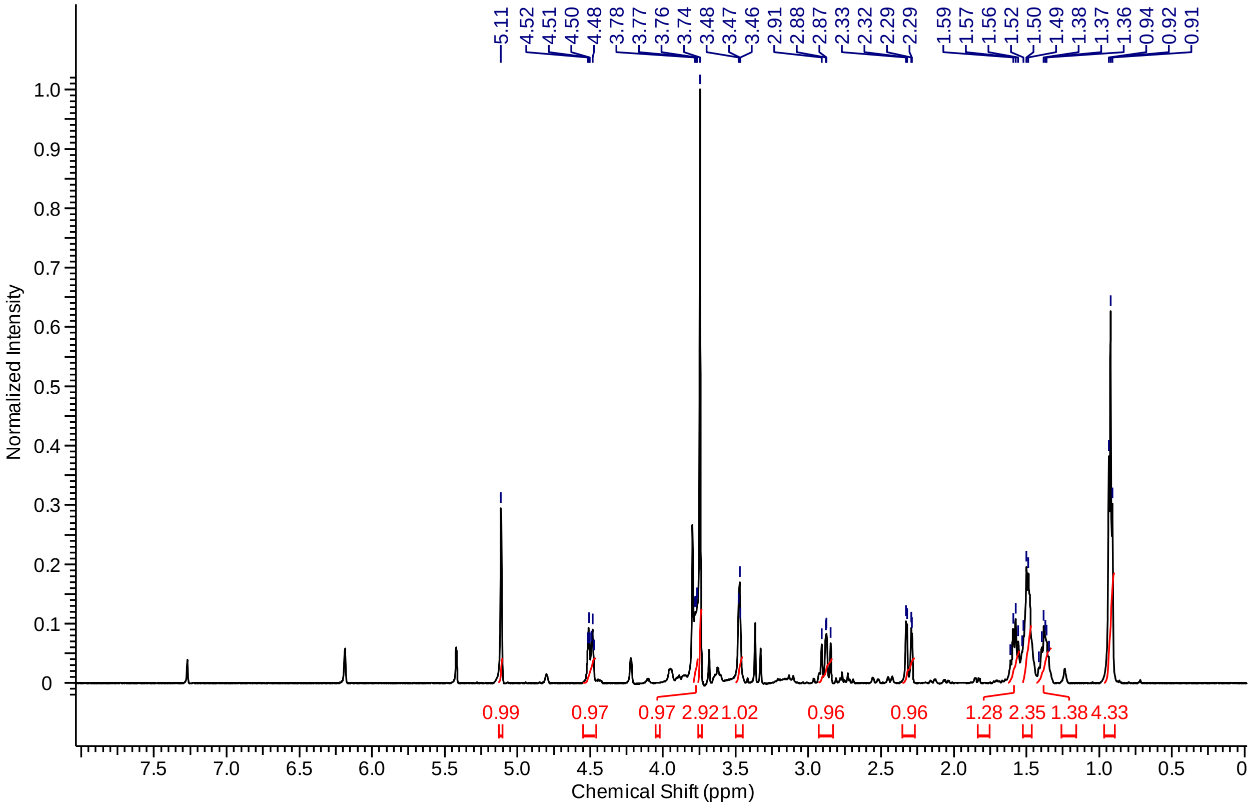

Figure S24: ^{1}H-NMR spectrum (500 MHz, $CDCl_3$) for **3**

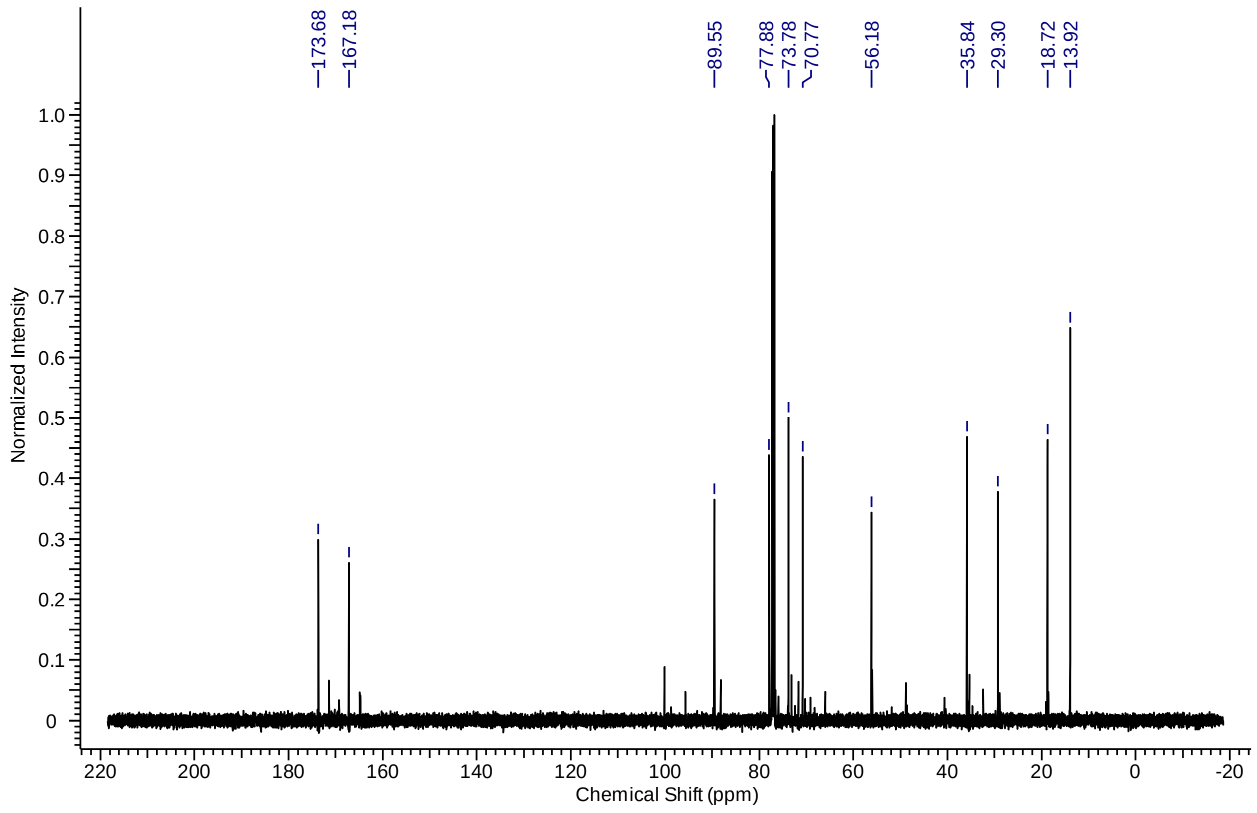

Figure S25: ^{13}C-NMR spectrum (125 MHz, $CDCl_3$) for **3**

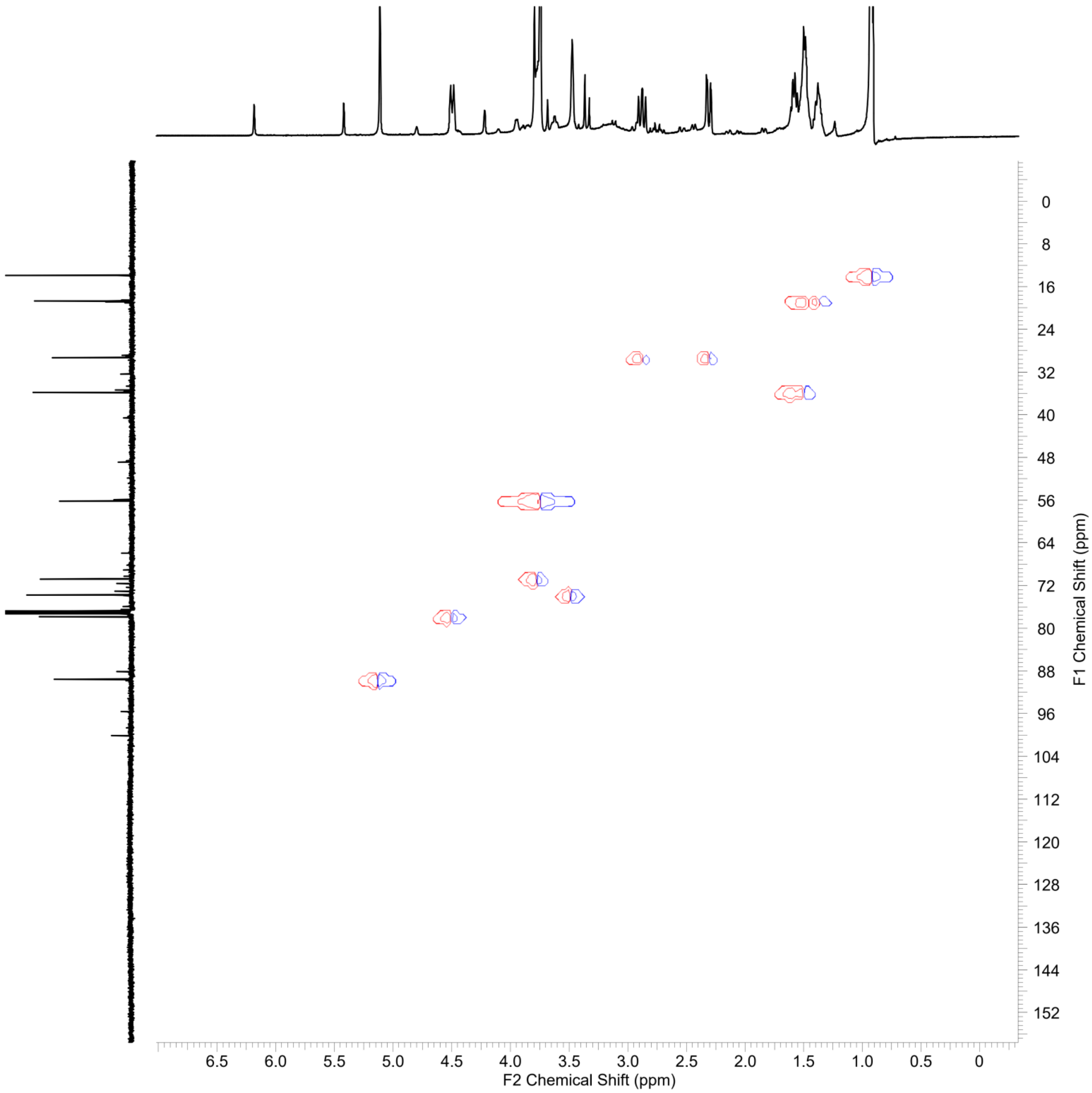

Figure S26: HSQC spectrum ($CDCl_3$) for **3**

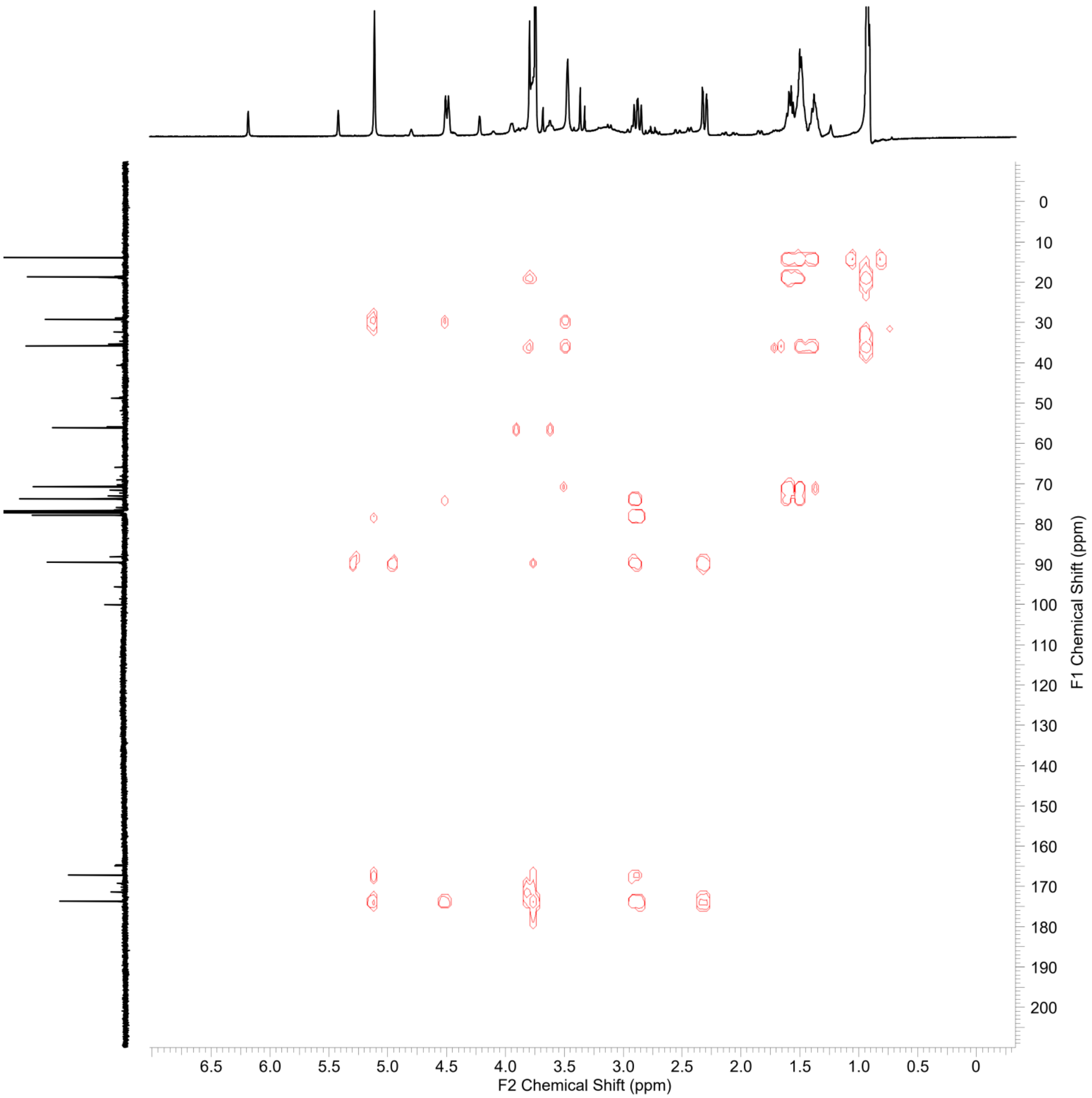

Figure S27: HMBC spectrum ($CDCl_3$) for **3**

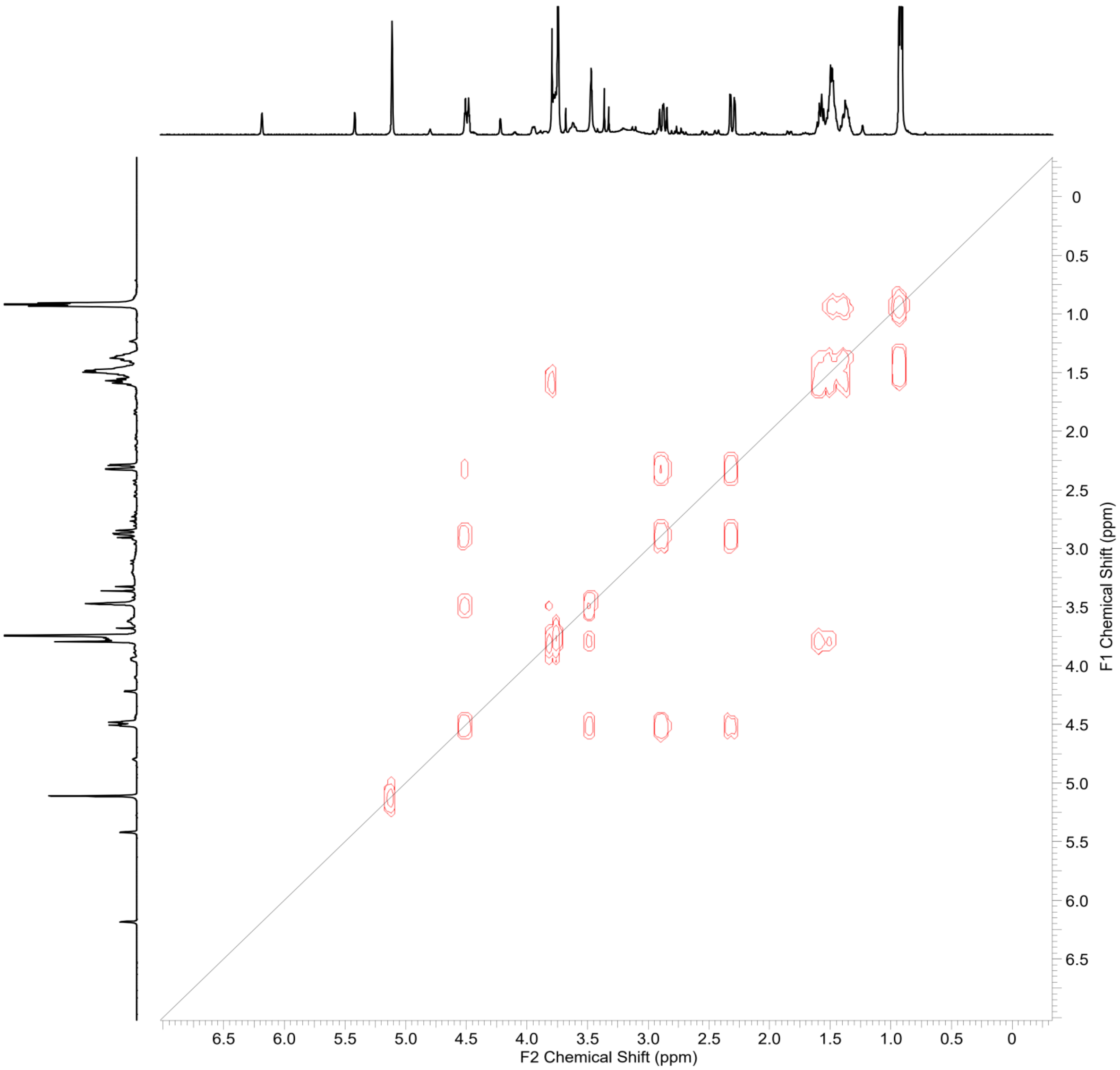

Figure S28: COSY spectrum ($CDCl_3$) for **3**

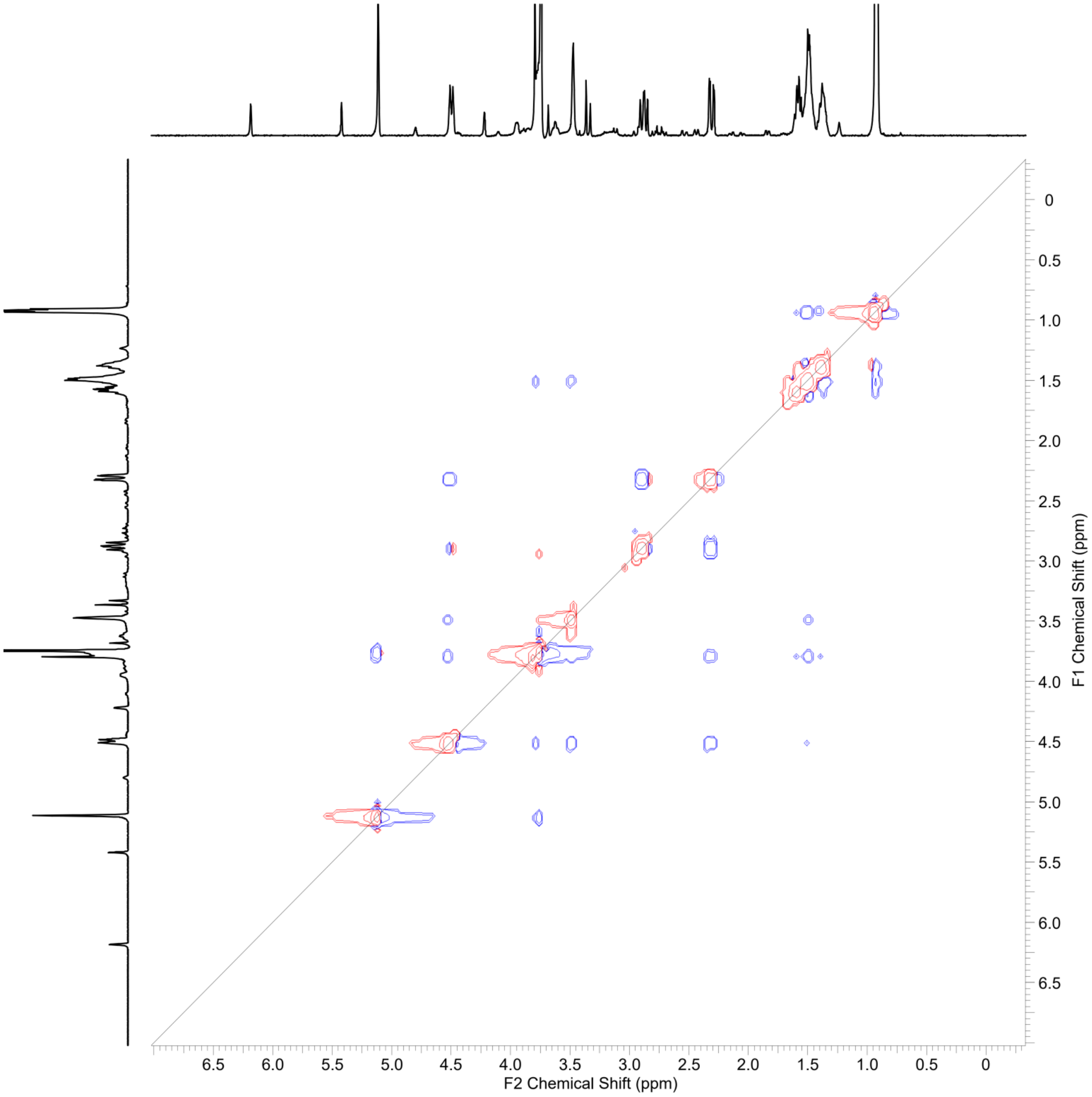

Figure S29: NOESY spectrum ($CDCl_3$) for **3**

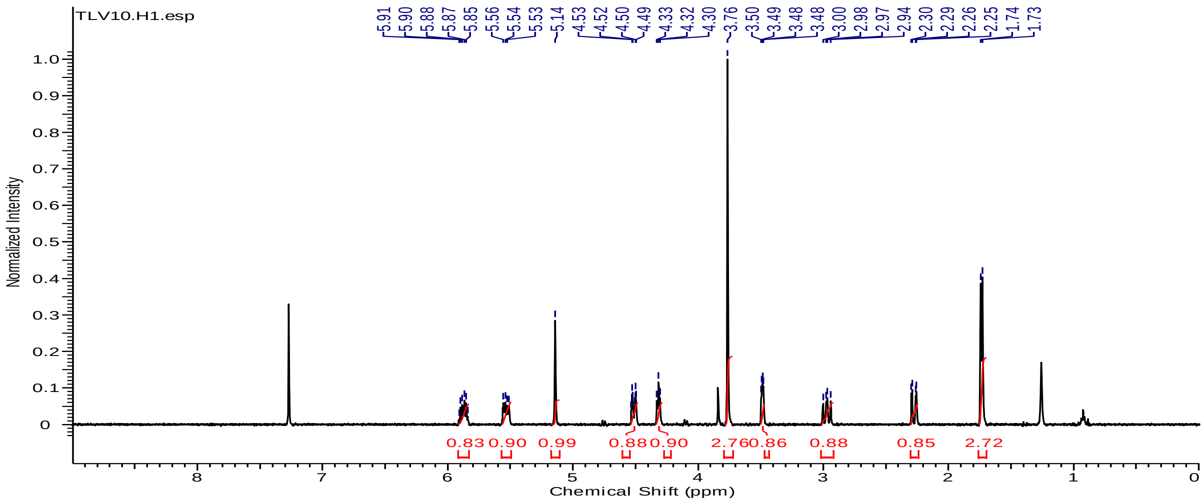

Figure S30: ^{1}H-NMR spectrum (500 MHz, $CDCl_3$) for **4**

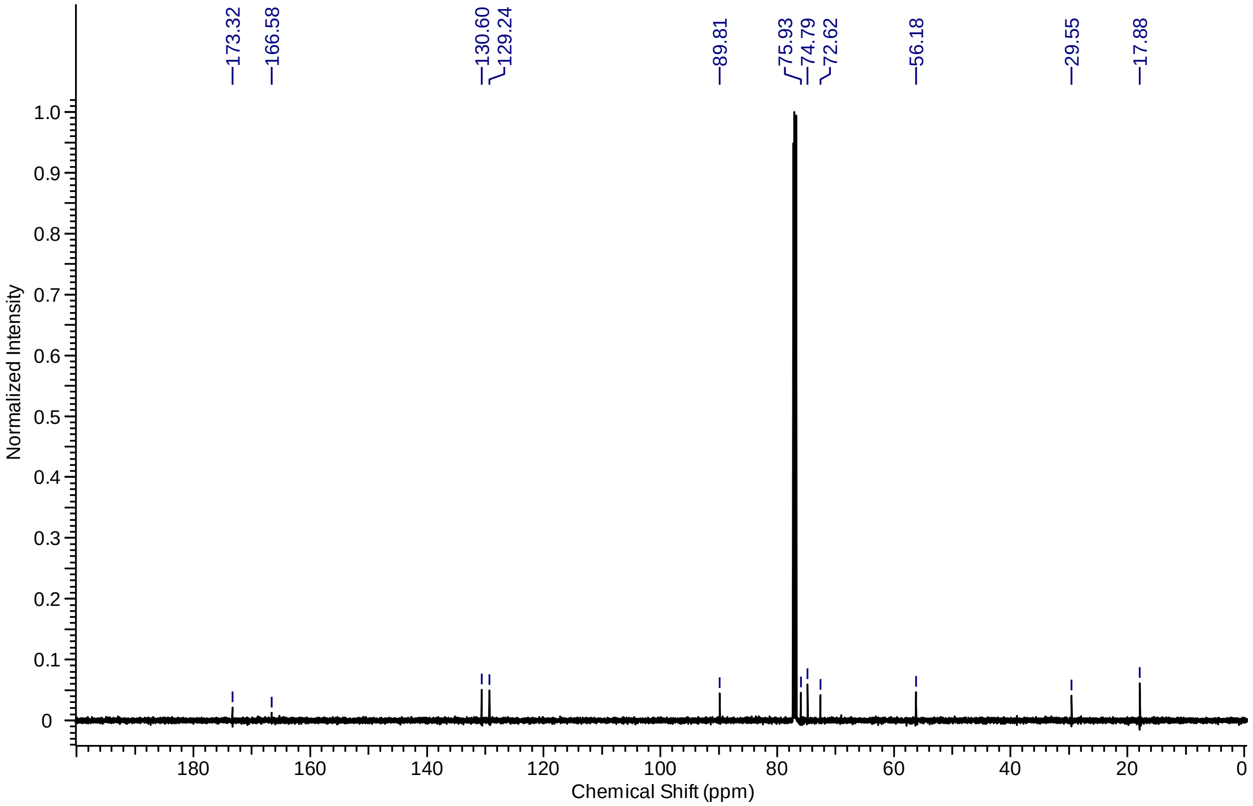

Figure S31: ^{13}C-NMR spectrum (125 MHz, $CDCl_3$) for **4**

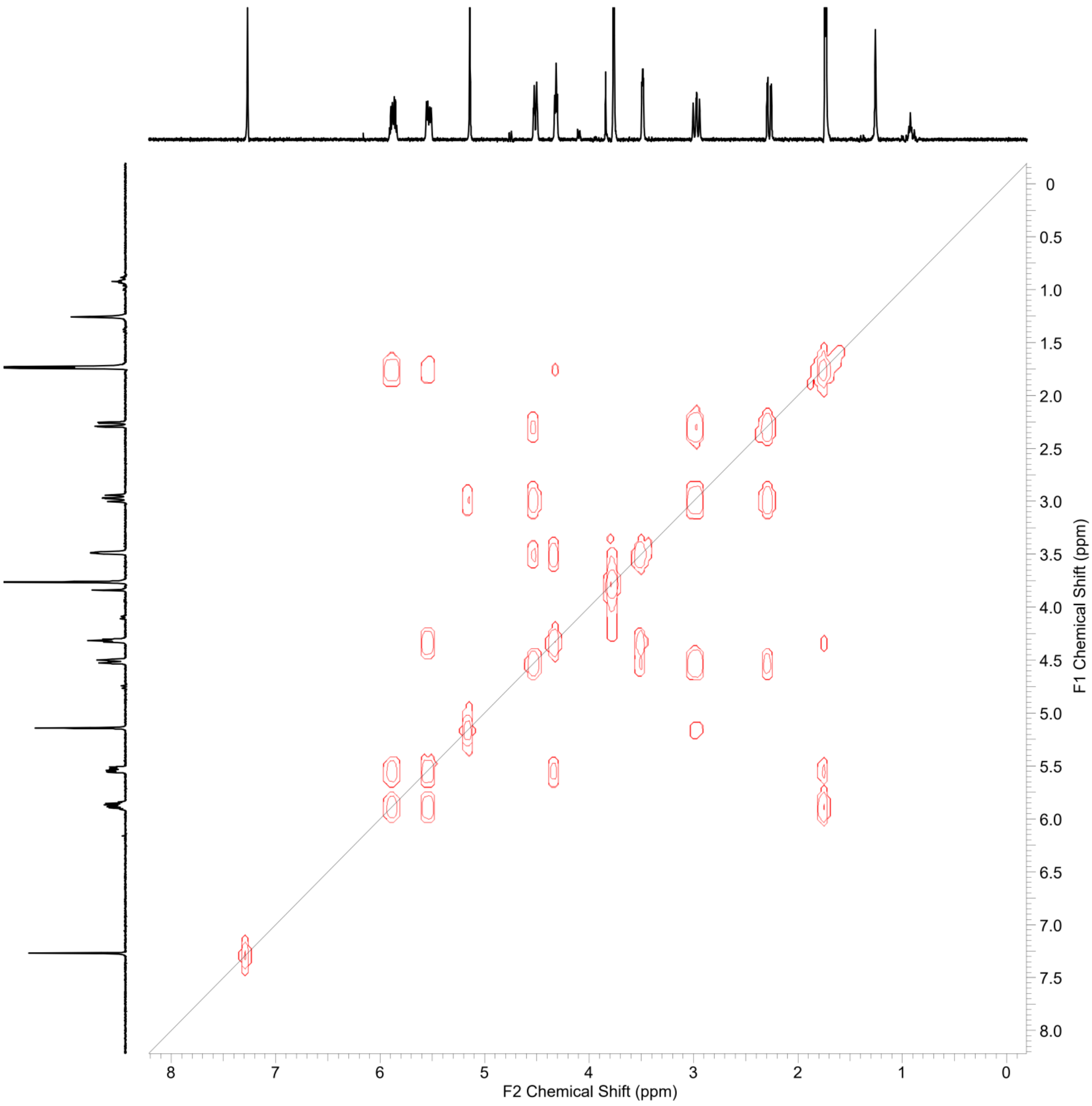

Figure S32: COSY spectrum ($CDCl_3$) for **4**

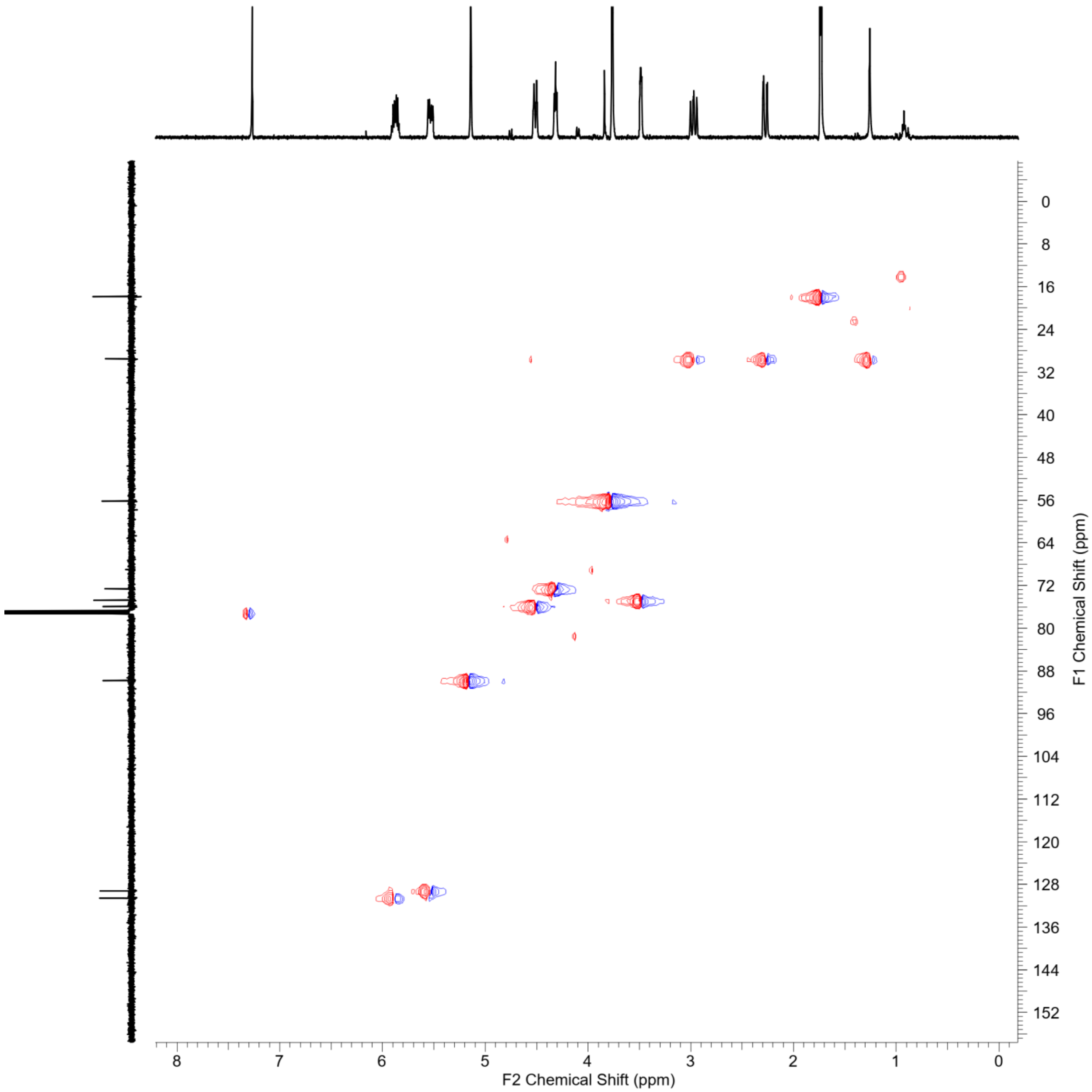

Figure S33: HSQC spectrum ($CDCl_3$) for **4**

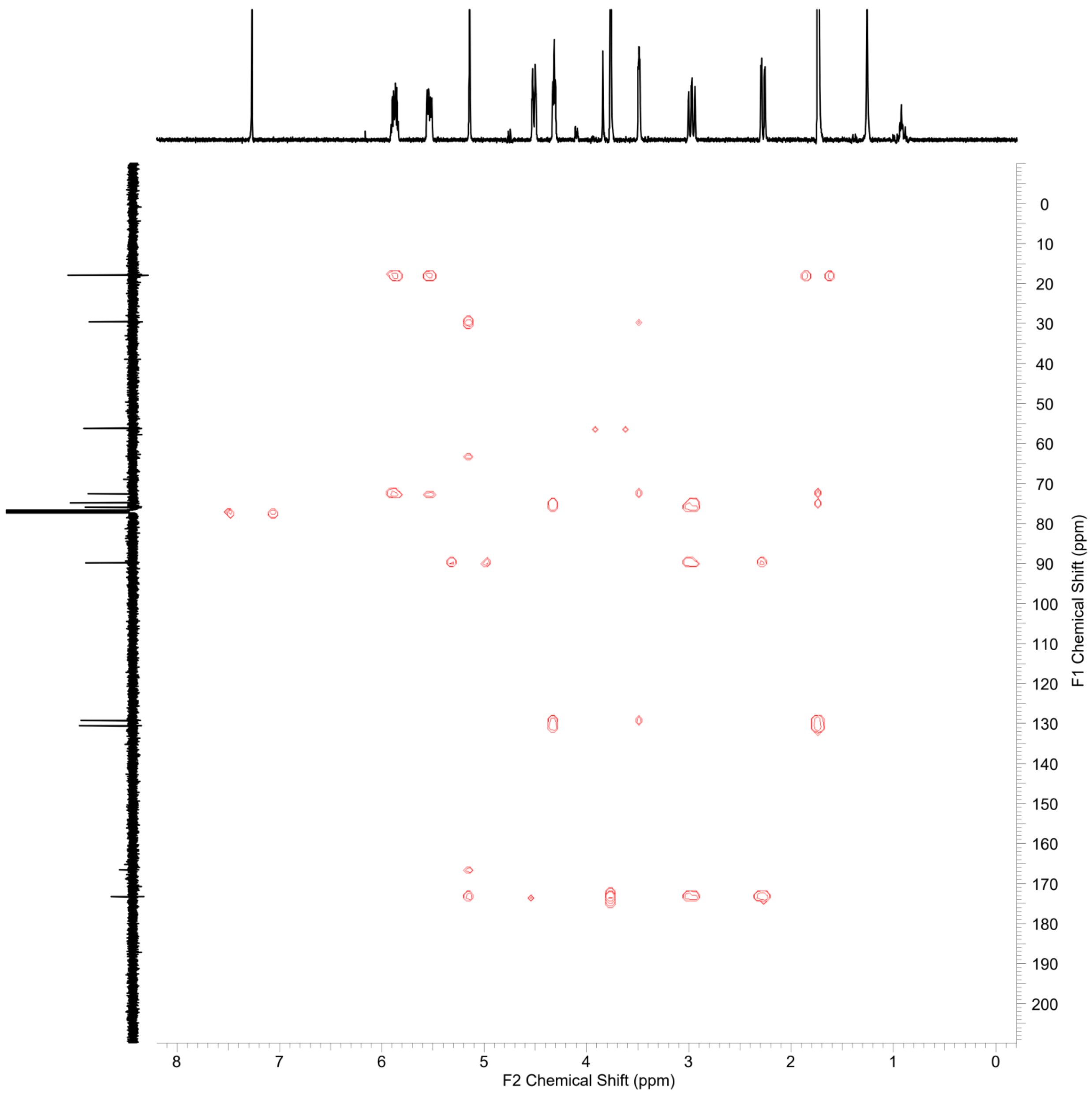

Figure S34: HMBC spectrum ($CDCl_3$) for **4**

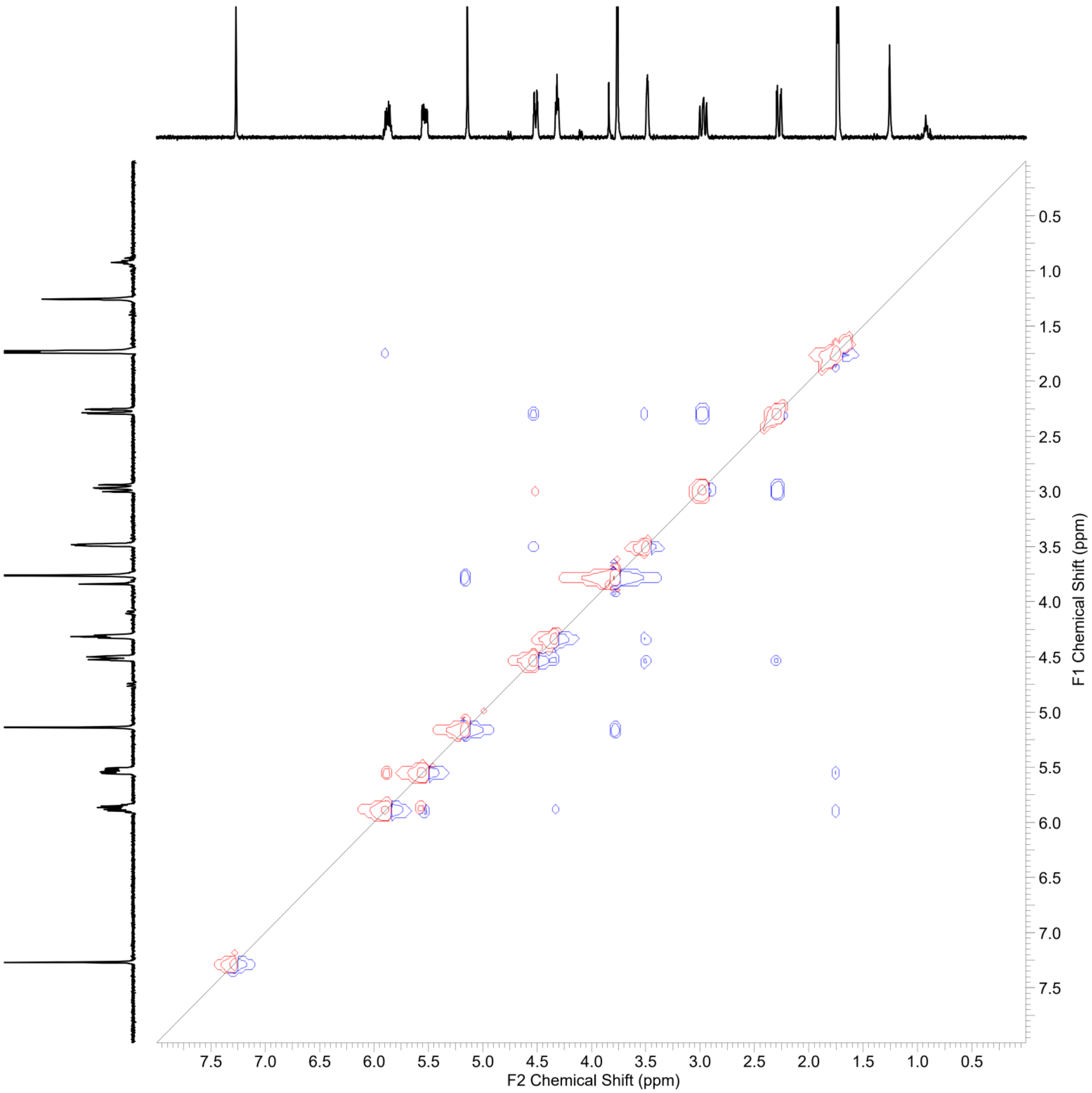

Figure S35: NOESY spectrum ($CDCl_3$) for **4**

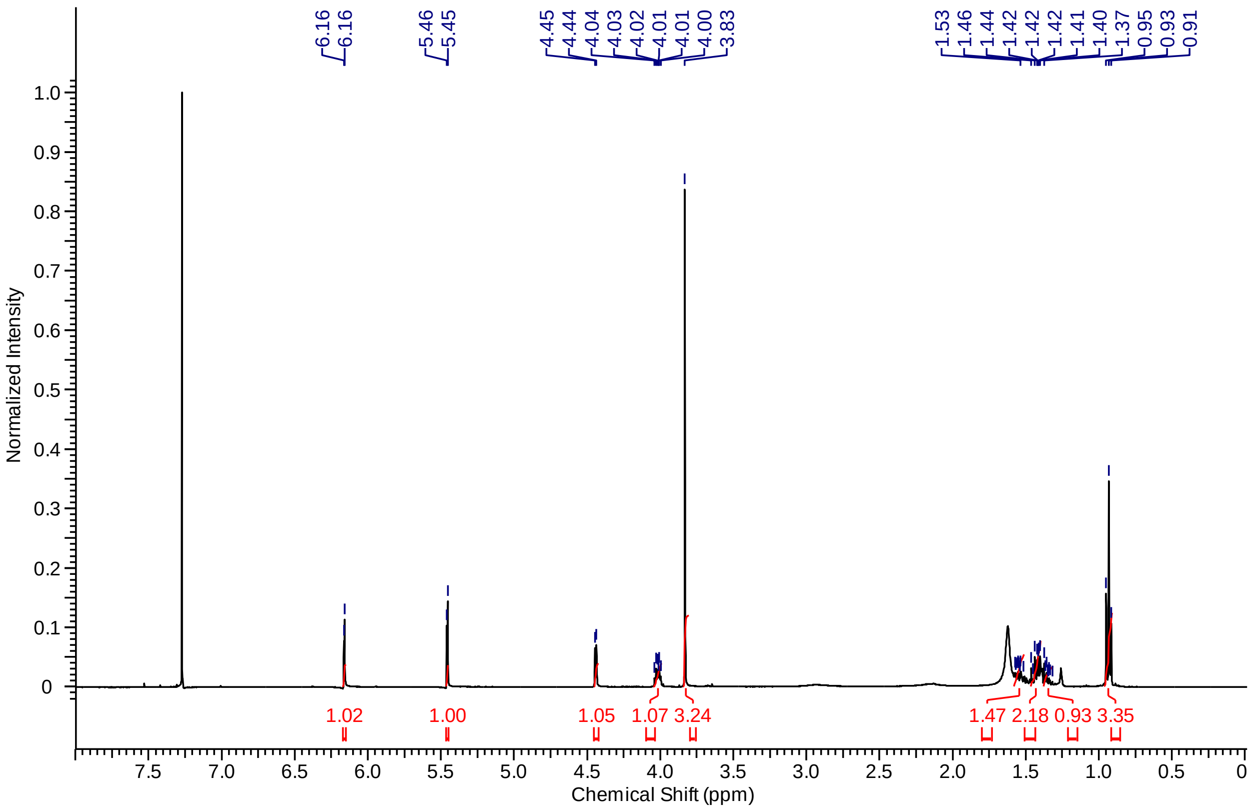

Figure S36: ^{1}H-NMR spectrum (500 MHz, $CDCl_3$) for **5**

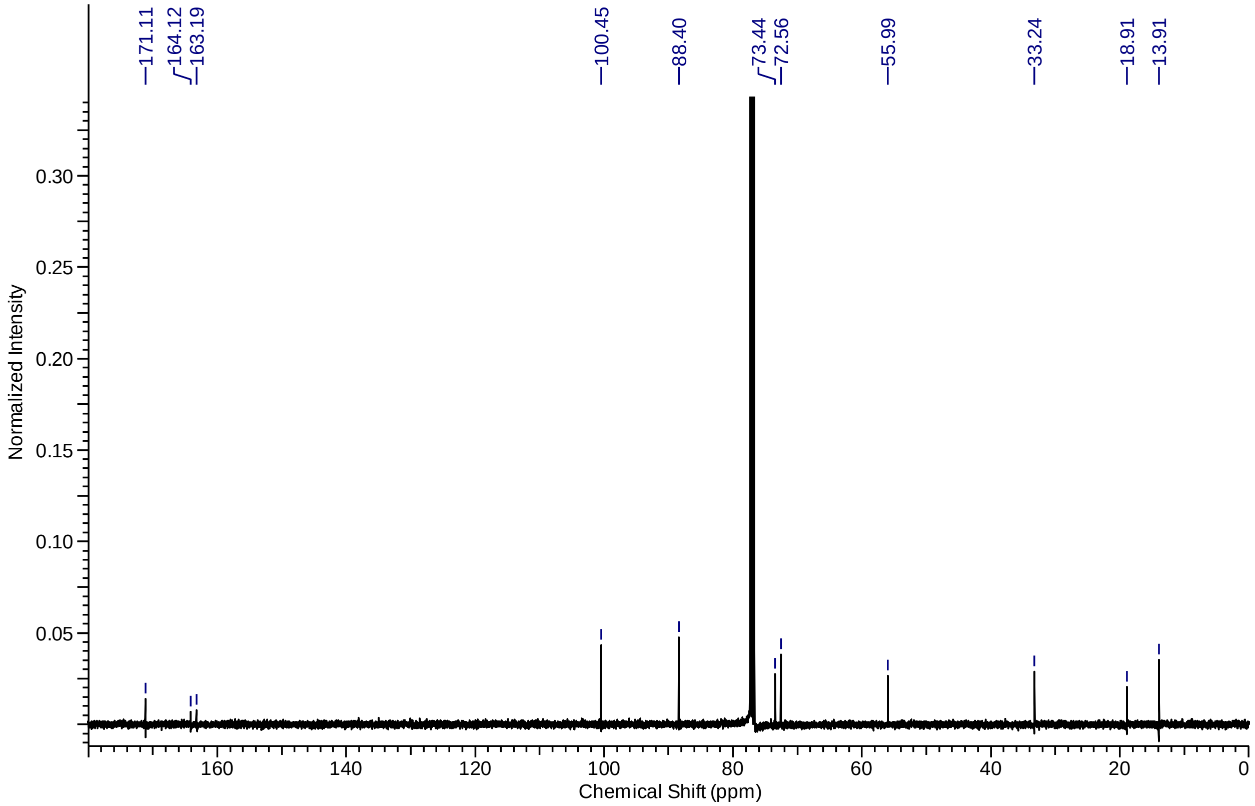

Figure S37: ^{13}C-NMR spectrum (125 MHz, $CDCl_3$) for **5**

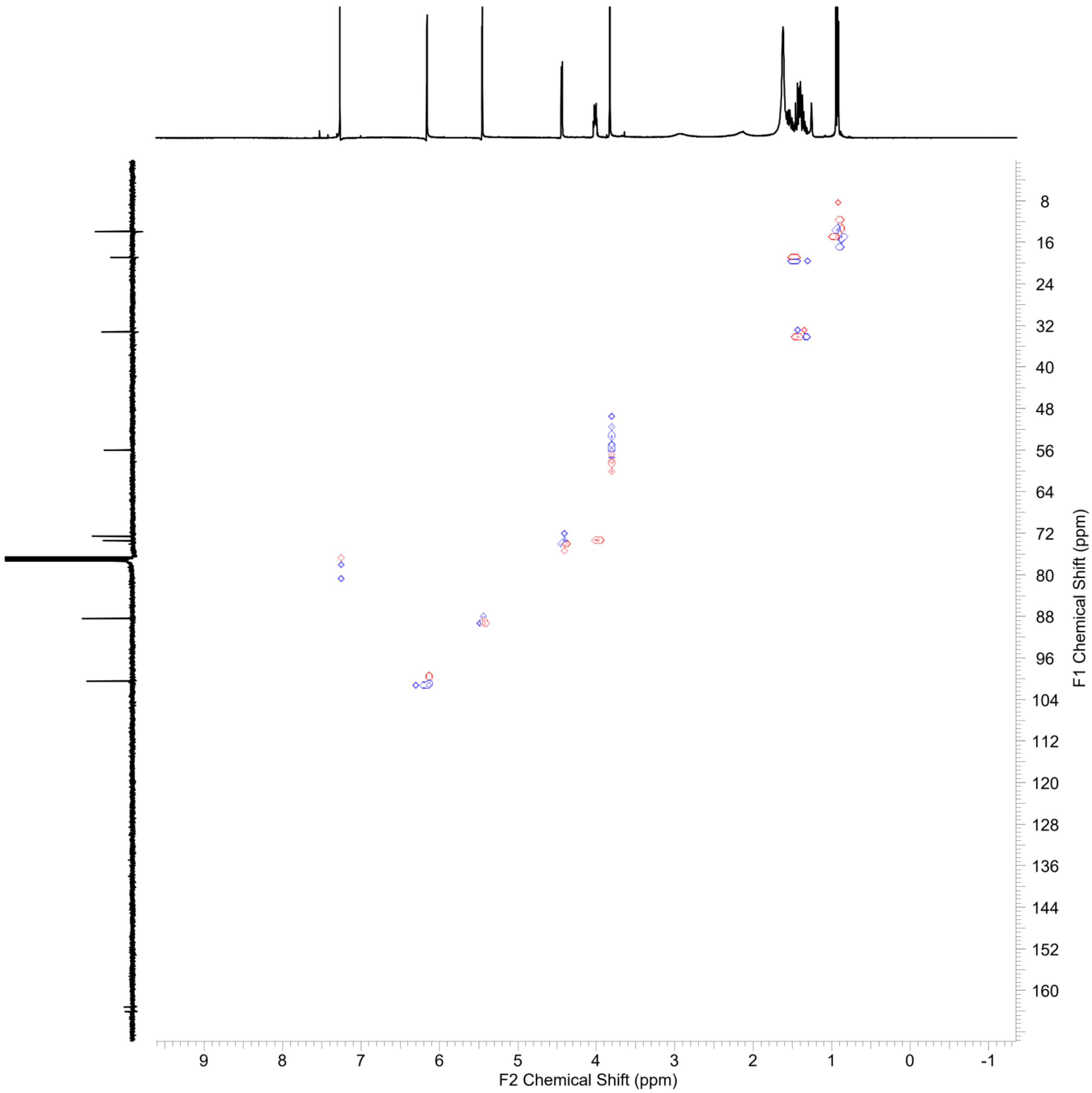

Figure S38: HSQC spectrum ($CDCl_3$) for **5**

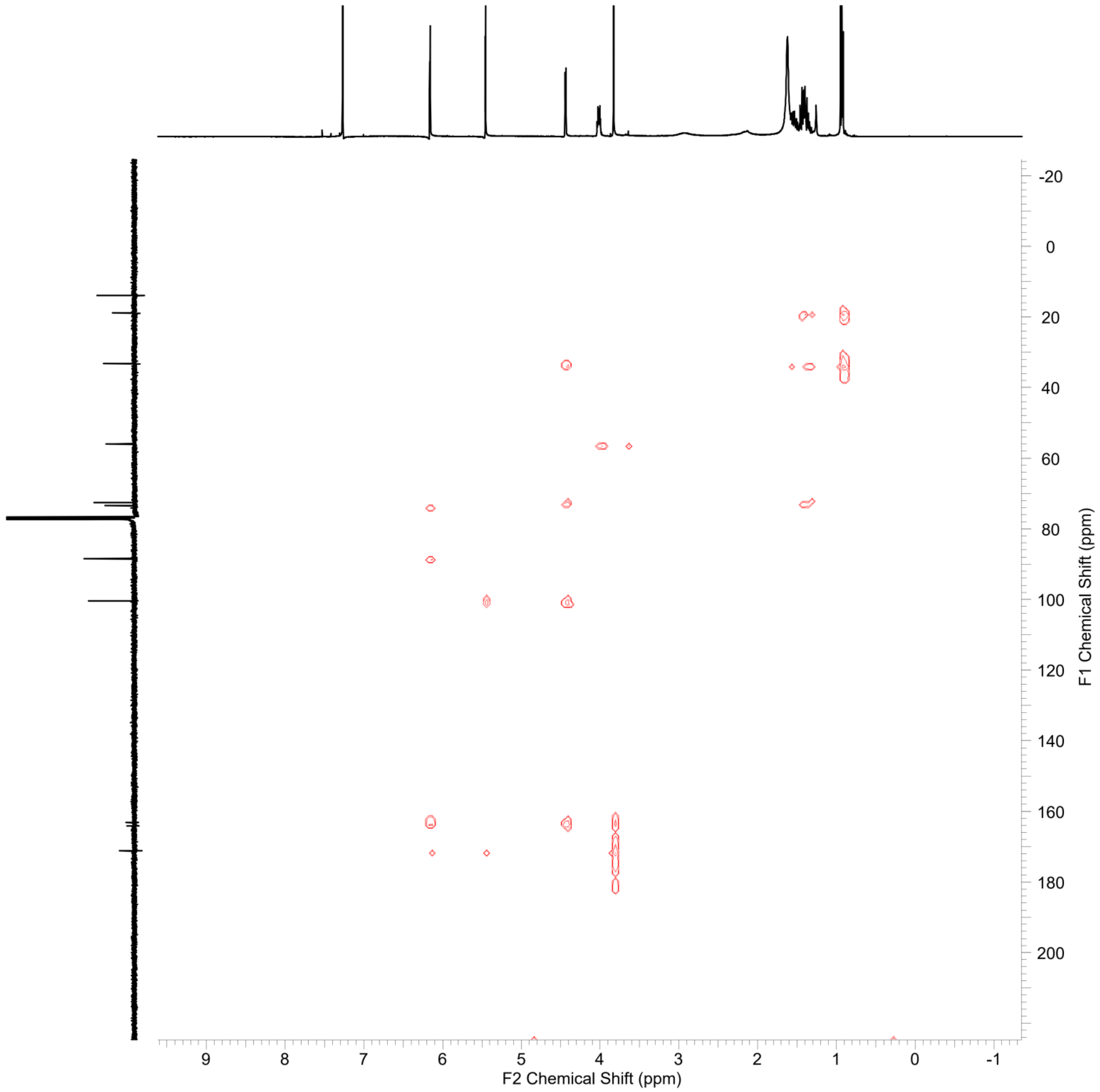

Figure S39: HMBC spectrum ($CDCl_3$) for **5**

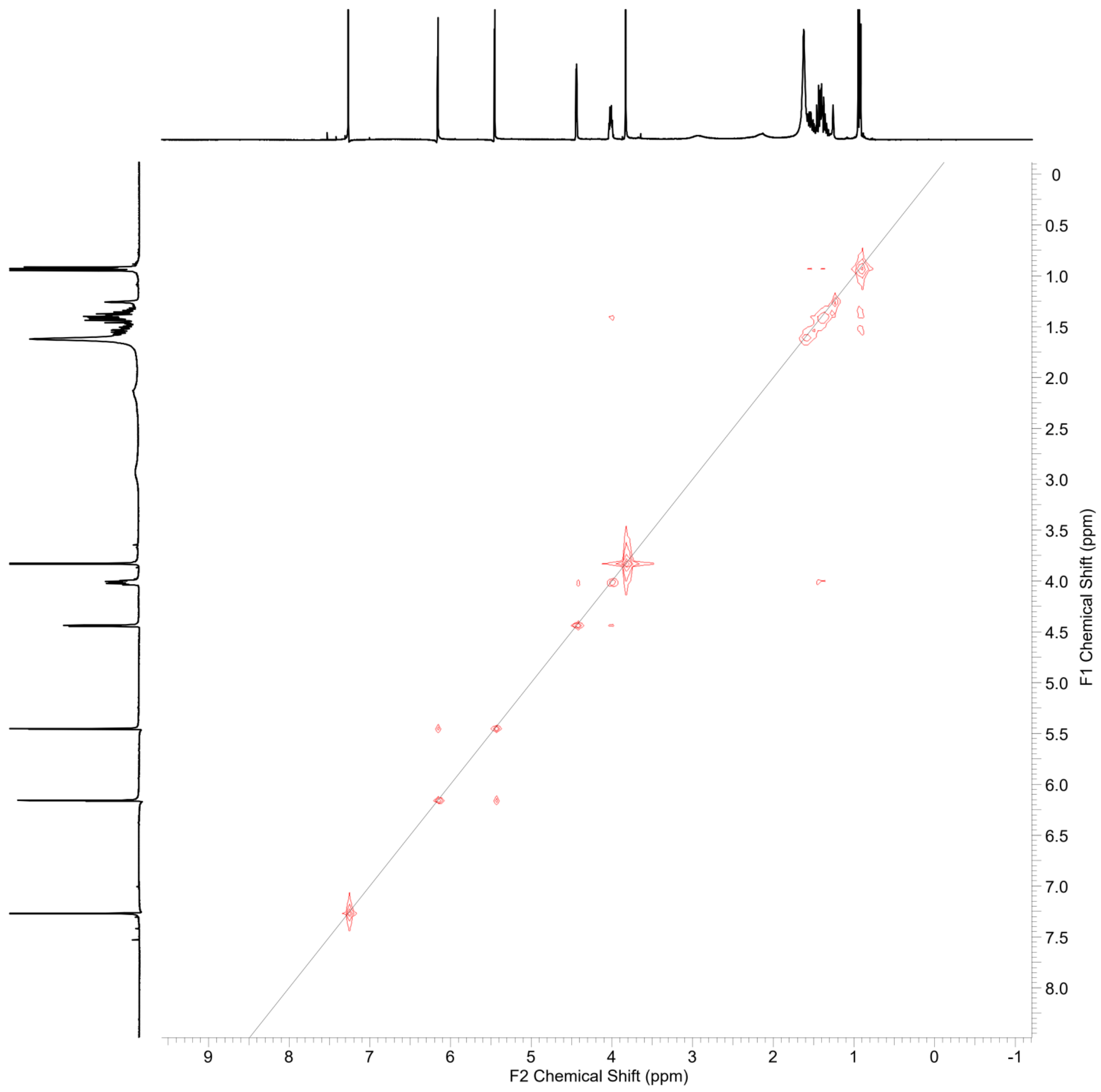

Figure S40: COSY spectrum ($CDCl_3$) for **5**

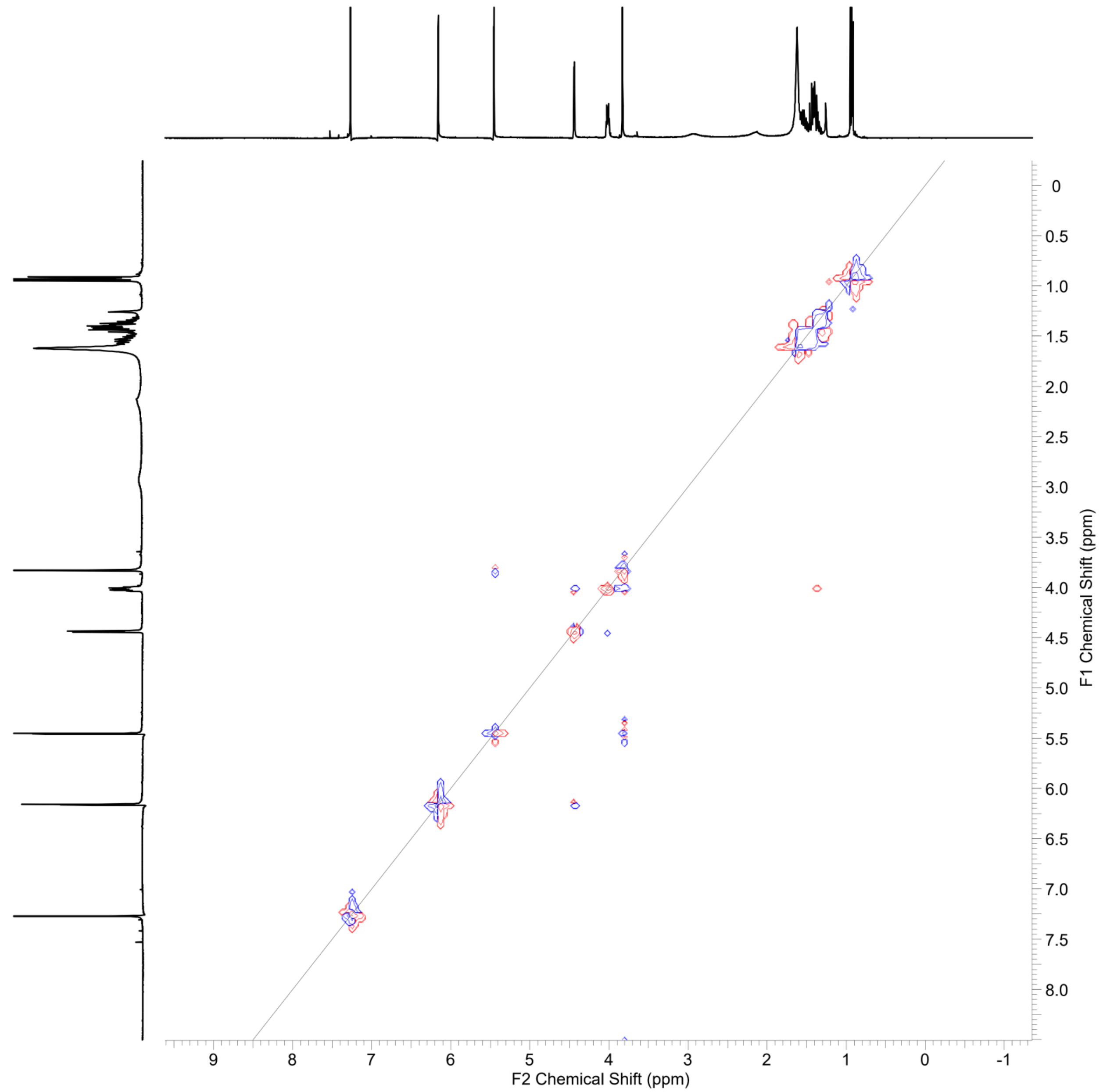

Figure S41: NOESY spectrum ($CDCl_3$) for **5**

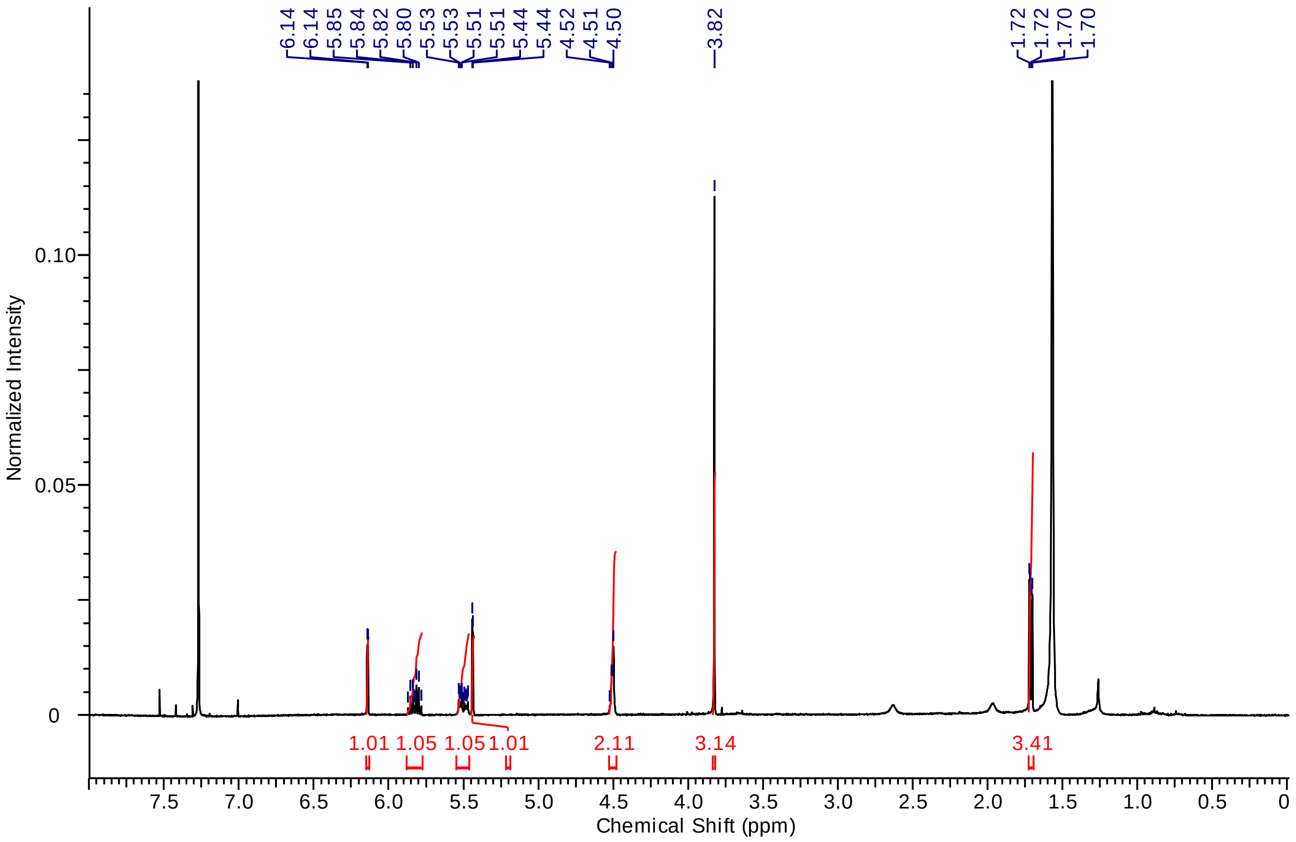

Figure S42: ^{1}H-NMR spectrum (500 MHz, $CDCl_3$) for **6**

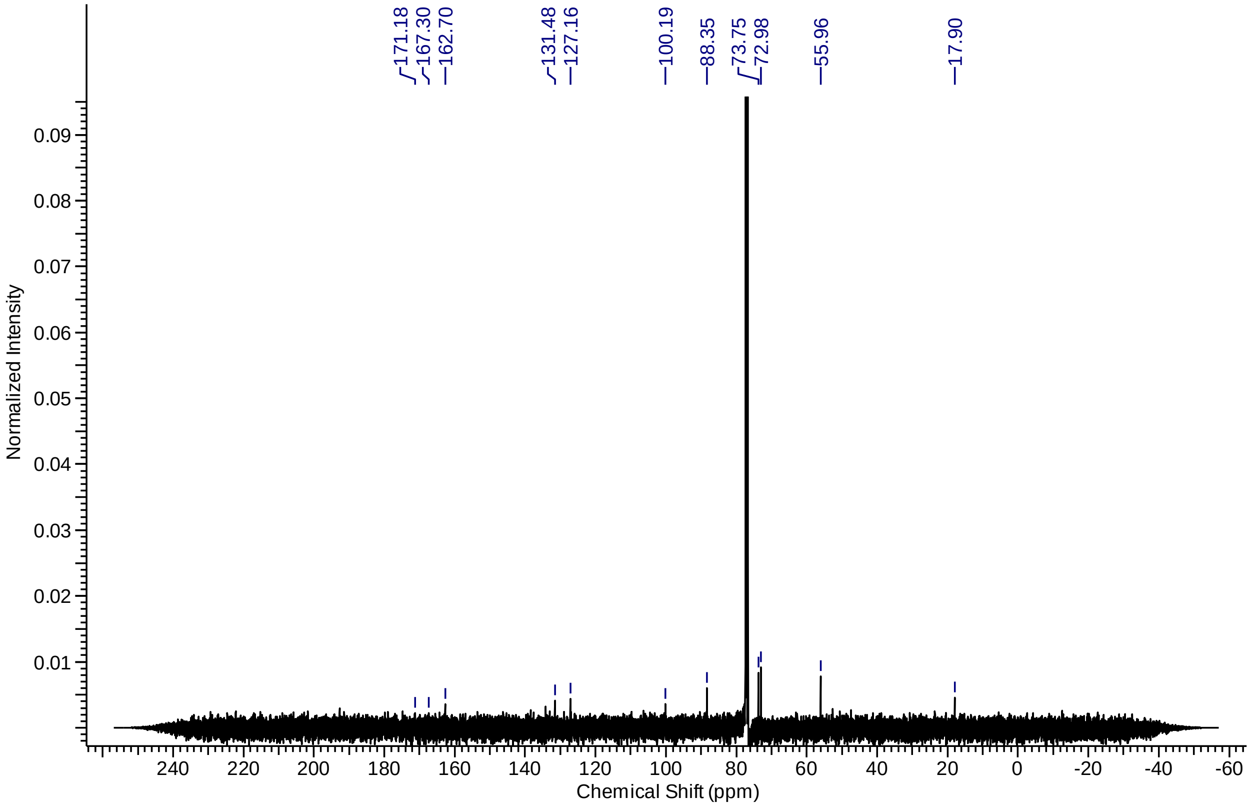

Figure S43: ^{13}C-NMR spectrum (125 MHz, $CDCl_3$) for 6

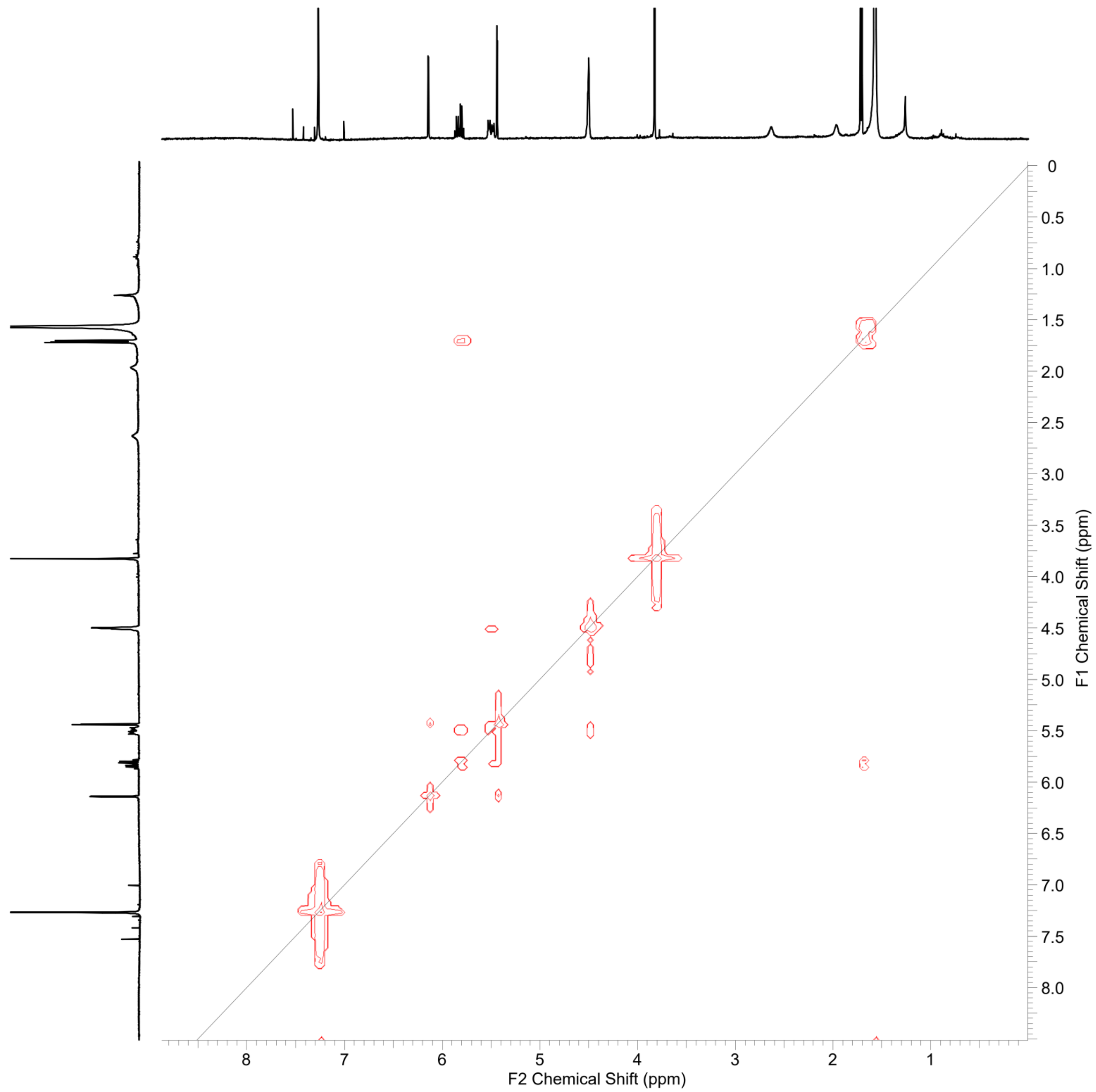

Figure S44: COSY spectrum ($CDCl_3$) for **6**

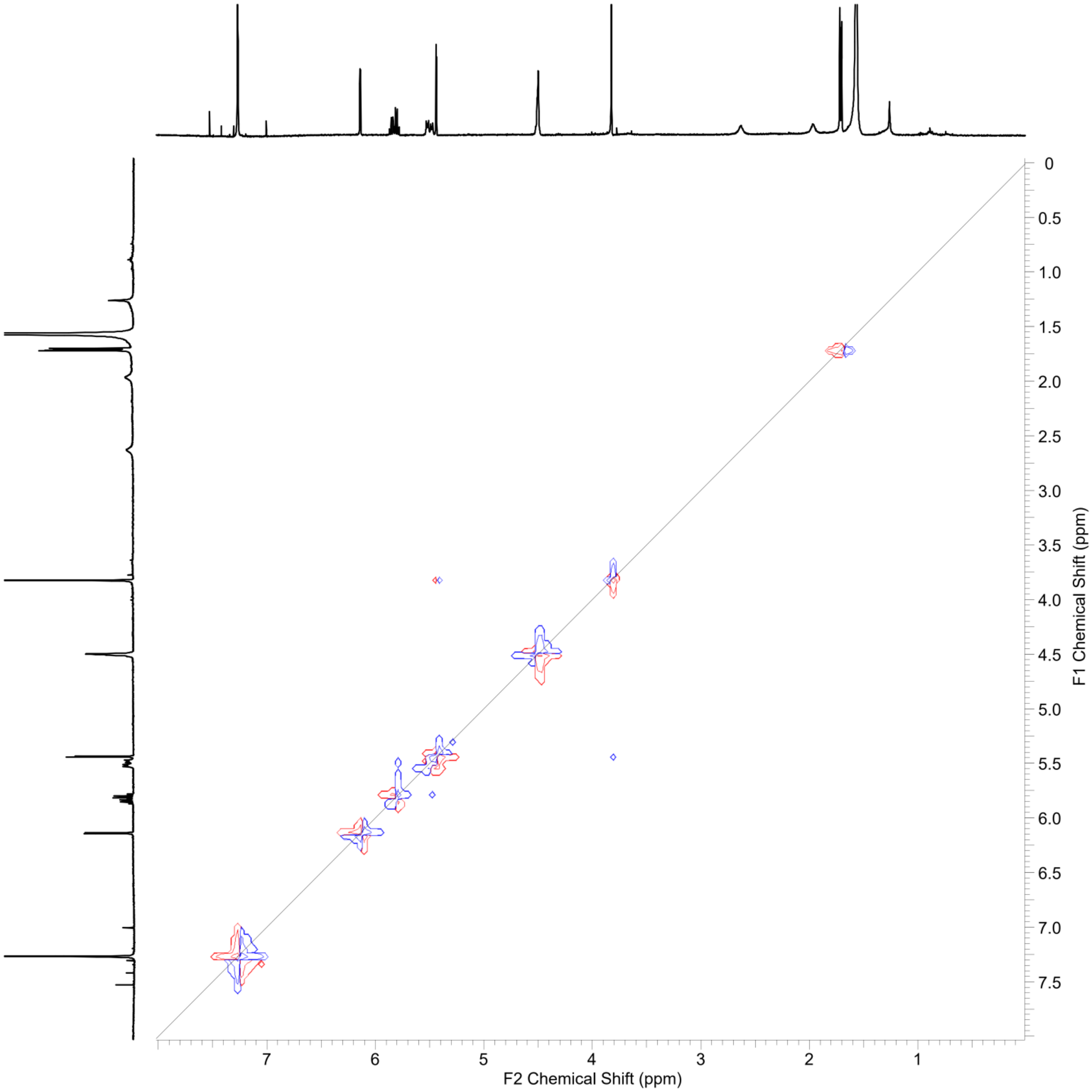

Figure S45: NOESY spectrum ($CDCl_3$) for **6**

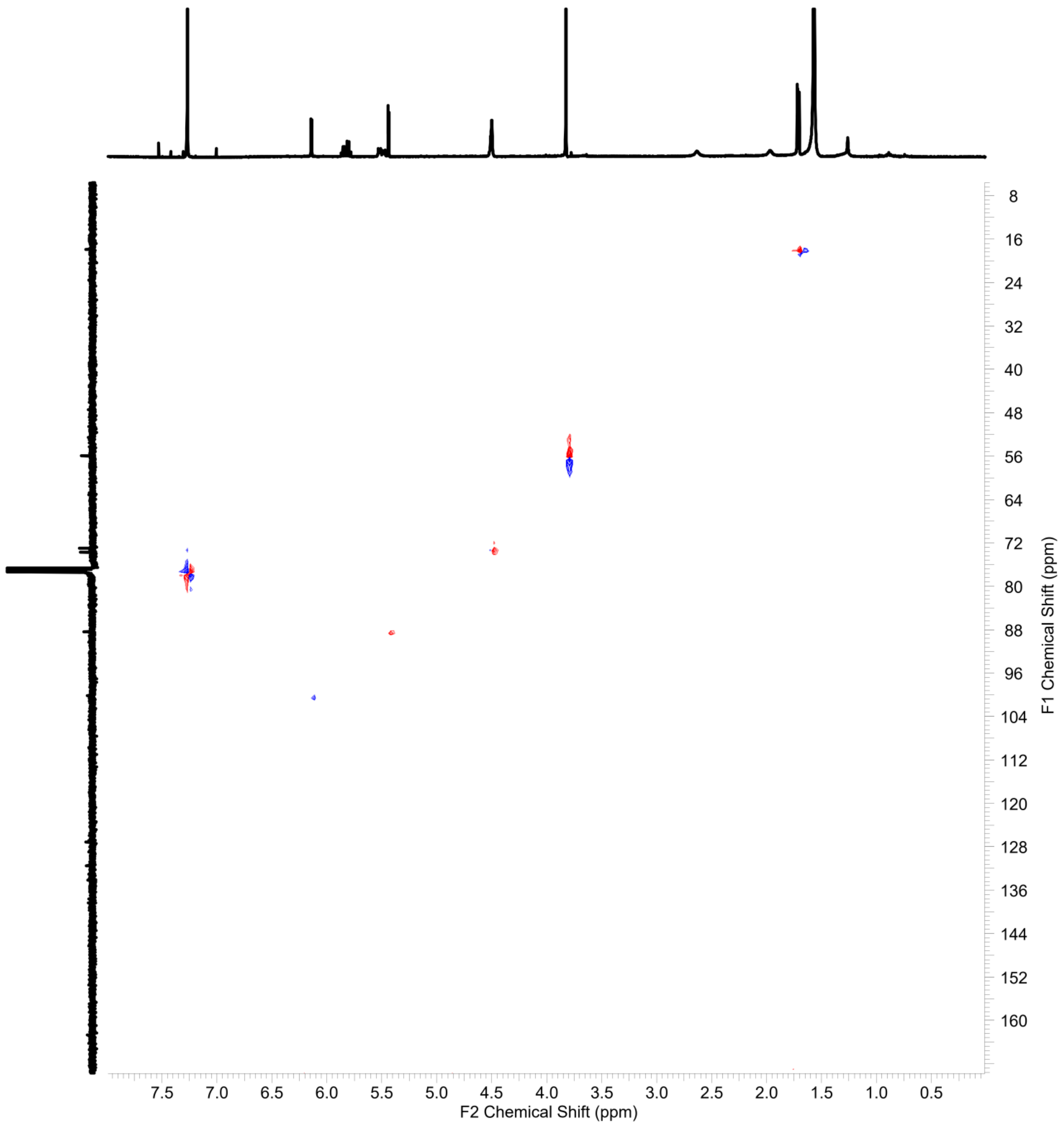

Figure S46: HSQC spectrum ($CDCl_3$) for **6**

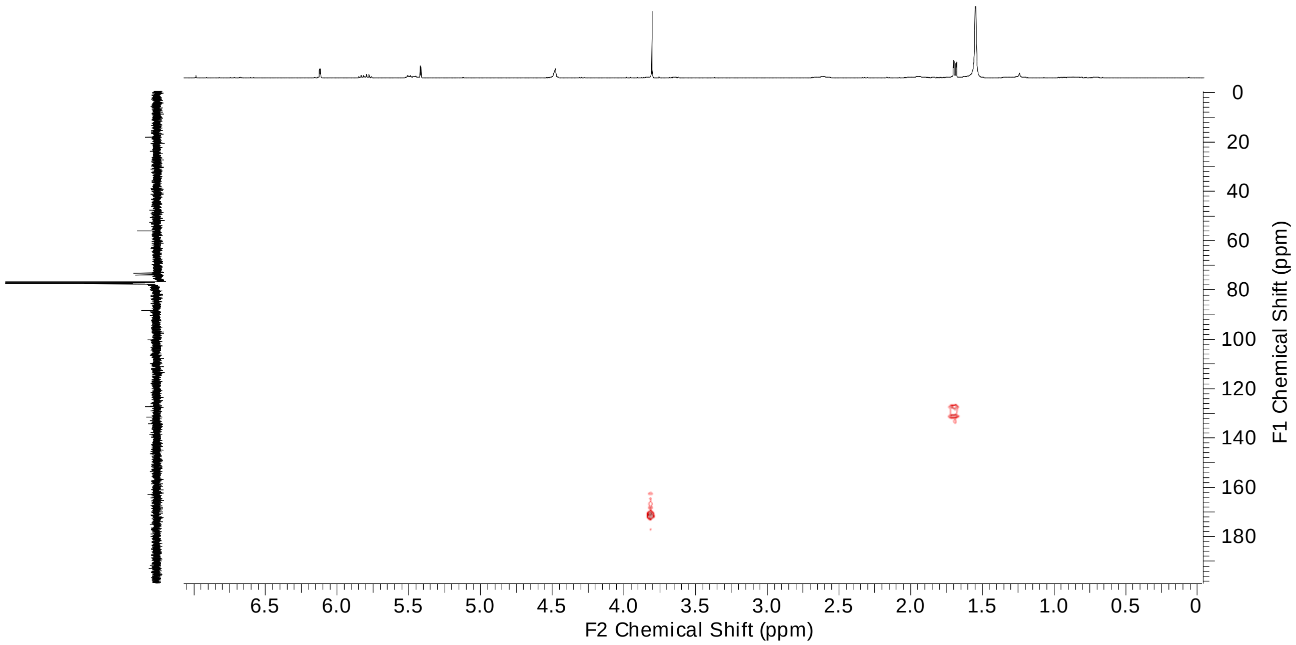

Figure S47: HMBC spectrum (CDCl3) for **6**

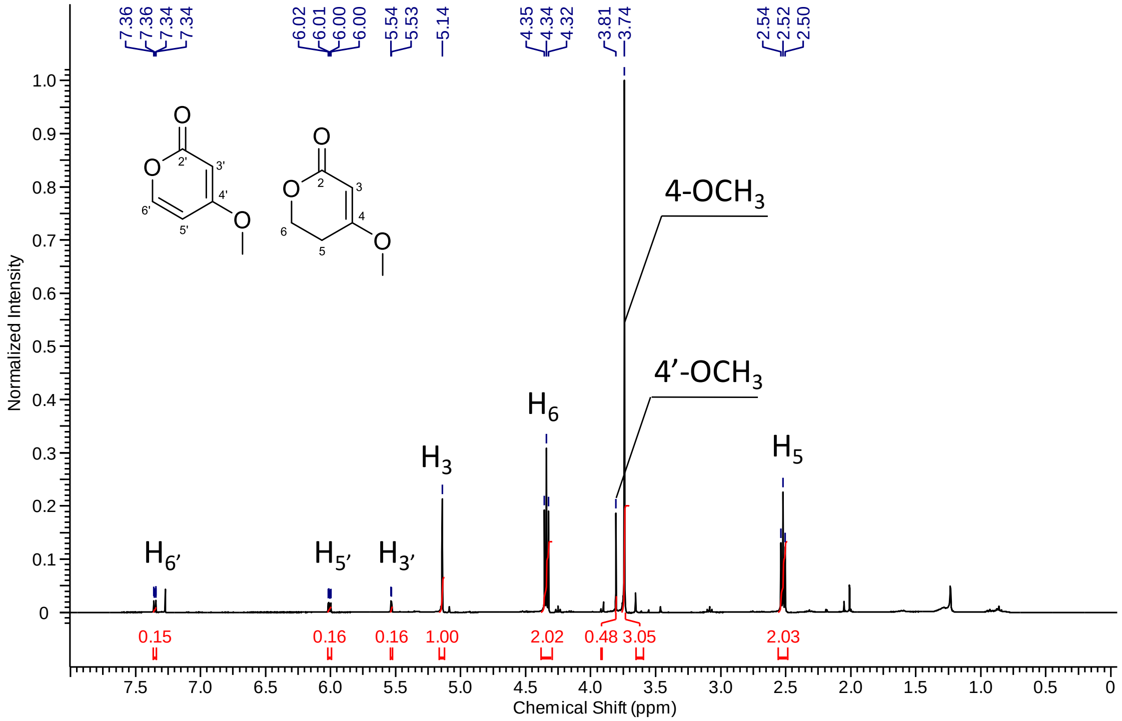

Figure S48: ^{1}H-NMR spectrum (500 MHz, CDCl3) for **7-8**

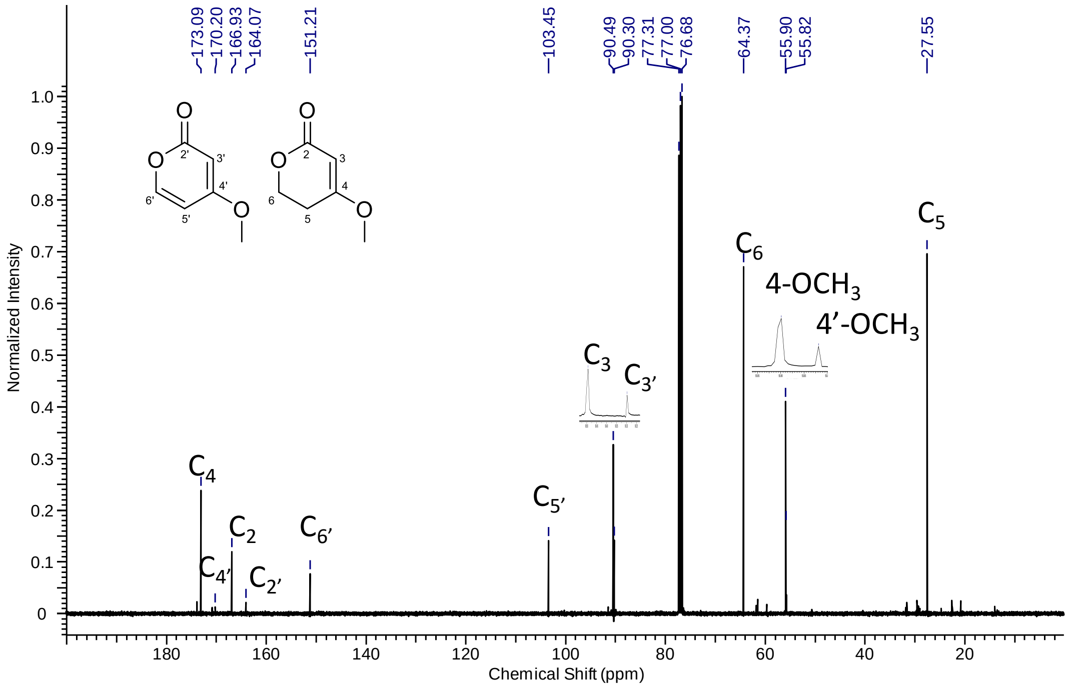

Figure S49: ^{13}C-NMR spectrum (125 MHz, CDCl3) for **7-8**

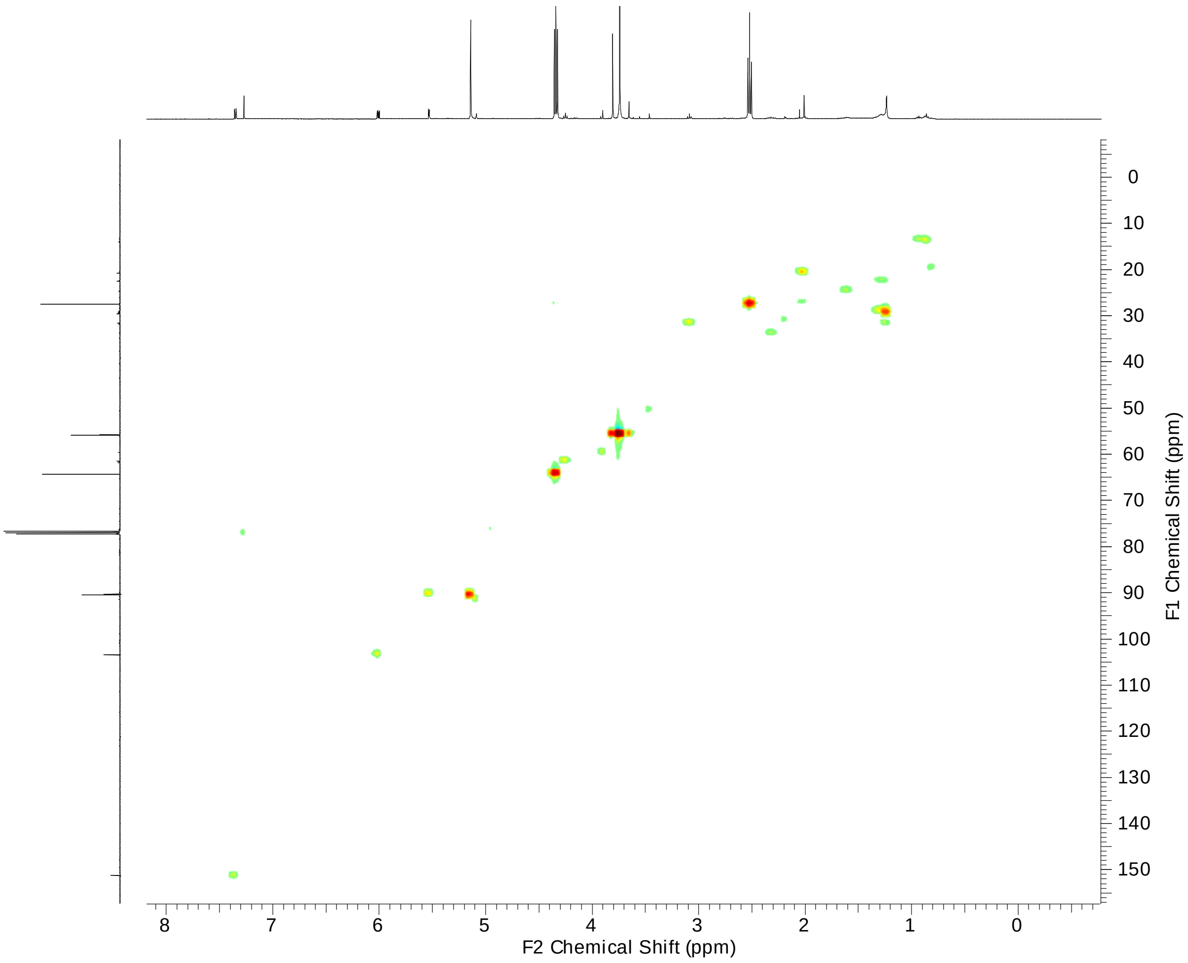

Figure S50: HSQC spectrum ($CDCl_3$) for **7-8**

References

1. Isaac, B.G.; Ayer, S.W.; Stonard, R.J. Arabenoic acid, a natural product herbicide of fungal origin. *J. Antibiot.* **1991**, *44*, 793–794.
2. Larsen, T.O.; Frisvad, J.C.; Christophersen, C. Arabenoic acid (verrucolone), a major chemical indicator of *Penicillium verrucosum*. *Biochem. Syst. Ecol.* **1998**, *26*, 463–465.
3. Gorst-Allman, C.P.; Steyn, P.S. Biosynthesis of 5,6-dihydro-4-methoxy-2*H*-pyran-2-one in *Penicillium italicum*. *S. Afr. j. chem.* **1983**, *36*, 83–84.
4. Kimura, Y.; Tamura, S. Isolation and structure of Pestalotin, a gibberellin synergist from *Pestalotia cryptomeriaecola*. *Agric. Biol. Chem.* **1972**, *36*, 1925–1930.
5. Ellestad, G.A.; McGahren, W.J.; Kunstmann, M.P. Structure of a new fungal lactone, LL-P880α, from an unidentified *Penicillium* sp. *J. Org. Chem.* **1972**, *37*, 2045–2047.
6. Kimura, Y.; Hamasaki, T.; Nakajima, H. Stereochemistry and biological activities of LL-P880γ, a pestalotin analogue, produced by *Penicillium citreo-viride*. *Agric. Biol. Chem.* **1986**, *50*, 1649–1650.
7. Sakurai, I.; Shimizu, S.; Yamamoto, Y. Studies on metabolites of *Macrophoma commelinae*. III. : Isolation of new metabolites and biosynthesis of macommelin group. *Chem. Pharm. Bull.* **1988**, *36*, 1328–1335.
8. Evidente, A.; Rodeva, R.; Andolfi, A.; Stoyanova, Z.; Perrone, C.; Motta, A. Phytotoxic polyketides produced by *Phomopsis foeniculi*, a strain isolated from diseased Bulgarian fennel. *Eur J Plant Pathol* **2011**, *130*, 173–182.
9. Ayer, W.A.; Trifonov, L.S.; Hutchison, L.J.; Chakravarty, P. Metabolites from a wood-Inhabiting cup fungus, *Urnula craterium*. *Nat. Prod. Lett.* **2000**, *14*, 405–410.
10. Kimura, Y.; McGahren, W.J.; Suzuki, A.; Tamura, S. Structure of a New Fungal Pyrone, from an Unidentified *Penicillium* sp. *Agric. Biol. Chem.* **1978**, *42*, 1625–1626.
11. Nose, H.; Seki, A.; Yaguchi, T.; Hosoya, A.; Sasaki, T.; Hoshiko, S.; Shomura, T. PF1163A and B, new antifungal antibiotics produced by *Penicillium* sp. *J. Antibiot.* **2000**, *53*, 33–37.
12. Sakai, K.; Chiba, H.; Kaneto, R.; Sakamoto, M.; Okamura, K.; Tone, H. Mer-NF8054A and X, Novel antifungal steroids, isolated from *Aspergillus* sp. *J. Antibiot.* **1994**, *47*, 591–594.
13. Zhou, G.-X.; Wijeratne, E.M.K.; Bigelow, D.; Pierson, L.S.; VanEtten, H.D.; Gunatilaka, A.A.L. Aspochalasins I, J, and K: Three new cytotoxic cytochalasans of *Aspergillus flavipes* from the rhizosphere of *Ericameria laricifolia* of the Sonoran Desert. *J. Nat. Prod.* **2004**, *67*, 328–332.
14. Minami, A.; Tajima, N.; Higuchi, Y.; Toyomasu, T.; Sassa, T.; Kato, N.; Dairi, T. Identification and functional analysis of brassicicene C biosynthetic gene cluster in *Alternaria brassicicola*. *Bioorg. Med. Chem. Lett.* **2009**, *19*, 870–874.
15. Kawahara, N.; Sekita, S.; Satake, M. Steroids from *Calvatia cyathiformis*. *Phytochemistry* **1994**, *37*, 213–215, doi:10.1016/0031-9422(94)85028-3.
16. Ngoc Quang, D.; Dinh Bach, D. Ergosta-4,6,8(14),22-tetraen-3-one from Vietnamese *Xylaria* sp. possessing inhibitory activity of nitric oxide production. *Nat. Prod. Res.* **2008**, *22*, 901–906.
17. Gao, N.; Shang, Z.-C.; Yu, P.; Luo, J.; Jian, K.-L.; Kong, L.-Y.; Yang, M.-H. Alkaloids from the endophytic fungus *Penicillium brefeldianum* and their cytotoxic activities. *Chinese Chem. Lett.* **2017**, *28*, 1194–1199.
18. Kusano, M.; Koshino, H.; Uzawa, J.; Fujioka, S.; Kawano, T.; Kimura, Y. Simplicissin, a new pollen growth inhibitor produced by the fungus, *Penicillium* cf. *simplidssimum* (Oudemans) Thorn No. 410. *Biosci. Biotechnol. Biochem.* **1997**, *61*, 2153–2155.
19. Li, J.; Wang, Y.; Hao, X.; Li, S.; Jia, J.; Guan, Y.; Peng, Z.; Bi, H.; Xiao, C.; Cen, S.; et al. Broad-spectrum antiviral natural products from the marine-derived *Penicillium* sp. IMB17-046. *Molecules* **2019**, *24*.
20. Nieto, I.J.; Ávila C, I.M. Determinación de ácidos grasos y compuestos triterpenoides del cuerpo fructífero de *Suillus luteus*. *Rev. Colomb. Quim.* **2008**, *37*, 297–304.
21. Okabe, M.; Sugita, T.; Kinoshita, K.; Koyama, K. Macrolides from a marine-derived fungus, *Penicillium meleagrinum* var. *viridiflavum*, showing synergistic effects with fluconazole against azole-resistant Candida albicans. *J. Nat. Prod.* **2016**, *79*, 1208–1212.
22. Nakano, H.; Hara, M.; Yamashita, Y.; Ando, K.; Shuto, K. Paxisterol, A new analgesic sterol without anti-inflammation activity from *Penicillium*. *J. Antibiot.* **1988**, *41*, 409–410.
23. Che, Y.; Gloer, J.B.; Wicklow, D.T. Curvicollides A−C: New polyketide-derived lactones from a sclerotium-colonizing isolate of *Podospora curvicolla* (NRRL 25778). *Org. Lett.* **2004**, *6*, 1249–1252.

24. Pang, Z.; Sterner, O. The Isolation of Ergosta-4, 6, 8 (14), 22-tetraen-3β-ol from injured fruit bodies of *Marasmius oreades. Nat. Prod. Lett.* **1993**, *3*, 193–196.
25. Matsumura, M.; Miki, S.; Oyama, K.; Tsuchida, M.; Yamamoto, K.; New agricultural/horticultural bactericide 2009, JP2009107953A.
